# Synthesis of 2-Aryl-4*H*-thiochromen-4-one Derivatives *via* a Cross-Coupling Reaction

**DOI:** 10.1021/acsomega.1c01778

**Published:** 2021-05-21

**Authors:** Peng Li, Shengnan Li, Gang Li, Haihong Huang

**Affiliations:** † Beijing Key Laboratory of Active Substance Discovery and Druggability Evaluation, Institute of Materia Medica, Peking Union Medical College and Chinese Academy of Medical Sciences, 1 Xian Nong Tan Street, Beijing 100050, P. R. China; ‡ Chinese Academy of Medical Sciences Key Laboratory of Anti-DR TB Innovative Drug Research, Institute of Materia Medica, Peking Union Medical College and Chinese Academy of Medical Sciences, 1 Xian Nong Tan Street, Beijing 100050, P. R. China

## Abstract

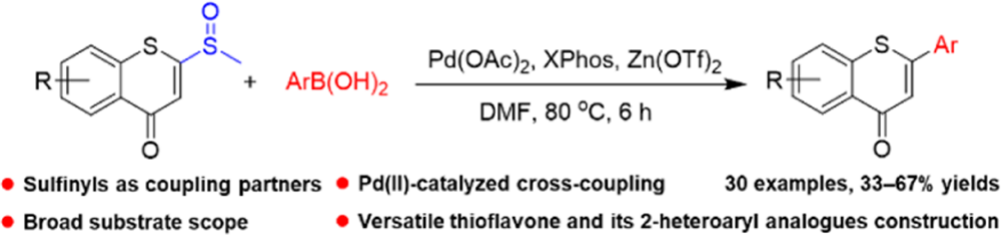

A concise and efficient cross-coupling synthetic strategy has been developed to construct 2-aryl-4*H*-thiochromen-4-one derivatives from 2-sulfinyl-thiochromones and arylboronic acids. This reaction proceeds *via* a catalyst system of Lewis acid and palladium(II) combined with XPhos as an optimal ligand in moderate to good yields. Besides, this flexible methodology provides a wide scope for the synthesis of different functionally substituted thiochromone scaffolds and can be further exploited to construct diverse thioflavone libraries for pharmaceutical research.

## Introduction

**Scheme 1:**
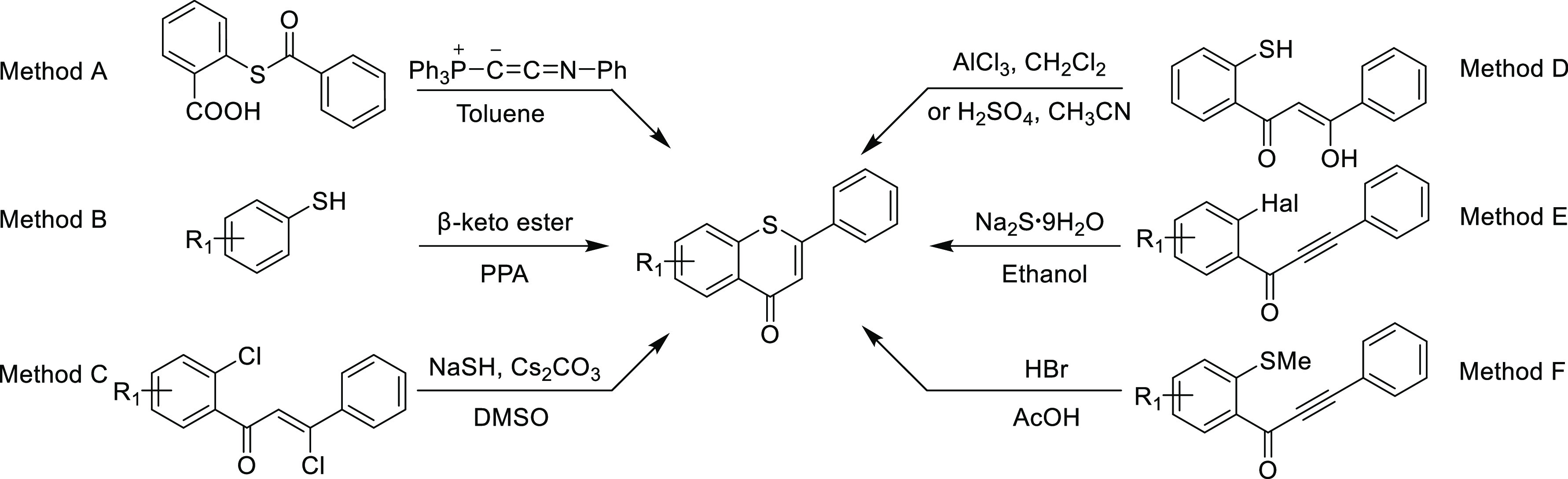
Representative Strategies for Constructing Thioflavones

Thioflavones are an important class of sulfur-containing heterocycles in medicinal chemistry due to their structural similarity to flavones, and this scaffold exhibits various biological and pharmacological properties,^1^ including antibacterial,^2,3^ anticancer,^4−6^ and anti-HIV activities.^7^ However, the synthetic methods for thioflavones *via* cross-coupling reaction are rarely reported. The major common synthetic routes include synthesizing from benzoylthiosalicylic acid *via* intramolecular Wittig cyclization (Scheme 1, method A)^8^ or coupling of thiophenols with β-keto ester in the presence of polyphosphoric acid (Scheme 1, method B).^3,9^ Recent synthetic methods of thioflavone mainly rely on the cyclization of various 2-substituted β-vinyl-aromatic ketones (Scheme 1, methods C and D) or intermolecular Michael addition of β-ethynyl-aromatic ketones (Scheme 1, methods E and F).^10−19^ However, the preparation of various starting materials for current methods limits the rapid synthesis of large libraries of valuable thioflavone precursors for pharmaceutical studies. To the best of our knowledge, there are no reports of the construction of thioflavones *via* cross-coupling reactions using sulfinyls as coupling partners.

Organoboron-mediated cross-coupling is a useful C–C bond-forming reaction. The commercial availability of boron reagents, broad functional group tolerance, and general applicability of the reaction make it suitable for derivative expansion. Flavones can be synthesized by organoboron cross-coupling;^20,21^ especially, the synthesis of flavones *via* transition metal-catalyzed cross-coupling reactions has particularly attracted our attention.^22,23^

**Scheme 2:**
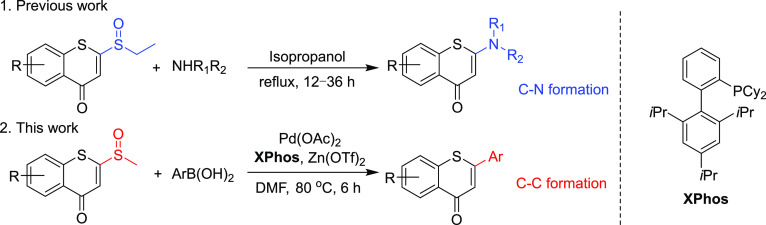
Strategy for Constructing 2-Aryl-thiochromones

Recently, our group has developed an efficient method to synthesize 2-amino-4*H*-benzothiopyran-4-ones from 2-sulfinyl-thiochromones *via* conjugated addition–elimination in which the sulfinyl group worked well as a leaving group (Scheme 2).^24^ The use of sulfinyl and sulfonyl groups in Suzuki–Miyaura cross-coupling encouraged us to consider the potential of 2-sulfinyl-thiochromones as substrates for synthesizing thioflavone analogues.^25,26^ Herein, we report a Lewis acid and Pd(II)-catalyzed cross-coupling method for synthesizing 2-aryl-4*H*-thiochromen-4-one derivatives from 2-sulfinyl-thiochromones in order to expand libraries for biological screening and investigate 2-sulfinyl-thiochromones as important building blocks. This protocol delivers a reliable and concise method for producing thioflavones.

## Results and Discussion

Initially, 2-(methylsulfinyl)-4*H*-thiochromen-4-one (**1a**), which was synthesized according to the reported method,^24^ and phenylboronic acid (**2a**) were chosen as the test substrates for the reaction in the presence of Pd(OAc)_2_ to investigate the feasibility of our method. However, the reaction only offered a trace amount of the desired product **3a** (Table 1, entry 1). Considering that the ligand strongly affects the efficiency of transition-metal catalyzed C–C cross-coupling reactions,^27^ we screened several ligands generally used in Pd(II)-catalyzed cross-coupling reactions. First, monodentate phosphine ligands, such as PPh_3_ (triphenylphosphine) and TFP (tri(2-furyl)phosphine), were investigated, but they showed unsatisfactory yields of 34 and 25%, respectively (Table 1, entries 2 and 3). Next, bidentate phosphine ligands BINAP (2,2′-bis(diphenylphosphino)-1,1′-binaphthalene) and DPEPHOS (bis(2-diphenylphosphinophenyl)ether) were tested, where the yields of 30 and 25% were still not improved significantly (Table 1, entries 4 and 5), though Xantphos (9,9-dimethyl-4,5-bis(diphenylphosphino)xanthene) gave the product in moderate yield (50%) (Table 1, entry 6). Gratifyingly, further screening showed that XPhos (2-(dicyclohexylphosphino)-2′,4′,6′-triisopropylbiphenyl) furnished **3a** in relatively good yield (59%) (Table 1, entry 7). With XPhos as a satisfactory ligand, we then focused on the selection of solvents beyond the commonly used DMF. Replacing DMF with toluene, 1,4-dioxane, and acetonitrile resulted in lower yields of 17–36% (Table 1, entries 8–10). Although the reaction in THF proceeded well (yield 56%, entry 11), difficult-to-remove yellow impurities were mixed with the target product **3a**. Therefore, DMF was confirmed as the optimal solvent. In addition, we also tried to replace Pd(OAc)_2_ with Pd(PPh_3_)_4_, but it only gave a low yield of 25% (Table 1, entry 12). The aforementioned results provided the proof of concept of using Pd(OAc)_2_-catalyzed cross-coupling to synthesize thioflavones from 2-sulfinyl-thiochromones.

**Table 1:**
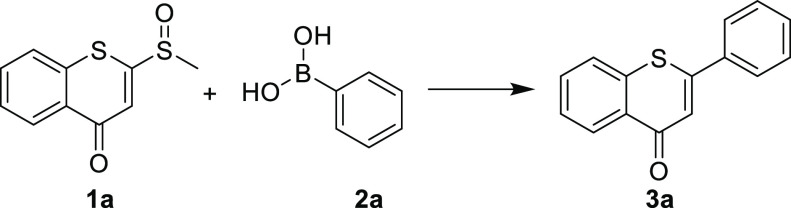
Optimization of the Reaction Conditions^a^

entry	catalyst	ligand	solvent	yield (%)^b^
1	Pd(OAc)_2_		DMF	trace
2	Pd(OAc)_2_	PPh_3_	DMF	34
3	Pd(OAc)_2_	TFP	DMF	25
4	Pd(OAc)_2_	BINAP	DMF	30
5	Pd(OAc)_2_	DPEPHOS	DMF	25
6	Pd(OAc)_2_	Xantphos	DMF	50
7	Pd(OAc)_2_	XPhos	DMF	59
8	Pd(OAc)_2_	XPhos	toluene	36
9	Pd(OAc)_2_	XPhos	1,4-dioxane	31
10	Pd(OAc)_2_	XPhos	acetonitrile	17
11	Pd(OAc)_2_	XPhos	THF	56
12	Pd(PPh_3_)_4_	XPhos	DMF	25

a **1a** (0.5 mmol, 1 equiv), **2a** (2 equiv), catalyst (0.1 equiv), and ligand (0.1 equiv) in solvent (3.0 mL) were stirred at 80 °C for 6 h.

b Isolated yields.

Based on the reported catalytic effect of Lewis acids in the Pd(II)-catalyzed coupling reaction of arylboronic acids with chromones to form flavanones,^28^ subsequently, a variety of Lewis acids were screened to further optimize the reaction condition to construct thioflavones (Table 2). We initially investigated SbCl_3_, which worked well in the Pd(II)-catalyzed coupling of arylboronic acids with α,β-unsaturated ketones;^29^ however, it did not improve the present reaction and gave a yield of 42% only (Table 2, entry 1). We then tested various triflate (OTf)-based Lewis acids.^28^ The results displayed that TMSOTf (trimethylsilyl triflate) and Cu(OTf)_2_ decreased the yields (39–50%) (Table 2, entries 2 and 3), whereas Fe(OTf)_3_ gave a yield of 59%, which was similar to the yield without using Lewis acid (Table 2, entry 4 *vs*Table 1, entry 7). To our delight, both In(OTf)_3_ and Zn(OTf)_2_ increased the yields to 64 and 67%, respectively (Table 2, entries 5 and 6). Considering the cost and availability, Zn(OTf)_2_ was chosen as the Lewis acid for this protocol. We further investigated the solvent impact by replacing DMF with THF under the use of Lewis acid, which resulted in a lower yield (Table 2, entries 6 *vs* 7). Comparing with the reaction without Lewis acid in the absence of the ligand XPhos, Zn(OTf)_2_ improved the reaction yield significantly, exemplified by entry 8 (yield 18%, Table 2) versus entry 1 (trace product, Table 1), which demonstrated the catalytic effects of Lewis acid. In our previously published work, we compared the difference in the reactivity of sulfide, sulfinyl, and sulfonyl groups in the conjugated addition–elimination reaction using 2-sulfinyl-thiochromones to construct 2-amino-4*H*-benzothiopyran-4-ones.^24^ Thus, in this present work, we continuously investigated the effect of different substrates on C–C formation *via* the cross-coupling reaction. Under the same reaction conditions, the sulfinyl as a leaving group still performed the best with the yield of 67% compared to the sulfide and sulfonyl groups with yields of 34 and 12%, respectively (Table 2, entries 6 *vs* 9 and 10). Given the potential irreversible poisoning of transition-metal catalyst by sulfur compounds,^12,30^ the amount of Pd(OAc)_2_ was increased from 0.1 to 0.2 or 0.4 equiv; however, the yields of product **3a** were not significantly improved (Table 2, entries 11 and 12). In a summary, we selected 2-(methylsulfinyl)-4*H*-thiochromen-4-one as the substrate, XPhos (0.1 equiv) as the ligand, Pd(OAc)_2_ (0.1 equiv) as the catalyst, and Zn(OTf)_2_ (0.2 equiv) as the Lewis acid in DMF to further investigate the scope and applications of our approach.

**Table 2:**
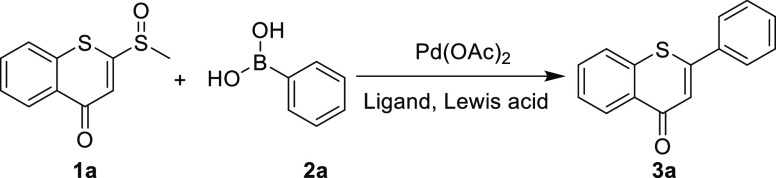
Screening of Lewis Acids for the Cross-Coupling Reaction^a^

entry	ligand	Lewis acid	solvent	yield (%)^b^
1	XPhos	SbCl_3_	DMF	42
2	XPhos	TMSOTf	DMF	50
3	XPhos	Cu(OTf)_2_	DMF	39
4	XPhos	Fe(OTf)_3_	DMF	59
5	XPhos	In(OTf)_3_	DMF	64
6	XPhos	Zn(OTf)_2_	DMF	67
7	XPhos	Zn(OTf)_2_	THF	31
8		Zn(OTf)_2_	DMF	18
9^c^	XPhos	Zn(OTf)_2_	DMF	34
10^d^	XPhos	Zn(OTf)_2_	DMF	12
11^e^	XPhos	Zn(OTf)_2_	DMF	69
12^f^	XPhos	Zn(OTf)_2_	DMF	71

a **1a** (0.5 mmol, 1 equiv), **2a** (2 equiv), Pd(OAc)_2_ (0.1 equiv), XPhos (0.1 equiv), and Lewis acid (0.2 equiv) in solvent (3.0 mL) were stirred at 80 °C for 6 h.

b Isolated yields.

c **1a** was 2-(methylthio)-4*H*-thiochromen-4-one.

d **1a** was 2-(methylsulfonyl)-4*H*-thiochromen-4-one.

e Pd(OAc)_2_ (0.2 equiv).

f Pd(OAc)_2_ (0.4 equiv).

With the optimal conditions in hand, the reactions of various substituted phenylboronic acids with **1a** were tested to explore the functional group compatibility of our method. As summarized in Table 3, the approach tolerated well with a variety of phenylboronic acids substituted by various functional groups, including electron-donating groups (**3b–f**, **3j**, methyl, methoxy, benzyloxy, and hydroxyl, yields 50–67%) and electron-withdrawing groups (**3g–i, 3k, 3l**, halogen, nitro, trifluoromethyl, and methoxycarbonyl, yields 38–65%). Therefore, the compatibility of the reactions with bromine, nitro, hydroxyl, and benzyloxy groups facilitates subsequent structural expansion.

**Table 3:**
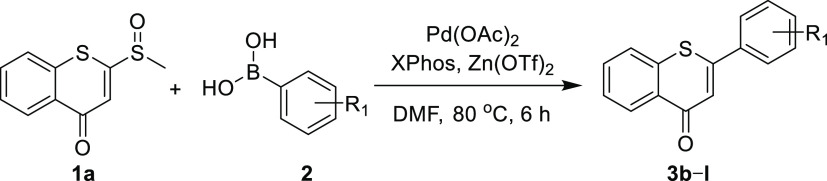

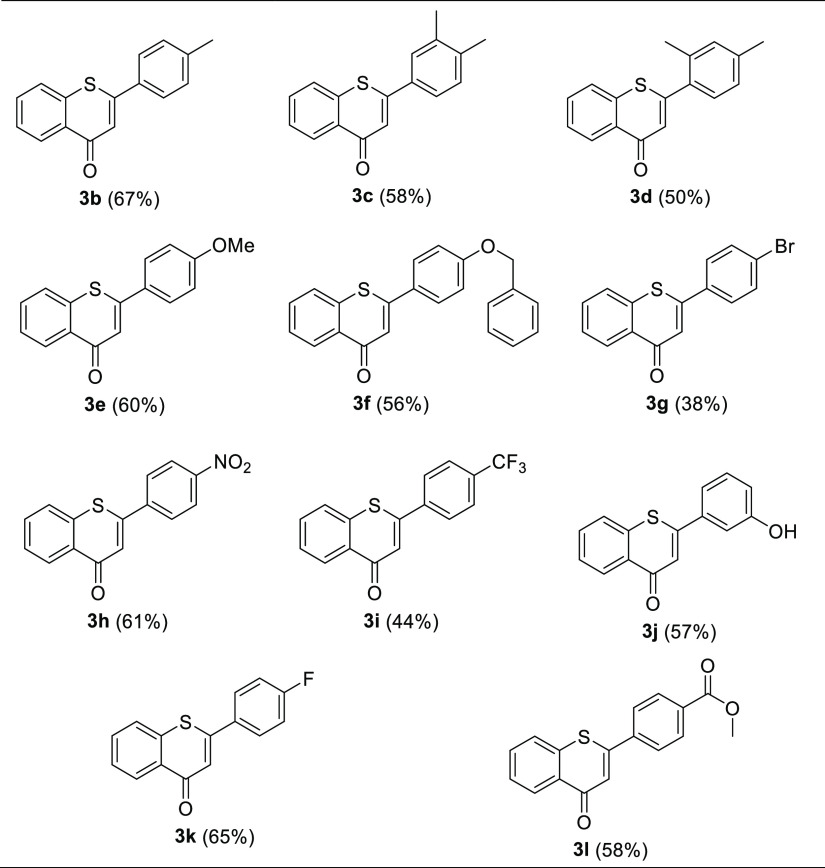
Reaction of 2-Sulfinyl-thiochromone **1a** with Substituted Phenylboronic Acids^a^

a **1a** (0.5 mmol, 1 equiv), substituted phenylboronic acid **2** (2 equiv), Pd(OAc)_2_ (0.1 equiv), XPhos (0.1 equiv), and Zn(OTf)_2_ (0.2 equiv) in DMF (3.0 mL) were stirred at 80 °C for 6 h. Isolated yields are shown in brackets.

Next, we explored the efficiency of the approach on the scope of the substituted 2-sulfinyl-thiochromones (**1**). As shown in Table 4, most 2-sulfinyl-thiochromones (**1**) could be readily transformed into the corresponding thioflavones in moderate to good yields (41–66%), with the electron-donating (**3m–o**) or electron-withdrawing substituents (**3p–u**) on the thiochromone phenyl group. The results indicated that the electronic effect and position of the substituents had less impact on the reactivity. The reaction was also tolerant to nitro and halogen groups, which is convenient for subsequent derivatization.

**Table 4:**
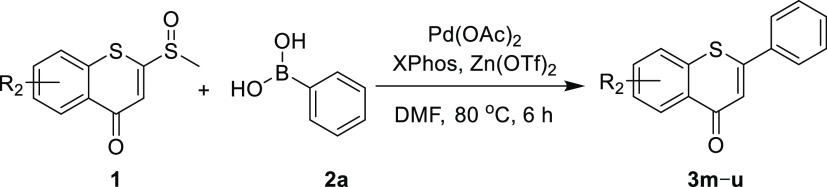

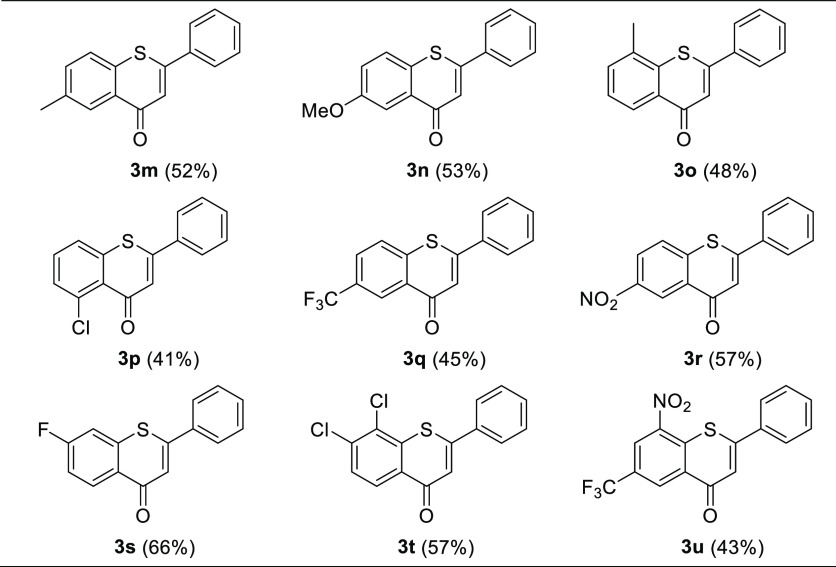
Reaction of Substituted 2-Sulfinyl-thiochromones (**1**) with Phenylboronic Acid (**2a**)^a^

a **1** (0.5 mmol, 1 equiv), **2a** (2 equiv), Pd(OAc)_2_ (0.1 equiv), XPhos (0.1 equiv), and Zn(OTf)_2_ (0.2 equiv) in DMF (3.0 mL) were stirred at 80 °C for 6 h. Isolated yields are shown in brackets.

Finally, to demonstrate the further potential utility of the reaction, the reactivity of substituted 2-sulfinyl-thiochromones with various arylboronic acids was investigated (Table 5). As expected, regardless of the electronic effect of the substituents on the substrates, the corresponding thioflavones (**3v–z**) were obtained with acceptable yields (33–54%). Furthermore, the reaction was extended to the synthesis of 2-heteroaryl analogues, such as the 2-pyridyl (**4a**), 2-furanyl (**4b**), 2-naphthyl (**4c**), and 2-benzothienyl (**4d**) thioflavone derivatives. The results demonstrated that our approach is suitable for preparing 2-aryl-4*H*-thiochromen-4-one derivatives with various functional groups.

**Table 5:**
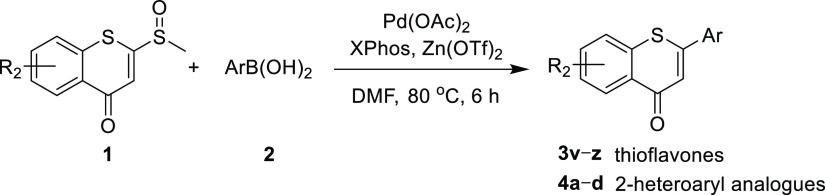

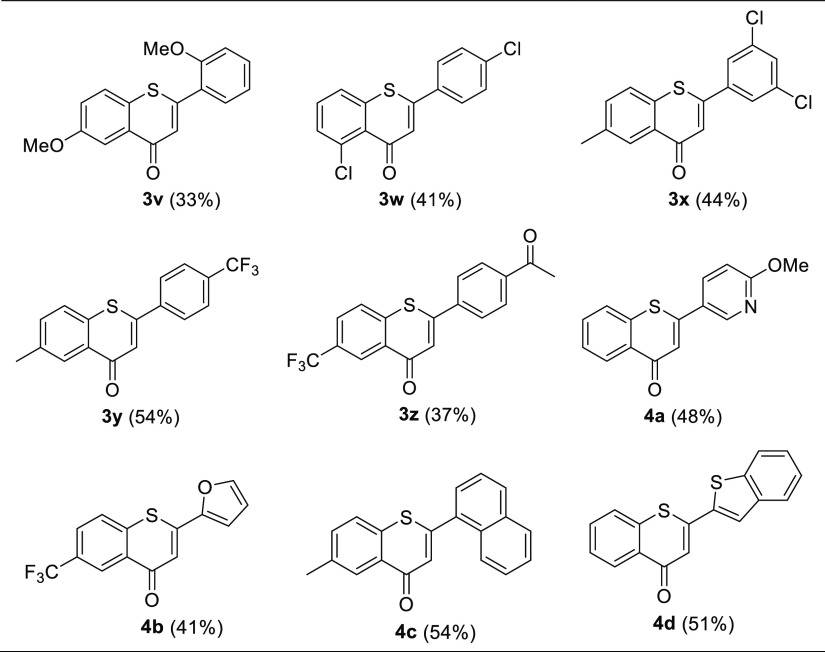
Synthesis of 2-Aryl-4*H*-thiochromen-4-one Derivatives^a^

a **1** (0.5 mmol, 1 equiv), arylboronic acid **2** (2 equiv), Pd(OAc)_2_ (0.1 equiv), XPhos (0.1 equiv), and Zn(OTf)_2_ (0.2 equiv) in DMF (3.0 mL) were stirred at 80 °C for 6 h. Isolated yields are shown in brackets.

**Scheme 3:**
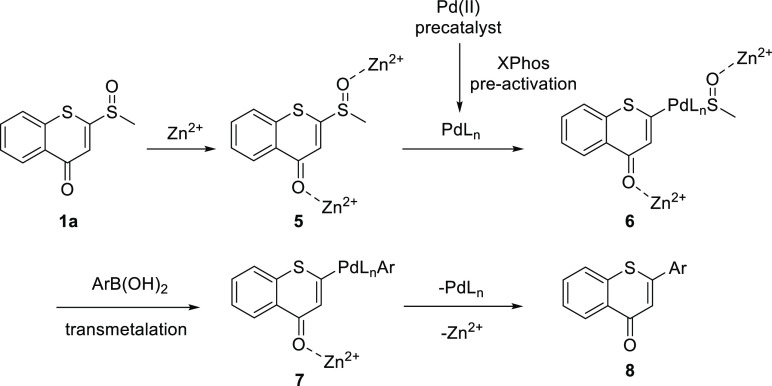
Proposed Mechanism of the Reaction

A plausible mechanism as outlined in Scheme 3 was proposed based on our experimental observations and previously reported literature.^26,28^ Lewis acid Zn(OTf)_2_ may coordinate with the oxygens of carbonyl and sulfinyl, which activates the electrophilicity at C-2 position of the thiochromone (**5**). The oxidative insertion of **5** by Pd(0) which is pre-activated by XPhos from Pd(II) generates thiochromone palladium species **6**. Subsequently, the transmetalation of **6** with arylboronic acid followed by the reductive elimination affords the final product 2-aryl thiochromone **8**.

## Conclusions

In conclusion, we developed a concise and efficient approach to synthesizing 2-aryl-4*H*-thiochromen-4-ones from 2-sulfinyl-thiochromones and arylboronic acids *via* a Lewis acid and Pd(II)-catalyzed cross-coupling reaction. To the best of our knowledge, this is the first use of sulfinyls as coupling partners to construct thioflavones through an organoboron cross-coupling reaction. The reaction exhibits good substituent compatibility and substrate adaptability. Furthermore, our method was extended to the synthesis of 2-heteroaryl thioflavone analogues, exploiting the good availability of heteroarylboronic acids. This strategy provides an effective complementary approach to the existing synthetic methods for thioflavone derivatives, which are used to construct diverse thioflavone libraries for pharmaceutical research.

## Experimental Section

All the solvents and chemicals were obtained from commercial sources and used without further purification. TLC was performed on silica gel plates (GF254) with visualization of components by UV light (254 nm). Column chromatography was carried out on silica gel (200–300 mesh). The structural identities of the prepared compounds were confirmed by ^1^H NMR and ^13^C NMR spectroscopy and high-resolution mass spectrometry (HR-MS). ^1^H NMR spectra were obtained on Varian Mercury 400 at 400 MHz. ^13^C NMR spectra were obtained on Bruker 400 at 100 MHz. Chemical shift values were referenced to the residual solvent peak and reported in ppm (δ scale) and all coupling constant (*J*) values were given in Hz. CDCl_3_ or DMSO-*d*_6_ were used as the standard NMR solvents. The following multiplicity abbreviations are used: (s) singlet, (d) doublet, (t) triplet, (q) quartet, (m) multiplet, and (br) broad. HR-MS (ESI) data were measured on a Thermo Exactive Plus Orbitrap spectrometer. Melting points were determined on a Yanaco MP-J3 microscope melting point apparatus. Substrates **1** were prepared according to the reported procedures.^24^

### General Procedure for the Synthesis of Thioflavones **3a–z** and 2-Heteroaryl Thioflavone Analogues **4a–d**

To a stirred solution of **1** (0.5 mmol, 1.0 equiv) in DMF (3.0 mL) was added Pd(OAc)_2_ (0.05 mmol, 0.1 equiv), XPhos (0.05 mmol, 0.1 equiv), Zn(OTf)_2_ (0.1 mmol, 0.2 equiv), and arylboronic acid **2** (1.0 mmol, 2.0 equiv). The reaction mixture was heated at 80 °C for 6 h. After cooling to room temperature, the solvent was evaporated *in vacuo* and the residue was purified by column chromatography eluting with ethyl acetate (EA)/petroleum (PE) = 1–20:100 to afford the target products.

#### 2-Phenyl-4*H*-thiochromen-4-one (**3a**)

White solid, 80 mg, yield 67%, mp 116–118 °C. ^1^H NMR (400 MHz, CDCl_3_): δ 8.56 (dd, *J* = 8.1, 1.5 Hz, 1H), 7.73–7.68 (m, 2H), 7.68–7.61 (m, 2H), 7.59–7.54 (m, 1H), 7.53–7.49 (m, 3H), 7.28 (s, 1H). ^13^C NMR (100 MHz, CDCl_3_): δ 180.9, 153.2, 137.8, 136.6, 131.6, 130.9, 130.8, 129.3, 128.6, 127.8, 127.0, 126.5, 123.4. HR-MS (ESI) *m*/*z*: [M + H]^+^ calcd for C_15_H_11_OS, 239.0525; found, 239.0529. ^1^H NMR and ^13^C NMR spectra are consistent with the literature.^15^

#### 2-(*p*-Tolyl)-4*H*-thiochromen-4-one (**3b**)

Off-white solid, 84 mg, yield 67%, mp 112–114 °C. ^1^H NMR (400 MHz, CDCl_3_): δ 8.55 (dd, *J* = 8.0, 1.5 Hz, 1H), 7.69–7.58 (m, 4H), 7.58–7.51 (m, 1H), 7.30 (d, *J* = 8.2 Hz, 2H), 7.23 (d, *J* = 1.4 Hz, 1H), 2.43 (s, 3H). ^13^C NMR (100 MHz, CDCl_3_): δ 180.9, 153.1, 141.3, 137.7, 133.7, 131.5, 130.9, 130.0, 128.6, 127.7, 126.8, 126.5, 122.9, 21.4. HR-MS (ESI) *m*/*z*: [M + H]^+^ calcd for C_16_H_13_OS, 253.0682; found, 253.0687. ^1^H NMR and ^13^C NMR spectra are consistent with the literature.^14^

#### 2-(3,4-Dimethylphenyl)-4*H*-thiochromen-4-one (**3c**)

Off-white solid, 77 mg, yield 58%, mp 130–132 °C. ^1^H NMR (400 MHz, CDCl_3_): δ 8.54 (dd, *J* = 7.8, 1.5 Hz, 1H), 7.68–7.58 (m, 2H), 7.57–7.51 (m, 1H), 7.48 (d, *J* = 2.1 Hz, 1H), 7.44 (dd, *J* = 7.8, 2.2 Hz, 1H), 7.24 (d, *J* = 1.7 Hz, 2H), 2.34 (s, 3H), 2.33 (s, 3H). ^13^C NMR (100 MHz, CDCl_3_): δ 180.9, 153.3, 140.1, 137.8, 137.7, 134.1, 131.5, 131.0, 130.5, 128.6, 128.0, 127.6, 126.4, 124.3, 122.7, 19.9, 19.7. HR-MS (ESI) *m*/*z*: [M + H]^+^ calcd for C_17_H_15_OS, 267.0838; found, 267.0844.

#### 2-(2,4-Dimethylphenyl)-4*H*-thiochromen-4-one (**3d**)

Off-white solid, 66 mg, yield 50%, mp 80–82 °C. ^1^H NMR (400 MHz, CDCl_3_): δ 8.58 (d, *J* = 8.1 Hz, 1H), 7.64–7.59 (m, 2H), 7.59–7.53 (m, 1H), 7.27–7.22 (m, 1H), 7.13 (s, 1H), 7.10 (d, *J* = 7.8 Hz, 1H), 6.91 (s, 1H), 2.38 (s, 3H), 2.36 (s, 3H). ^13^C NMR (100 MHz, CDCl_3_): δ 180.6, 153.8, 139.9, 138.5, 135.5, 133.4, 131.7, 131.5, 131.0, 129.0, 128.7, 127.7, 126.8, 126.3, 126.2, 21.2, 19.9. HR-MS (ESI) *m*/*z*: [M + H]^+^ calcd for C_17_H_15_OS, 267.0838; found, 267.0843.

#### 2-(4-Methoxyphenyl)-4*H*-thiochromen-4-one (**3e**)

Off-white solid, 81 mg, yield 60%, mp 115–117 °C. ^1^H NMR (400 MHz, CDCl_3_): δ 8.54 (dd, *J* = 8.0, 1.0 Hz, 1H), 7.70–7.64 (m, 3H), 7.64–7.59 (m, 1H), 7.57–7.52 (m, 1H), 7.22 (s, 1H), 7.02 (d, *J* = 8.9 Hz, 2H), 3.88 (s, 3H). ^13^C NMR (100 MHz, CDCl_3_): δ 180.9, 161.9, 152.9, 137.7, 131.5, 130.9, 128.8, 128.6, 128.4, 127.7, 126.4, 122.2, 114.7, 55.5. HR-MS (ESI) *m*/*z*: [M + H]^+^ calcd for C_16_H_13_O_2_S, 269.0631; found, 269.0635. ^1^H NMR and ^13^C NMR spectra are consistent with the literature.^14^

#### 2-(4-(Benzyloxy)phenyl)-4*H*-thiochromen-4-one (**3f**)

Off-white solid, 97 mg, yield 56%, mp 97–99 °C. ^1^H NMR (400 MHz, CDCl_3_): δ 8.54 (d, *J* = 8.1 Hz, 1H), 7.68–7.63 (m, 3H), 7.61 (t, *J* = 7.3 Hz, 1H), 7.54 (t, *J* = 7.4 Hz, 1H), 7.47–7.38 (m, 4H), 7.38–7.32 (m, 1H), 7.20 (s, 1H), 7.08 (d, *J* = 8.8 Hz, 2H), 5.14 (s, 2H). ^13^C NMR (100 MHz, CDCl_3_): δ 180.9, 161.0, 152.7, 137.6, 136.3, 131.5, 130.9, 129.1, 128.7, 128.6, 128.4, 128.2, 127.7, 127.5, 126.4, 122.2, 115.6, 70.2. HR-MS (ESI) *m*/*z*: [M + H]^+^ calcd for C_22_H_17_O_2_S, 345.0944; found, 345.0949.

#### 2-(4-Bromophenyl)-4*H*-thiochromen-4-one (**3g**)

Off-white solid, 60 mg, yield 38%, mp 154–156 °C. ^1^H NMR (400 MHz, CDCl_3_): δ 8.55 (d, *J* = 8.9 Hz, 1H), 7.69–7.61 (m, 4H), 7.60–7.54 (m, 3H), 7.21 (s, 1H). ^13^C NMR (100 MHz, CDCl_3_): δ 180.7, 151.7, 137.3, 135.5, 132.5, 131.8, 130.8, 128.7, 128.4, 128.0, 126.5, 125.4, 123.6. HR-MS (ESI) *m*/*z*: [M + H]^+^ calcd for C_15_H_10_BrOS, 316.9630; found, 316.9641. ^1^H NMR and ^13^C NMR spectra are consistent with the literature.^11^

#### 2-(4-Nitrophenyl)-4*H*-thiochromen-4-one (**3h**)

Yellow solid, 87 mg, yield 61%, mp 175–177 °C. ^1^H NMR (400 MHz, CDCl_3_): δ 8.57 (d, *J* = 7.9 Hz, 1H), 8.37 (d, *J* = 8.8 Hz, 2H), 7.87 (d, *J* = 8.8 Hz, 2H), 7.71–7.67 (m, 2H), 7.63–7.58 (m, 1H), 7.27 (s, 1H). ^13^C NMR (100 MHz, CDCl_3_): δ 180.5, 150.0, 149.1, 142.5, 137.0, 132.1, 130.8, 128.8, 128.3, 128.1, 126.6, 125.0, 124.5. HR-MS (ESI) *m*/*z*: [M + H]^+^ calcd for C_15_H_10_NO_3_S, 284.0376; found, 284.0381. ^1^H NMR and ^13^C NMR spectra are consistent with the literature.^31^

#### 2-(4-(Trifluoromethyl)phenyl)-4*H*-thiochromen-4-one (**3i**)

Off-white solid, 68 mg, yield 44%, mp 167–169 °C. ^1^H NMR (400 MHz, CDCl_3_): δ 8.56 (d, *J* = 8.0 Hz, 1H), 7.79 (q, *J* = 8.3 Hz, 4H), 7.70–7.63 (m, 2H), 7.58 (t, *J* = 7.2 Hz, 1H), 7.25 (s, 1H). ^13^C NMR (100 MHz, CDCl_3_): δ 180.6, 151.1, 140.0, 137.3, 132.6 (q, ^2^*J*_C,F_ = 33 Hz), 131.9, 130.9, 128.7, 128.1, 127.5, 126.6, 126.3, 124.4, 123.7 (q, ^1^*J*_C,F_ = 271 Hz). HR-MS (ESI) *m*/*z*: [M + H]^+^ calcd for C_16_H_10_F_3_OS, 307.0399; found, 307.0406. ^1^H NMR and ^13^C NMR spectra are consistent with the literature.^11^

#### 2-(3-Hydroxyphenyl)-4*H*-thiochromen-4-one (**3j**)

Purified by column chromatography with ethyl acetate (EA)/petroleum (PE) = 1–30:100. Off-white solid, 73 mg, yield 57%, mp 179–181 °C. ^1^H NMR (400 MHz, DMSO-*d*_6_): δ 9.95 (brs, 1H), 8.37 (d, *J* = 8.1 Hz, 1H), 7.95 (d, *J* = 8.2 Hz, 1H), 7.79 (t, *J* = 7.6 Hz, 1H), 7.66 (t, *J* = 7.6 Hz, 1H), 7.38 (t, *J* = 7.9 Hz, 1H), 7.24 (d, *J* = 7.8 Hz, 1H), 7.18 (s, 1H), 7.16 (t, *J* = 2.1 Hz, 1H), 6.99 (dd, *J* = 8.1, 2.7 Hz, 1H). ^13^C NMR (100 MHz, DMSO-*d*_6_): δ 179.3, 158.0, 152.1, 136.9, 136.7, 132.1, 130.6, 130.1, 128.1, 127.6, 127.1, 122.2, 118.0, 117.3, 113.2. HR-MS (ESI) *m*/*z*: [M + H]^+^ calcd for C_15_H_11_O_2_S, 255.0474; found, 255.0476.

#### 2-(4-Fluorophenyl)-4*H*-thiochromen-4-one (**3k**)

Off-white solid, 83 mg, yield 65%, mp 157–159 °C. ^1^H NMR (400 MHz, CDCl_3_): δ 8.57–8.51 (m, 1H), 7.72–7.61 (m, 4H), 7.59–7.53 (m, 1H), 7.24–7.16 (m, 3H). ^13^C NMR (100 MHz, CDCl_3_): δ 180.8, 164.3 (d, ^1^*J*_C,F_ = 250 Hz), 151.8, 137.4, 132.7, 131.7, 130.8, 129.0 (d, ^3^*J*_C,F_ = 9 Hz), 128.7, 127.9, 126.5, 123.5, 116.5 (d, ^2^*J*_C,F_ = 22 Hz). HR-MS (ESI) *m*/*z*: [M + H]^+^ calcd for C_15_H_10_FOS, 257.0431; found, 257.0433. ^1^H NMR and ^13^C NMR spectra are consistent with the literature.^13^

#### 2-(4-Methoxycarbonylphenyl)-4*H*-thiochromen-4-one (**3l**)

Off-white solid, 86 mg, yield 58%, mp 144–146 °C. ^1^H NMR (400 MHz, CDCl_3_): δ 8.56 (d, *J* = 7.3 Hz, 1H), 8.17 (d, *J* = 8.7 Hz, 2H), 7.77 (d, *J* = 8.8 Hz, 2H), 7.69–7.62 (m, 2H), 7.60–7.55 (m, 1H), 7.28 (s, 1H), 3.97 (s, 3H). ^13^C NMR (100 MHz, CDCl_3_): δ 180.7, 166.2, 151.6, 140.7, 137.4, 132.1, 131.8, 130.9, 130.5, 130.2, 128.7, 128.0, 127.3, 127.0, 126.6, 124.2, 52.5. HR-MS (ESI) *m*/*z*: [M + H]^+^ calcd for C_17_H_13_O_3_S, 297.0580; found, 297.0587.

#### 6-Methyl-2-phenyl-4*H*-thiochromen-4-one (**3m**)

Off-white solid, 66 mg, yield 52%, mp 143–145 °C. ^1^H NMR (400 MHz, CDCl_3_): δ 8.37 (d, *J* = 1.9 Hz, 1H), 7.72–7.68 (m, 2H), 7.58 (d, *J* = 8.2 Hz, 1H), 7.53–7.49 (m, 3H), 7.47 (dd, *J* = 8.3, 2.0 Hz, 1H), 7.26 (s, 1H), 2.51 (s, 3H). ^13^C NMR (100 MHz, CDCl_3_): δ 180.9, 153.1, 138.2, 136.7, 134.8, 133.1, 130.8, 130.7, 129.3, 128.4, 127.0, 126.4, 123.3, 21.3. HR-MS (ESI) *m*/*z*: [M + H]^+^ calcd for C_16_H_13_OS, 253.0682; found, 253.0688. ^1^H NMR and ^13^C NMR spectra are consistent with the literature.^13^

#### 6-Methoxy-2-phenyl-4*H*-thiochromen-4-one (**3n**)

Off-white solid, 71 mg, yield 53%, mp 145–147 °C. ^1^H NMR (400 MHz, CDCl_3_): δ 8.25 (d, *J* = 6.5 Hz, 1H), 8.00 (d, *J* = 2.9 Hz, 1H), 7.72–7.67 (m, 2H), 7.58 (d, *J* = 8.8 Hz, 1H), 7.54–7.47 (m, 3H), 7.29–7.24 (m, 1H), 3.95 (s, 3H). ^13^C NMR (100 MHz, CDCl_3_): δ 180.6, 159.6, 153.2, 136.7, 135.6, 130.7, 129.3, 128.0, 127.8, 127.0, 122.6, 122.2, 108.8, 55.8. HR-MS (ESI) *m*/*z*: [M + H]^+^ calcd for C_16_H_13_O_2_S, 269.0631; found, 269.0637.

#### 8-Methyl-2-phenyl-4*H*-thiochromen-4-one (**3o**)

Off-white solid, 61 mg, yield 48%, mp 114–116 °C. ^1^H NMR (400 MHz, CDCl_3_): δ 8.45 (dd, *J* = 7.6, 2.1 Hz, 1H), 7.76–7.71 (m, 2H), 7.55–7.43 (m, 5H), 7.28–7.25 (m, 1H), 2.59 (s, 3H). ^13^C NMR (100 MHz, CDCl_3_): δ 181.4, 152.2, 137.2, 136.9, 134.7, 132.8, 131.2, 130.8, 129.3, 127.1, 127.1, 126.5, 123.3, 19.6. HR-MS (ESI) *m*/*z*: [M + H]^+^ calcd for C_16_H_13_OS, 253.0682; found, 253.0687. ^1^H NMR and ^13^C NMR spectra are consistent with the literature.^12^

#### 5-Chloro-2-phenyl-4*H*-thiochromen-4-one (**3p**)

Yellow solid, 56 mg, yield 41%, mp 124–126 °C. ^1^H NMR (400 MHz, CDCl_3_): δ 7.70–7.66 (m, 2H), 7.59–7.54 (m, 1H), 7.54–7.50 (m, 4H), 7.47 (d, *J* = 7.9 Hz, 1H), 7.18 (s, 1H). ^13^C NMR (100 MHz, CDCl_3_): δ 180.4, 149.8, 140.7, 136.2, 135.7, 131.5, 131.0, 129.3, 127.8, 126.9, 126.8, 125.6, 124.7. HR-MS (ESI) *m*/*z*: [M + H]^+^ calcd for C_15_H_10_ClOS, 273.0135; found, 273.0141.

#### 2-Phenyl-6-(trifluoromethyl)-4*H*-thiochromen-4-one (**3q**)

Off-white solid, 69 mg, yield 45%, mp 159–161 °C. ^1^H NMR (400 MHz, CDCl_3_): δ 8.82 (d, *J* = 2.0 Hz, 1H), 7.80 (q, *J* = 8.5 Hz, 2H), 7.72–7.66 (m, 2H), 7.57–7.49 (m, 3H), 7.27 (s, 1H). ^13^C NMR (100 MHz, CDCl_3_): δ 179.8, 153.2, 141.2, 136.0, 131.3, 131.0, 130.1 (q, ^2^*J*_C,F_ = 33 Hz), 129.5, 127.6, 127.5, 127.0, 126.1, 123.6, 123.5 (q, ^1^*J*_C,F_ = 271 Hz). HR-MS (ESI) *m*/*z*: [M + H]^+^ calcd for C_16_H_10_F_3_OS, 307.0399; found, 307.0405. ^1^H NMR and ^13^C NMR spectra are consistent with the literature.^32^

#### 6-Nitro-2-phenyl-4*H*-thiochromen-4-one (**3r**)

Yellow solid, 81 mg, yield 57%, mp 180–182 °C. ^1^H NMR (400 MHz, CDCl_3_): δ 9.35 (d, *J* = 2.5 Hz, 1H), 8.42 (dd, *J* = 8.8, 2.6 Hz, 1H), 7.83 (d, *J* = 8.8 Hz, 1H), 7.73–7.67 (m, 2H), 7.59–7.51 (m, 3H), 7.28 (s, 1H). ^13^C NMR (100 MHz, CDCl_3_): δ 179.3, 153.0, 147.2, 144.0, 135.6, 131.5, 131.4, 129.5, 128.0, 127.0, 125.3, 124.3, 123.5. HR-MS (ESI) *m*/*z*: [M + H]^+^ calcd for C_15_H_10_NO_3_S, 284.0376; found, 284.0380.

#### 7-Fluoro-2-phenyl-4*H*-thiochromen-4-one (**3s**)

Off-white solid, 84 mg, yield 66%, mp 126–128 °C. ^1^H NMR (400 MHz, CDCl_3_): δ 8.57 (dd, *J* = 9.0, 5.9 Hz, 1H), 7.71–7.65 (m, 2H), 7.54–7.48 (m, 3H), 7.35 (dd, *J* = 8.5, 2.5 Hz, 1H), 7.26 (td, *J* = 8.4, 2.4 Hz, 1H), 7.21 (s, 1H). ^13^C NMR (100 MHz, CDCl_3_): δ 179.9, 164.1 (d, ^1^*J*_C,F_ = 250 Hz), 152.7, 139.9, 136.2, 131.8, 131.0, 129.4, 127.6, 127.0, 123.5, 116.4 (d, *J* = 22 Hz), 112.3 (d, ^2^*J*_C,F_ = 25 Hz).^12^ HR-MS (ESI) *m*/*z*: [M + H]^+^ calcd for C_15_H_10_FOS, 257.0431; found, 257.0435.

#### 7,8-Dichloro-2-phenyl-4*H*-thiochromen-4-one (**3t**)

Yellow solid, 88 mg, yield 57%, mp 215–217 °C. ^1^H NMR (400 MHz, CDCl_3_): δ 8.44 (d, *J* = 8.7 Hz, 1H), 7.75–7.71 (m, 2H), 7.65 (d, *J* = 8.7 Hz, 1H), 7.56–7.52 (m, 3H), 7.24 (s, 1H). ^13^C NMR (100 MHz, CDCl_3_): δ 180.2, 153.2, 138.9, 137.2, 136.3, 131.2, 131.1, 129.4, 128.9, 128.8, 128.0, 127.1, 123.0. HR-MS (ESI) *m*/*z*: [M + H]^+^ calcd for C_15_H_9_Cl_2_OS, 306.9746; found, 306.9760.

#### 8-Nitro-2-phenyl-6-(trifluoromethyl)-4*H*-thiochromen-4-one (**3u**)

Yellow solid, 76 mg, yield 43%, mp 207–209 °C. ^1^H NMR (400 MHz, CDCl_3_): δ 9.21 (d, *J* = 2.2 Hz, 1H), 8.90 (d, *J* = 2.2 Hz, 1H), 7.74 (d, *J* = 6.1 Hz, 2H), 7.63–7.53 (m, 3H), 7.29 (s, 1H). ^13^C NMR (100 MHz, CDCl_3_): δ 178.2, 155.1, 145.0, 138.1, 135.7, 134.1, 132.4, 131.7, 129.6, 129.4 (q, ^2^*J*_C,F_ = 35 Hz) 127.3, 126.2, 123.2, 122.5 (q, ^1^*J*_C,F_ = 271 Hz). HR-MS (ESI) *m*/*z*: [M + H]^+^ calcd for C_16_H_9_F_3_NO_3_S, 352.0250; found, 352.0250.

#### 6-Methoxy-2-(2-methoxyphenyl)-4*H*-thiochromen-4-one (**3v**)

Off-white solid, 49 mg, yield 33%, mp 130–132 °C. ^1^H NMR (400 MHz, CDCl_3_): δ 8.01 (d, *J* = 2.9 Hz, 1H), 7.54 (d, *J* = 8.8 Hz, 1H), 7.47–7.42 (m, 2H), 7.26–7.23 (m, 1H), 7.17 (s, 1H), 7.08–7.00 (m, 2H), 3.95 (s, 3H), 3.86 (s, 3H). ^13^C NMR (100 MHz, CDCl_3_): δ 180.4, 159.4, 156.5, 150.2, 132.3, 131.5, 130.9, 130.3, 127.6, 125.9, 125.6, 122.0, 120.9, 111.7, 108.7, 55.8, 55.7. HR-MS (ESI) *m*/*z*: [M + H]^+^ calcd for C_17_H_15_O_3_S, 299.0736; found, 299.0739.

#### 5-Chloro-2-(4-chlorophenyl)-4*H*-thiochromen-4-one (**3w**)

Yellow solid, 63 mg, yield 41%, mp 191–193 °C. ^1^H NMR (400 MHz, CDCl_3_): δ 7.61 (d, *J* = 8.6 Hz, 2H), 7.58–7.51 (m, 2H), 7.50–7.45 (m, 3H), 7.13 (s, 1H). ^13^C NMR (100 MHz, CDCl_3_): δ 180.3, 148.4, 140.3, 137.3, 136.3, 134.1, 131.7, 131.1, 129.6, 128.1, 125.6, 124.8, 116.8. HR-MS (ESI) *m*/*z*: [M + H]^+^ calcd for C_15_H_9_Cl_2_OS, 306.9746; found, 306.9755.

#### 2-(3,5-Dichlorophenyl)-6-methyl-4*H*-thiochromen-4-one (**3x**)

Off-white solid, 70 mg, yield 44%, mp 224–226 °C. ^1^H NMR (400 MHz, CDCl_3_): δ 8.36 (s, 1H), 7.59–7.55 (m, 3H), 7.51–7.47 (m, 2H), 7.18 (s, 1H), 2.51 (s, 3H). ^13^C NMR (100 MHz, CDCl_3_): δ 180.6, 149.9, 139.5, 138.7, 136.0, 134.1, 133.4, 130.6, 130.5, 128.5, 126.4, 125.5, 124.2, 21.4. HR-MS (ESI) *m*/*z*: [M + H]^+^ calcd for C_16_H_11_Cl_2_OS, 320.9902; found, 320.9909.

#### 6-Methyl-2-(4-(trifluoromethyl)phenyl)-4*H*-thiochromen-4-one (**3y**)

Off-white solid, 87 mg, yield 54%, mp 151–153 °C. ^1^H NMR (400 MHz, CDCl_3_): δ 8.38 (d, *J* = 2.0 Hz, 1H), 7.79 (q, *J* = 8.4 Hz, 4H), 7.59 (d, *J* = 8.2 Hz, 1H), 7.49 (dd, *J* = 8.2, 2.1 Hz, 1H), 7.25 (s, 1H), 2.51 (s, 3H). ^13^C NMR (100 MHz, CDCl_3_): δ 180.8, 151.3, 140.1, 138.6, 134.3, 133.4, 132.6 (q, ^2^*J*_C,F_ = 33 Hz), 130.6, 128.5, 127.5, 127.1, 126.4, 126.3, 124.2, 123.7 (q, ^1^*J*_C,F_ = 271 Hz), 115.6, 21.4. HR-MS (ESI) *m*/*z*: [M + H]^+^ calcd for C_17_H_12_F_3_OS, 321.0556; found, 321.0561.

#### 2-(4-Acetylphenyl)-6-(trifluoromethyl)-4*H*-thiochromen-4-one (**3z**)

Off-white solid, 65 mg, yield 37%, mp 162–164 °C. ^1^H NMR (400 MHz, CDCl_3_): δ 8.83 (s, 1H), 8.10 (d, *J* = 8.5 Hz, 2H), 7.89–7.83 (m, 1H), 7.83–7.77 (m, 3H), 7.31 (s, 1H), 2.67 (s, 3H). ^13^C NMR (100 MHz, CDCl_3_): δ 197.1, 179.7, 151.8, 140.1, 138.9, 130.9, 130.4 (q, ^2^*J*_C,F_ = 34 Hz), 129.3, 129.0, 127.9, 127.5, 127.4, 127.3, 126.2, 124.5, 123.5 (q, ^1^*J*_C,F_ = 271 Hz), 115.4, 26.8. HR-MS (ESI) *m*/*z*: [M + H]^+^ calcd for C_18_H_12_F_3_O_2_S, 349.0505; found, 349.0508.

#### 2-(6-Methoxypyridin-3-yl)-4*H*-thiochromen-4-one (**4a**)

White solid, 64 mg, yield 48%, mp 135–137 °C. ^1^H NMR (400 MHz, CDCl_3_): δ 8.57–8.51 (m, 2H), 7.88 (dd, *J* = 8.7, 2.7 Hz, 1H), 7.70–7.60 (m, 2H), 7.59–7.54 (m, 1H), 7.18 (s, 1H), 6.88 (d, *J* = 8.7 Hz, 1H), 4.02 (s, 3H). ^13^C NMR (100 MHz, CDCl_3_): δ 180.7, 165.8, 149.8, 145.5, 137.3, 136.9, 131.7, 130.9, 128.7, 127.9, 126.4, 125.9, 122.8, 111.6, 54.0. HR-MS (ESI) *m*/*z*: [M + H]^+^ calcd for C_15_H_12_NO_2_S, 270.0583; found, 270.0588.

#### 2-(Furan-2-yl)-6-(trifluoromethyl)-4*H*-thiochromen-4-one (**4b**)

Yellow solid, 61 mg, yield 41%, mp 161–163 °C. ^1^H NMR (400 MHz, CDCl_3_): δ 8.77 (s, 1H), 7.80 (dd, *J* = 8.4, 2.1 Hz, 1H), 7.72 (d, *J* = 8.5 Hz, 1H), 7.64 (d, *J* = 1.7 Hz, 1H), 7.35 (s, 1H), 6.99 (d, *J* = 3.8 Hz, 1H), 6.60 (dd, *J* = 3.6, 1.8 Hz, 1H). ^13^C NMR (100 MHz, CDCl_3_): δ 179.4, 148.5, 145.8, 140.8, 139.9, 131.2, 130.1 (q, ^2^*J*_C,F_ = 33 Hz), 127.6, 127.4, 126.0, 123.5 (q, ^1^*J*_C,F_ = 270 Hz), 118.9, 112.9, 112.1. HR-MS (ESI) *m*/*z*: [M + H]^+^ calcd for C_14_H_8_F_3_O_2_S, 297.0192; found, 297.0197.

#### 6-Methyl-2-(naphthalen-1-yl)-4*H*-thiochromen-4-one (**4c**)

Yellow solid, 81 mg, yield 54%, mp 158–160 °C. ^1^H NMR (400 MHz, CDCl_3_): δ 8.45 (s, 1H), 8.07 (d, *J* = 9.0 Hz, 1H), 7.97 (d, *J* = 8.1 Hz, 1H), 7.93 (dd, *J* = 7.2, 2.5 Hz, 1H), 7.63–7.46 (m, 6H), 7.16 (s, 1H), 2.54 (s, 3H). ^13^C NMR (100 MHz, CDCl_3_): δ 180.4, 152.3, 138.3, 135.7, 134.2, 133.7, 133.1, 130.9, 130.6, 130.4, 128.5, 128.4, 127.2, 127.2, 127.1, 126.6, 126.1, 125.0, 124.9, 21.4. HR-MS (ESI) *m*/*z*: [M + H]^+^ calcd for C_20_H_15_OS, 303.0838; found, 303.0846.

#### 2-(Benzo[*b*]thiophen-2-yl)-4*H*-thiochromen-4-one (**4d**)

Yellow solid, 75 mg, yield 51%, mp 157–159 °C. ^1^H NMR (400 MHz, CDCl_3_): δ 8.53 (d, *J* = 8.4 Hz, 1H), 7.89–7.83 (m, 2H), 7.81 (s, 1H), 7.66–7.63 (m, 2H), 7.58–7.53 (m, 1H), 7.45–7.41 (m, 2H), 7.34 (s, 1H). ^13^C NMR (100 MHz, CDCl_3_) δ 180.6, 145.5, 140.2, 139.5, 138.8, 136.8, 131.9, 131.1, 128.7, 127.9, 126.5, 126.3, 125.3, 124.7, 124.3, 122.9, 122.5. HR-MS (ESI) *m*/*z*: [M + H]^+^ calcd for C_17_H_11_OS_2_, 295.0246; found, 295.0256.

## Supporting Information Available

Copies of ^1^H NMR and ^13^C NMR spectra for all compounds (PDF)
