# Supplementary material for: Synthesis of 2-Aryl-4H-thiochromen-4-one Derivatives via a Cross-Coupling Reaction
Source: ACS Omega. 2021 May 21;6(22):14655–63. doi: 10.1021/acsomega.1c01778 (PMC8190924; doi:10.1021/acsomega.1c01778)
Supplement: Supplementary file 1 — ao1c01778_si_001.pdf [file ao1c01778_si_001.pdf]

Supporting Information

Synthesis of 2-aryl-4*H*-thiochromen-4-one derivatives *via* a cross-coupling reaction

Peng Li[1,2], Shengnan Li[1], Gang Li[1,2,*], Haihong Huang[1,2,*]

[1] Beijing Key Laboratory of Active Substance Discovery and Druggability Evaluation, Institute of Materia Medica, Peking Union Medical College and Chinese Academy of Medical Sciences, 1 Xian Nong Tan Street, Beijing 100050, P. R. China

[2] Chinese Academy of Medical Sciences Key Laboratory of Anti-DR TB Innovative Drug Research, Institute of Materia Medica, Peking Union Medical College and Chinese Academy of Medical Sciences, 1 Xian Nong Tan Street, Beijing 100050, P. R. China

E-mail addresses: ligang@imm.ac.cn, joyce@imm.ac.cn

Contents:

^{1}H NMR and ^{13}C NMR of compound **3a~z**……………………………………….....S2-27

^{1}H NMR and ^{13}C NMR of compound **4a~d**…………………………..………………...S28-31

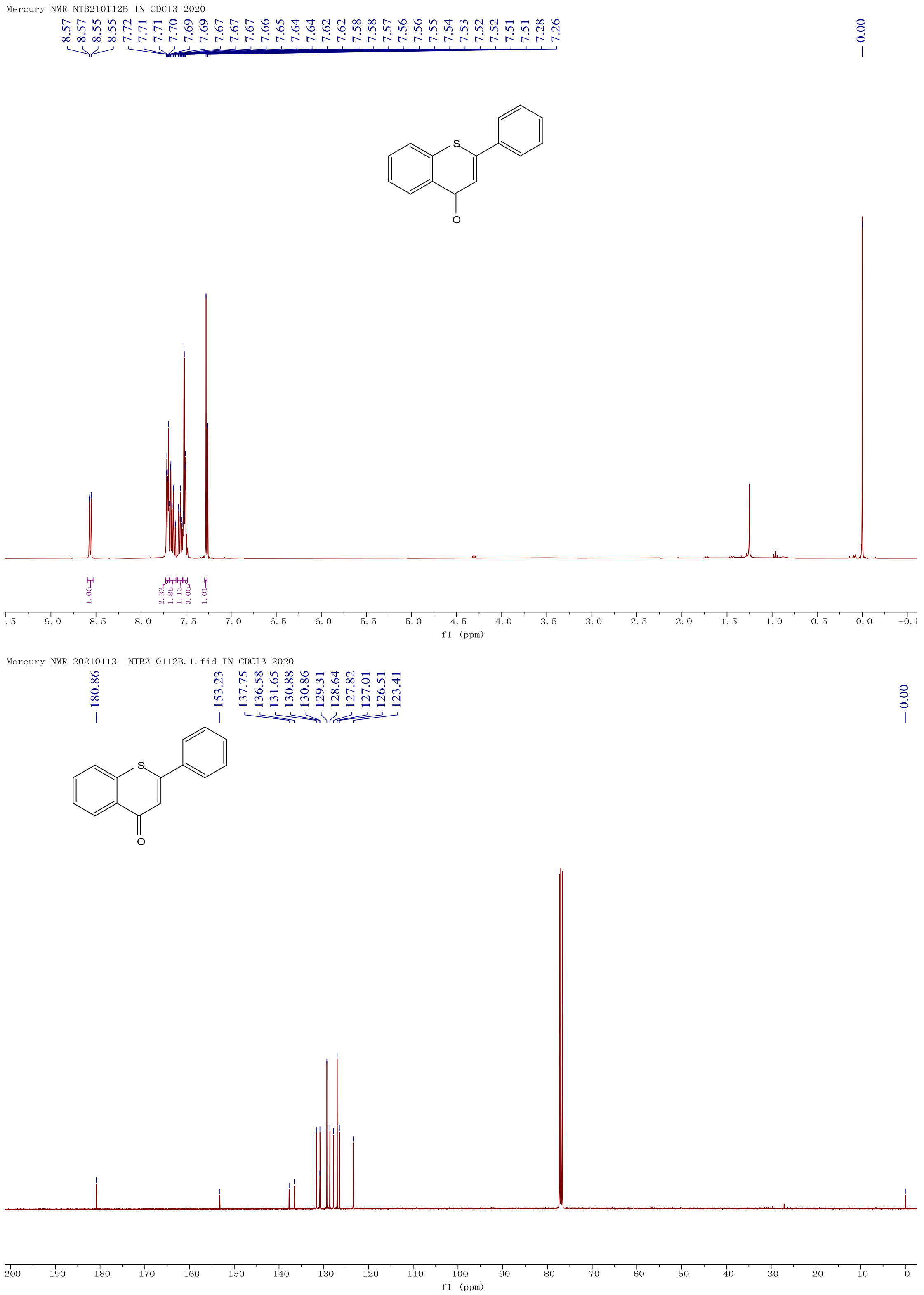

Figure S1. ^{1}H and ^{13}C NMR spectra of 2-Phenyl-4*H*-thiochromen-4-one (**3a**)

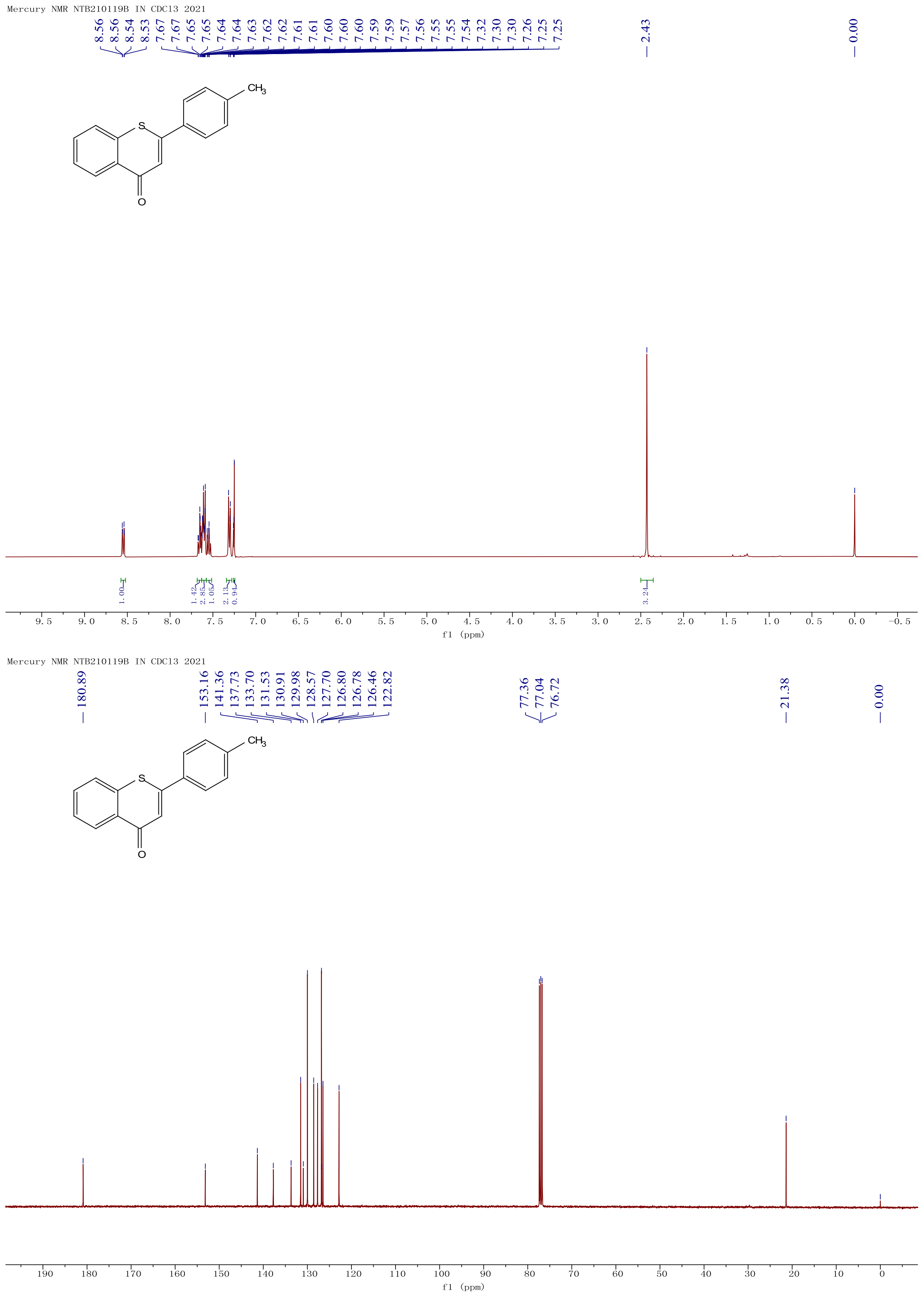

Figure S2. ^{1}H and ^{13}C NMR spectra of 2-(p-Tolyl)-4*H*-thiochromen-4-one (**3b**)

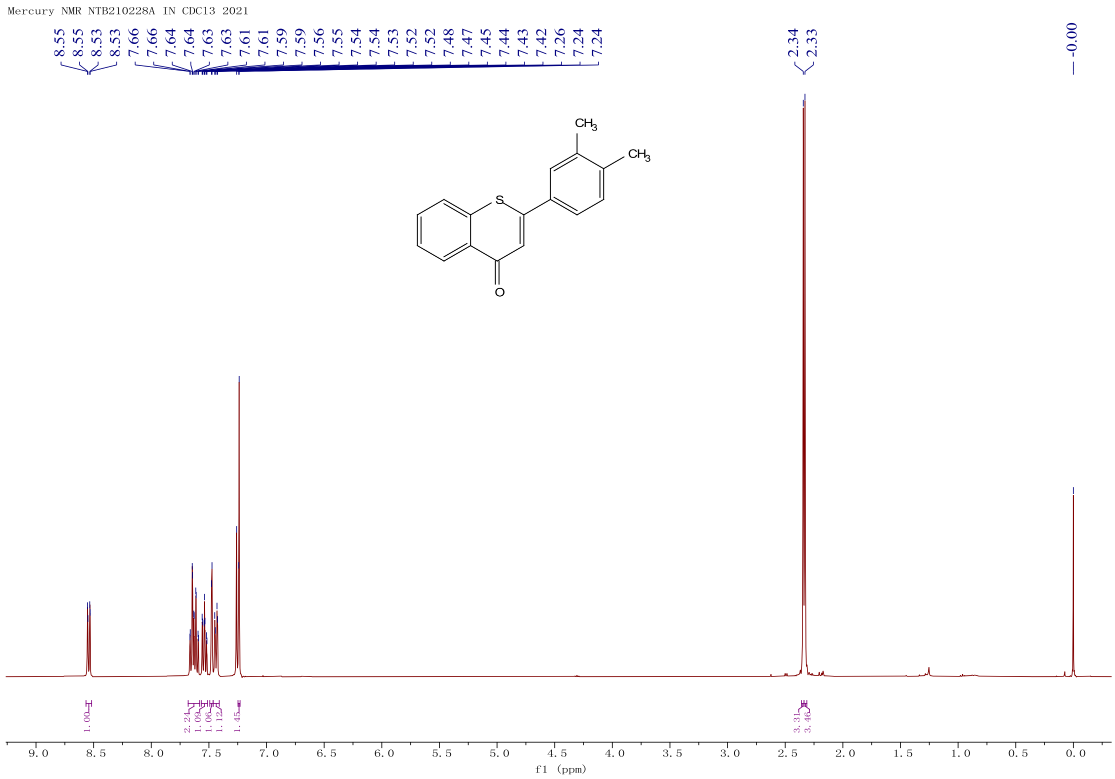

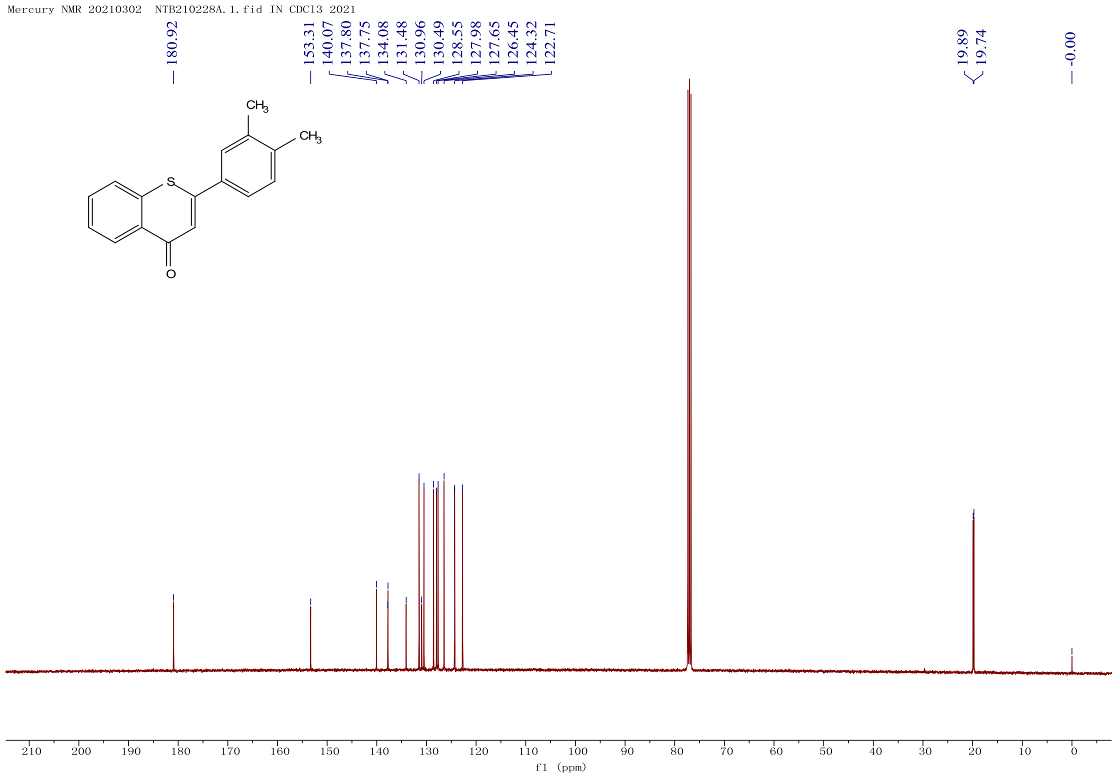

Figure S3. ^{1}H and ^{13}C NMR spectra of 2-(3,4-Dimethylphenyl)-4*H*-thiochromen-4-one (**3c**)

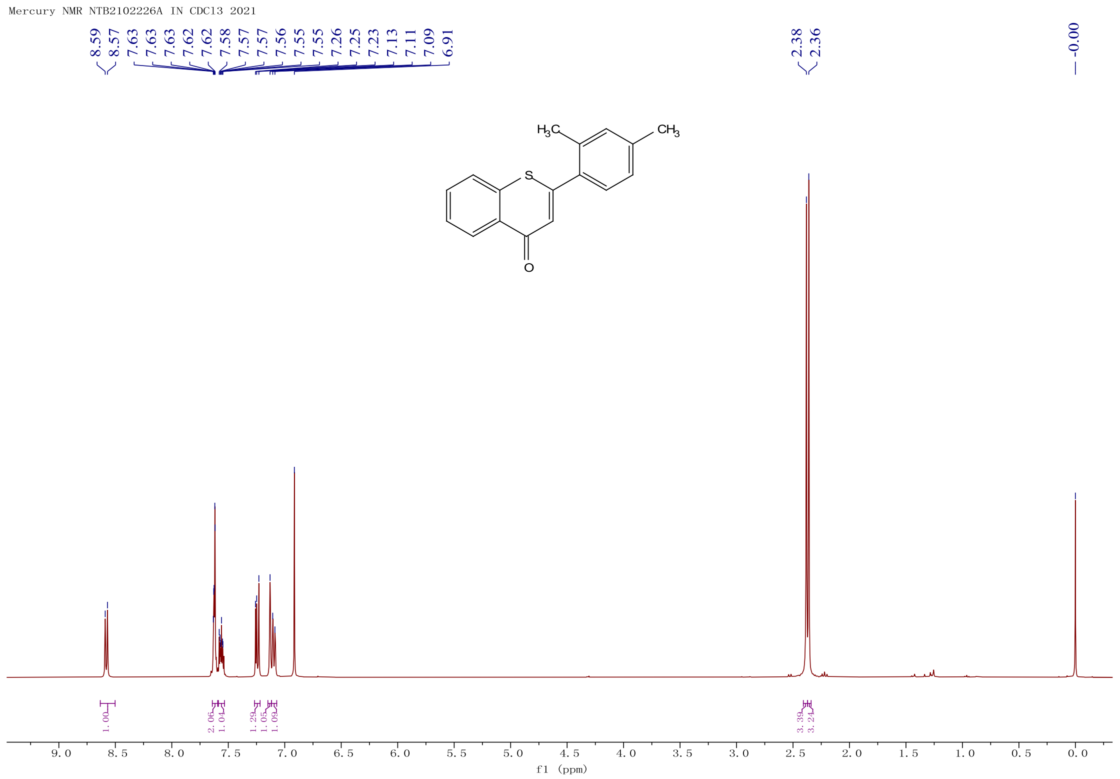

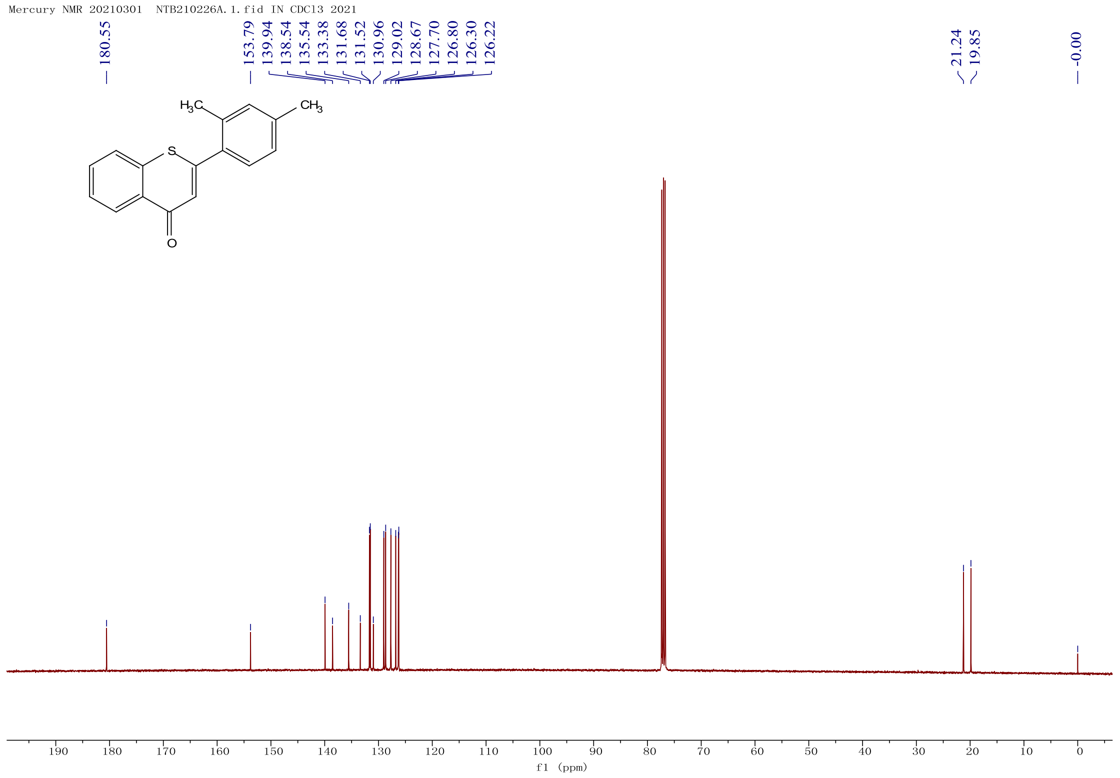

Figure S4. ^{1}H and ^{13}C NMR spectra of 2-(2,4-Dimethylphenyl)-4*H*-thiochromen-4-one (**3d**)

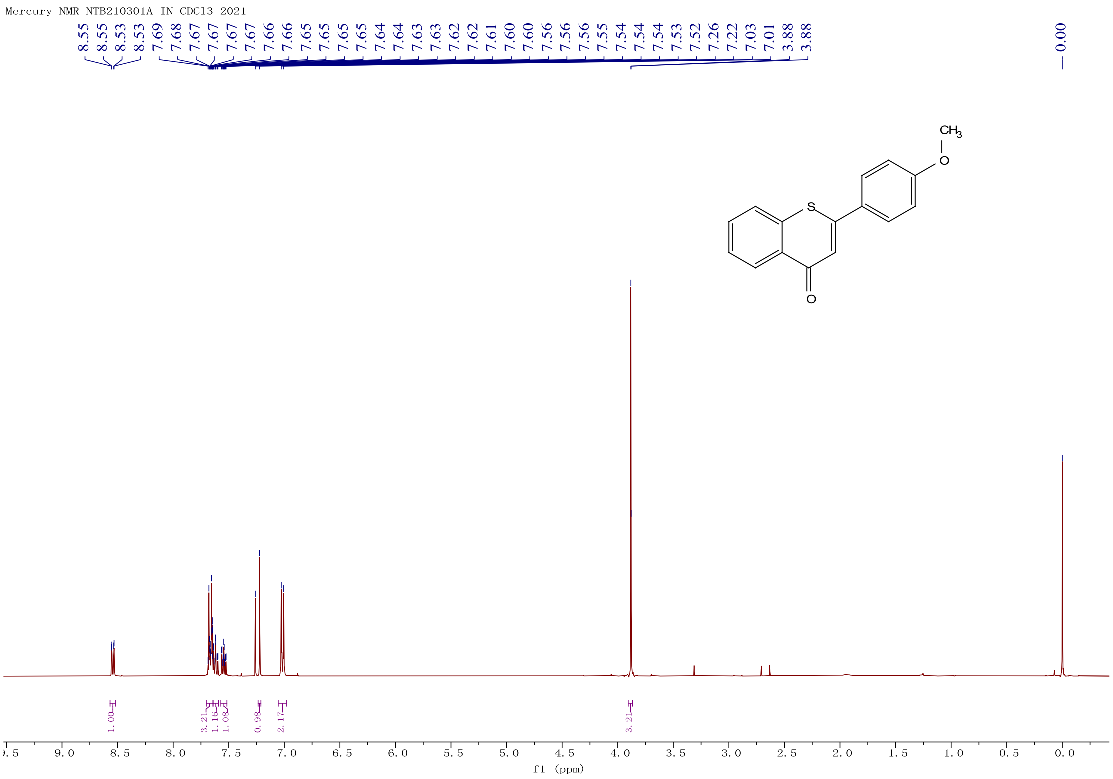

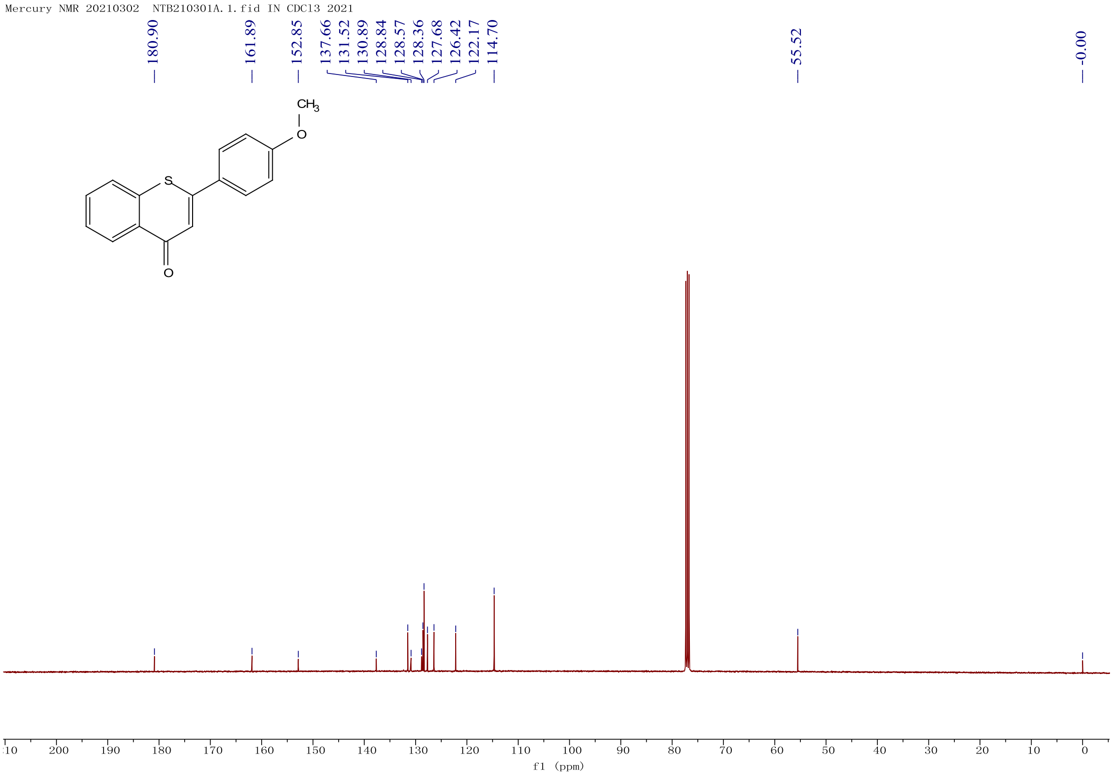

Figure S5. ^{1}H and ^{13}C NMR spectra of 2-(4-Methoxyphenyl)-4*H*-thiochromen-4-one (**3e**)

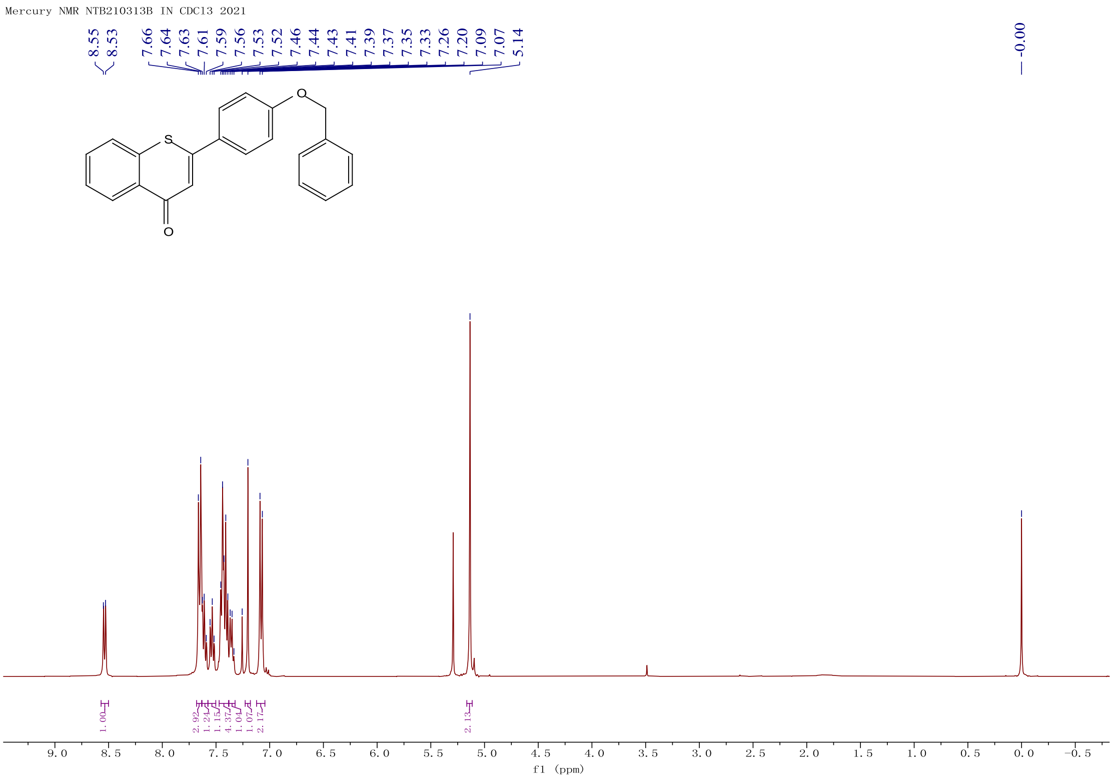

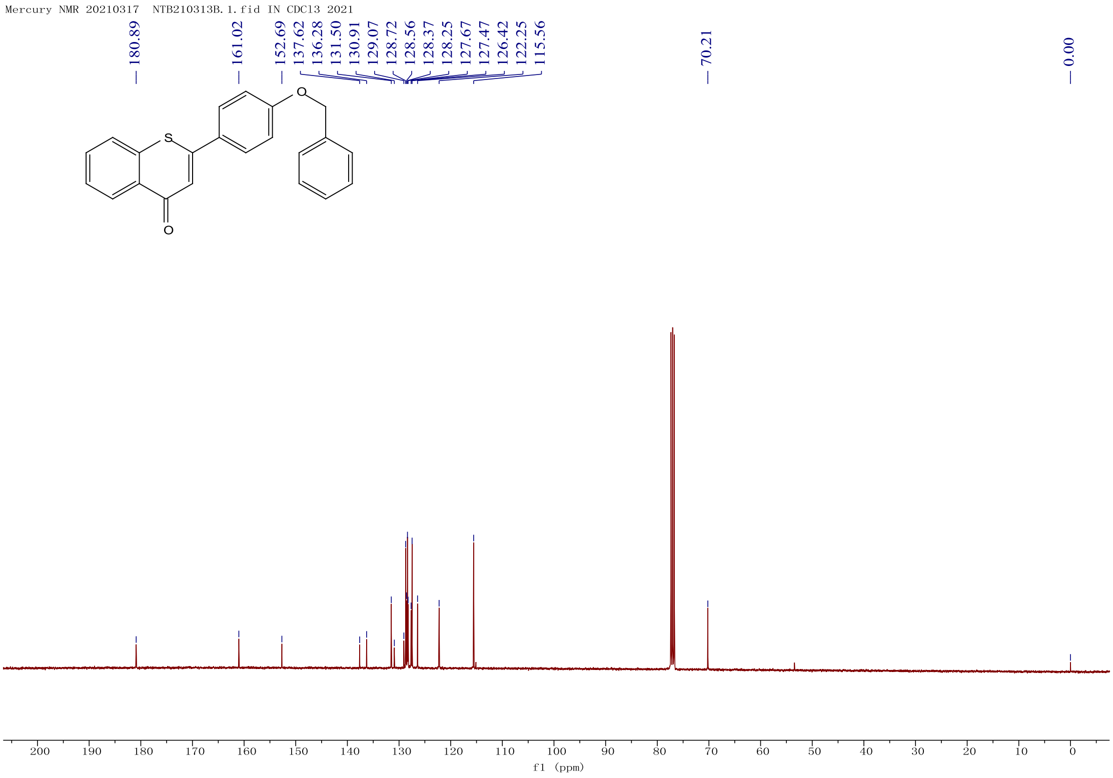

Figure S6. ^{1}H and ^{13}C NMR spectra of 2-(4-(Benzyloxy)phenyl)-4*H*-thiochromen-4-one (**3f**)

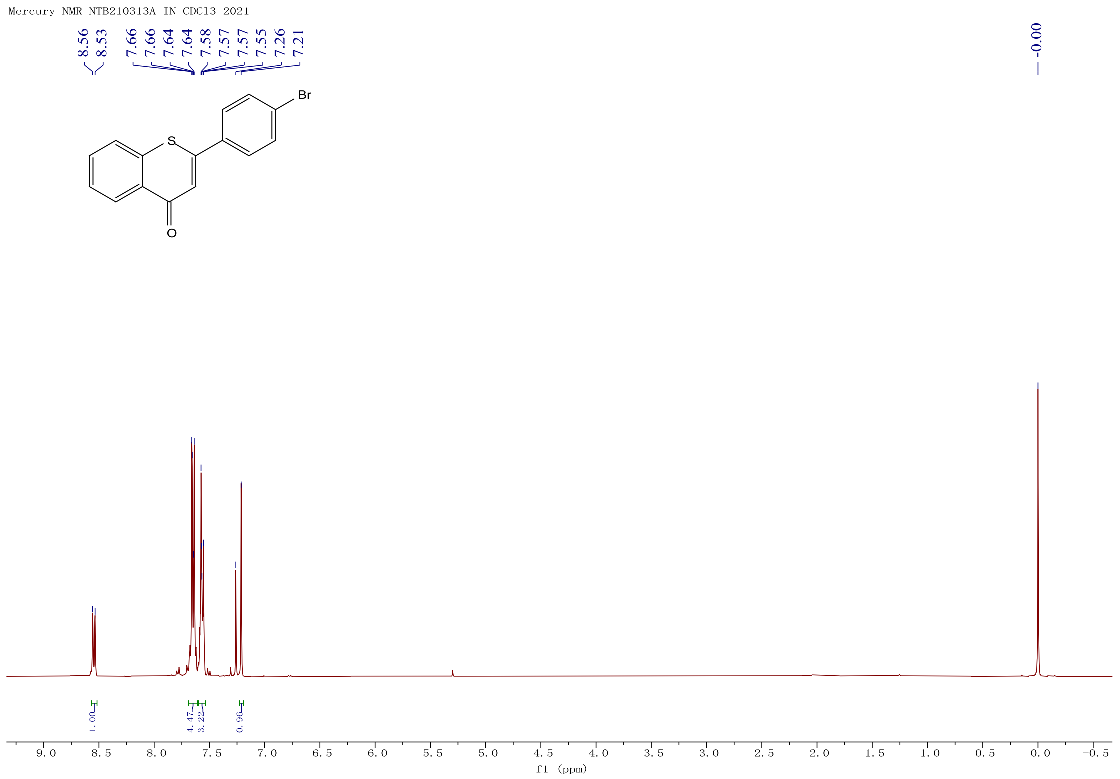

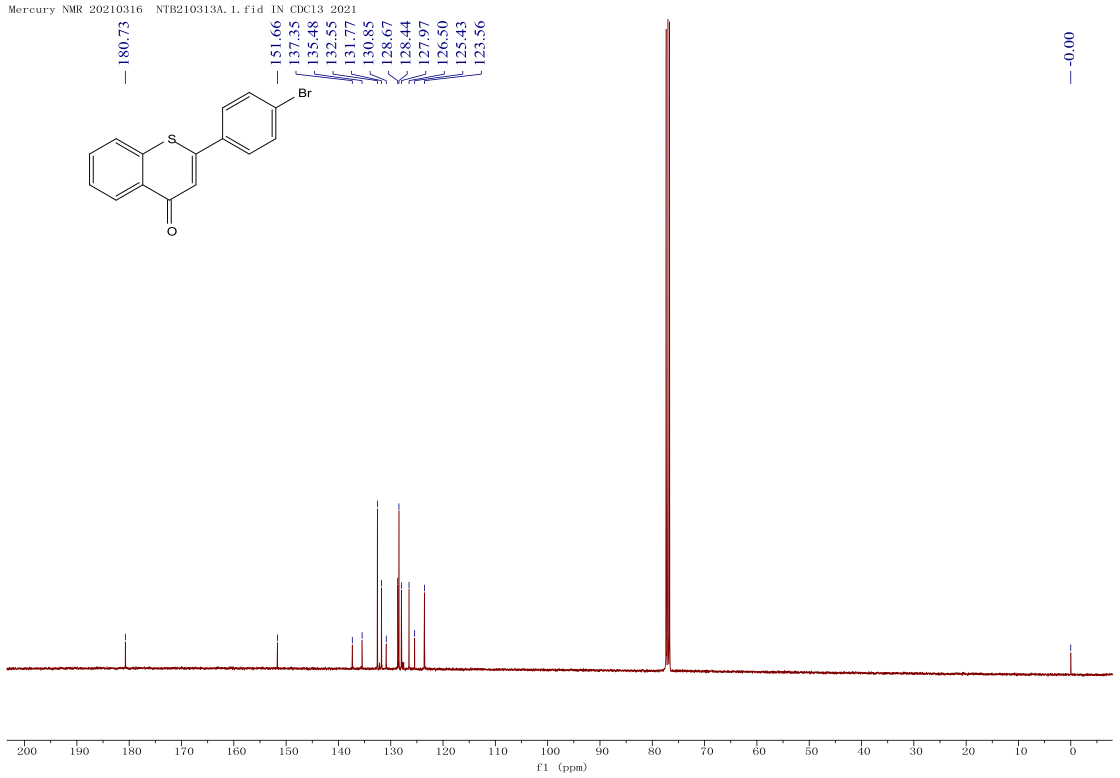

Figure S7. ^{1}H and ^{13}C NMR spectra of 2-(4-Bromophenyl)-4*H*-thiochromen-4-one (**3g**)

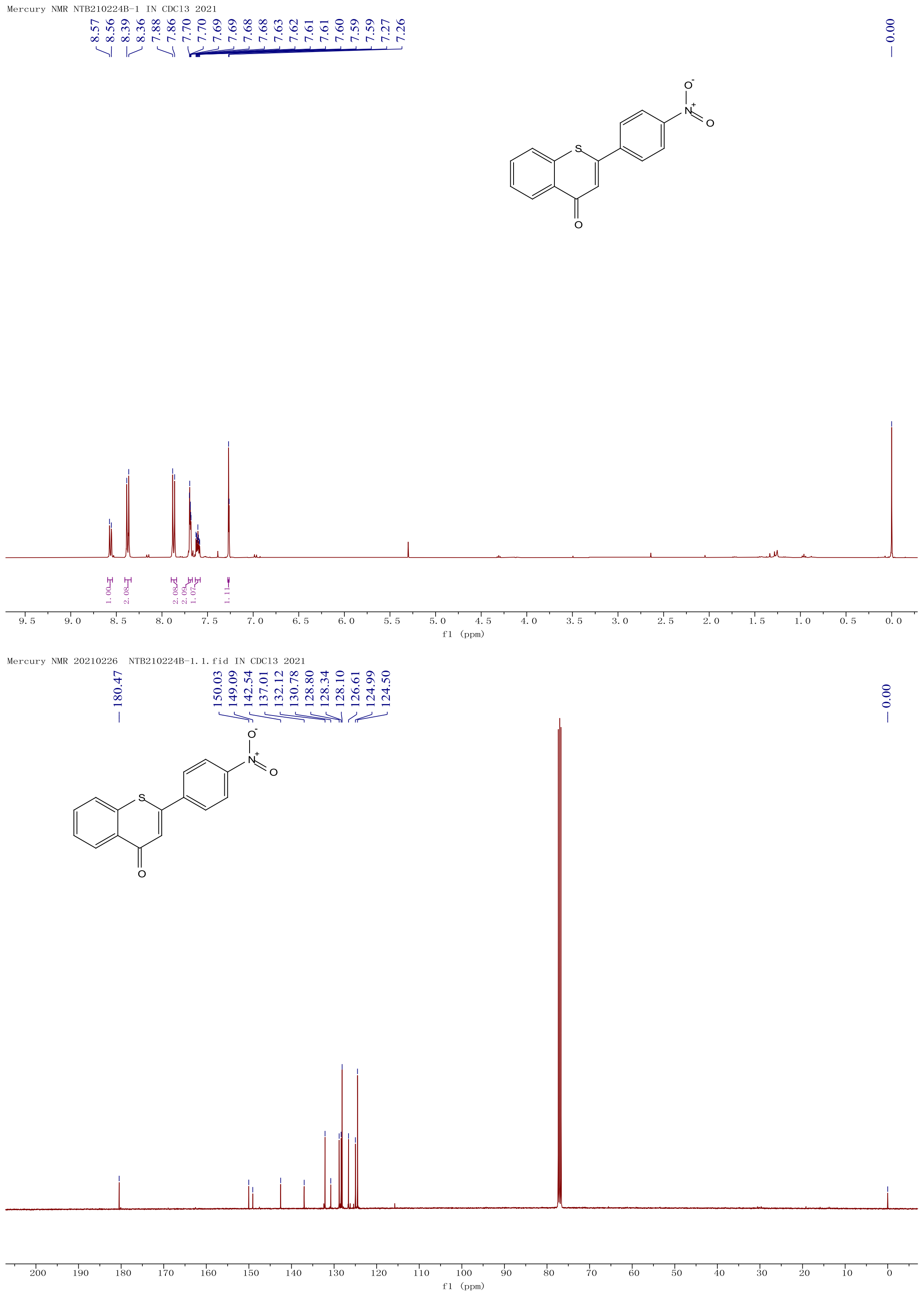

Figure S8. ^{1}H and ^{13}C NMR spectra of 2-(4-Nitrophenyl)-4*H*-thiochromen-4-one (**3h**)

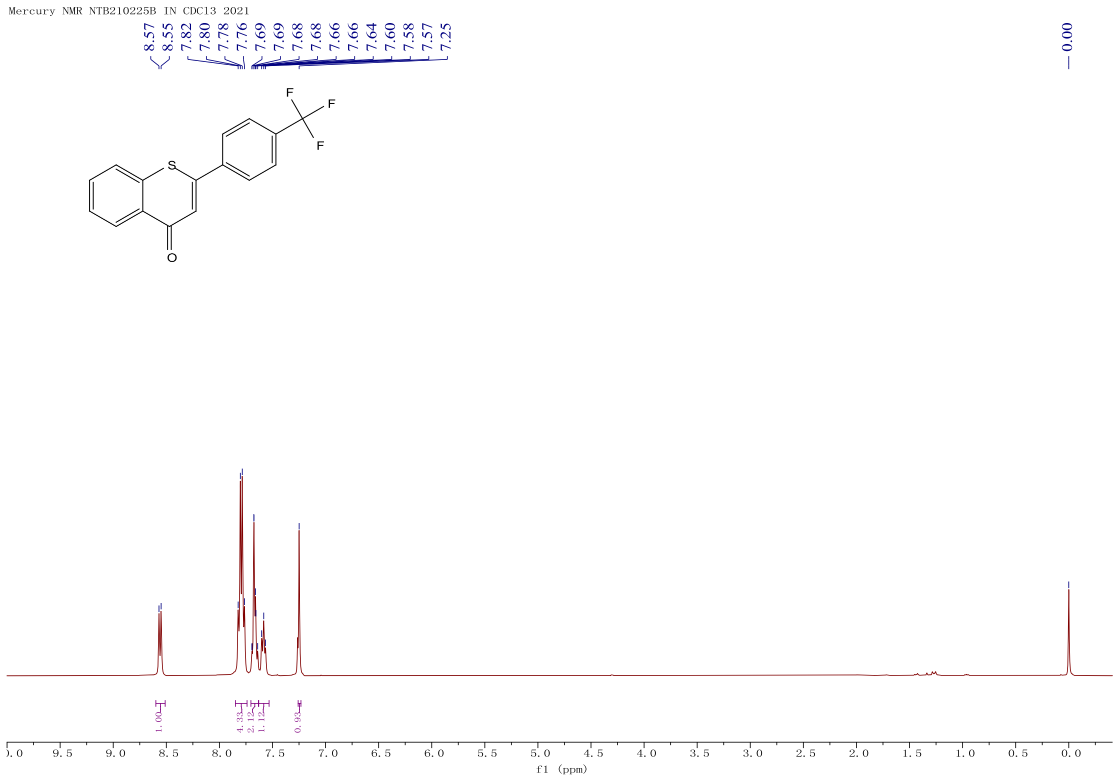

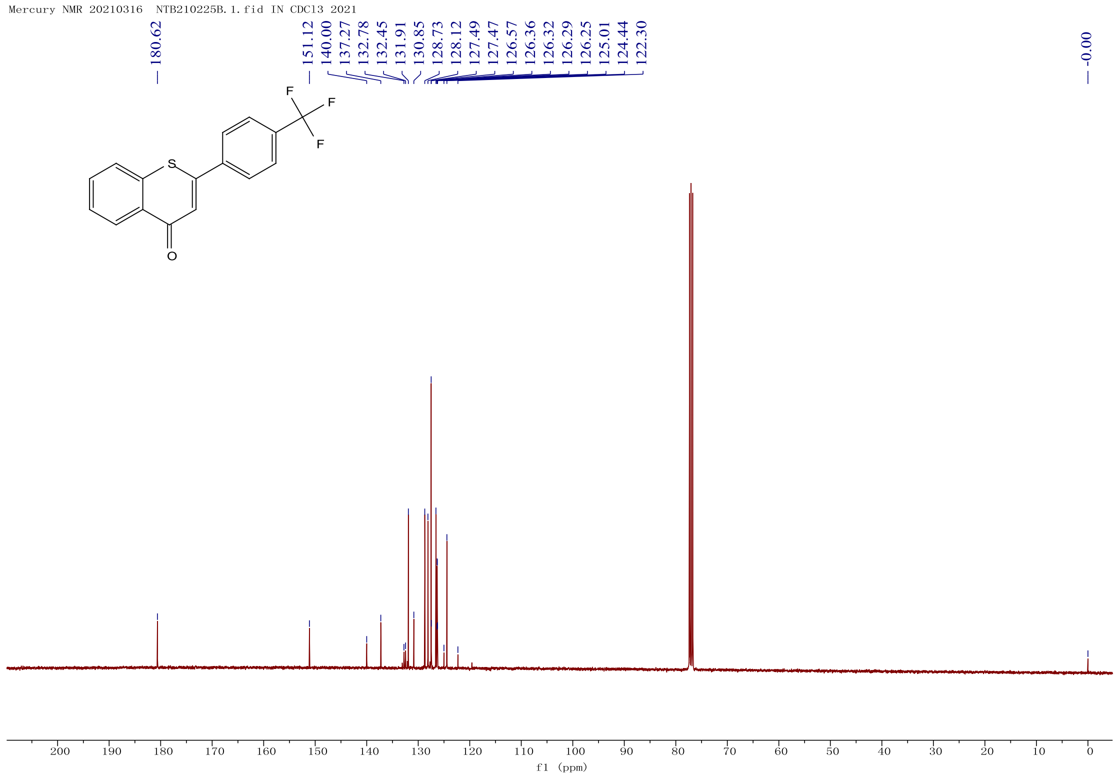

Figure S9. ^{1}H and ^{13}C NMR spectra of 2-(4-(Trifluoromethyl)phenyl)-4*H*-thiochromen-4-one (**3i**)

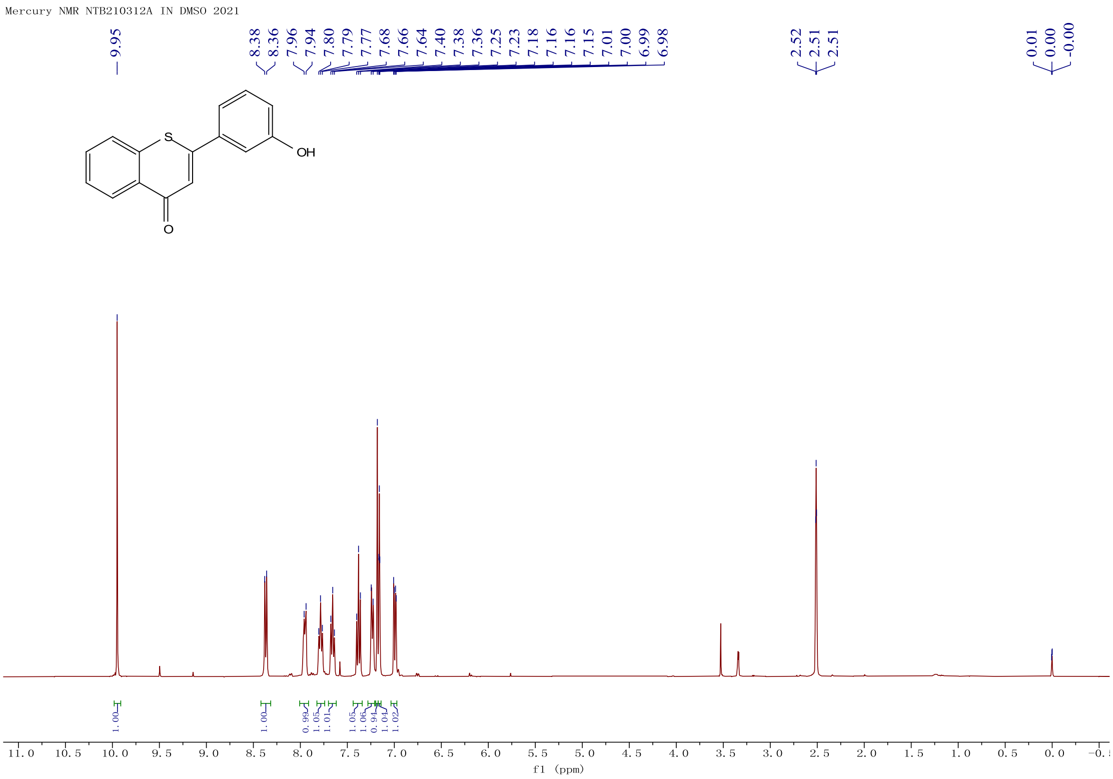

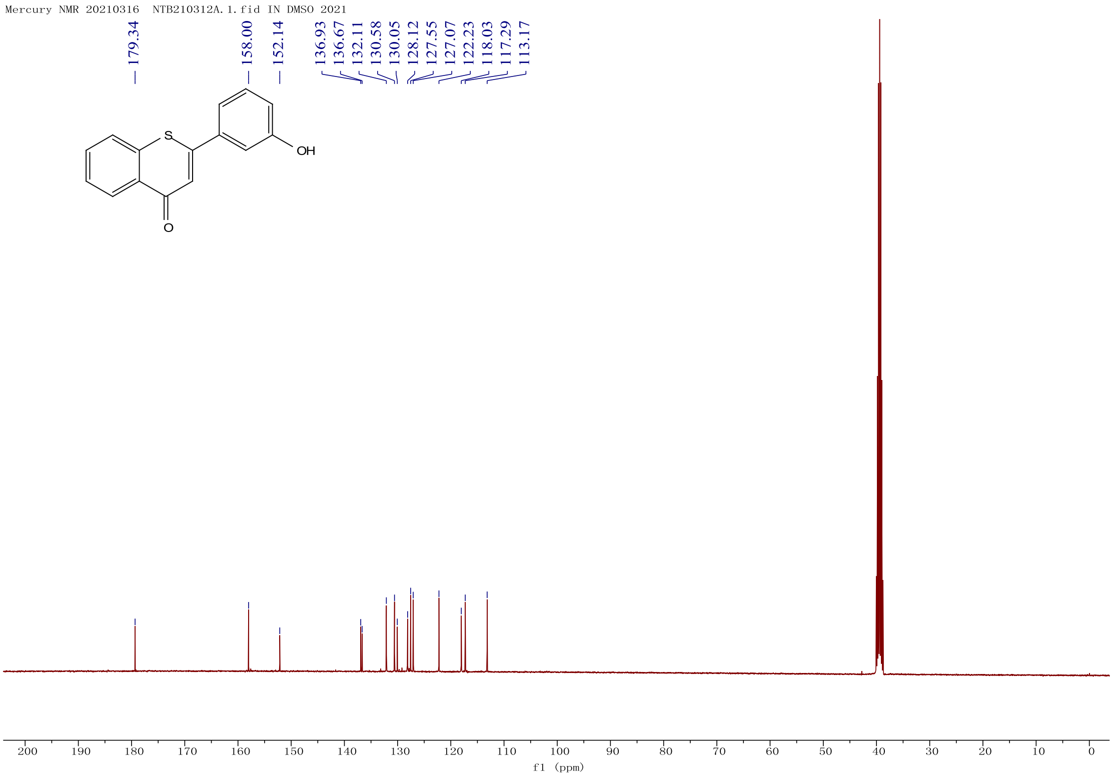

Figure S10. ^{1}H and ^{13}C NMR spectra of 2-(3-Hydroxyphenyl)-4*H*-thiochromen-4-one (**3j**)

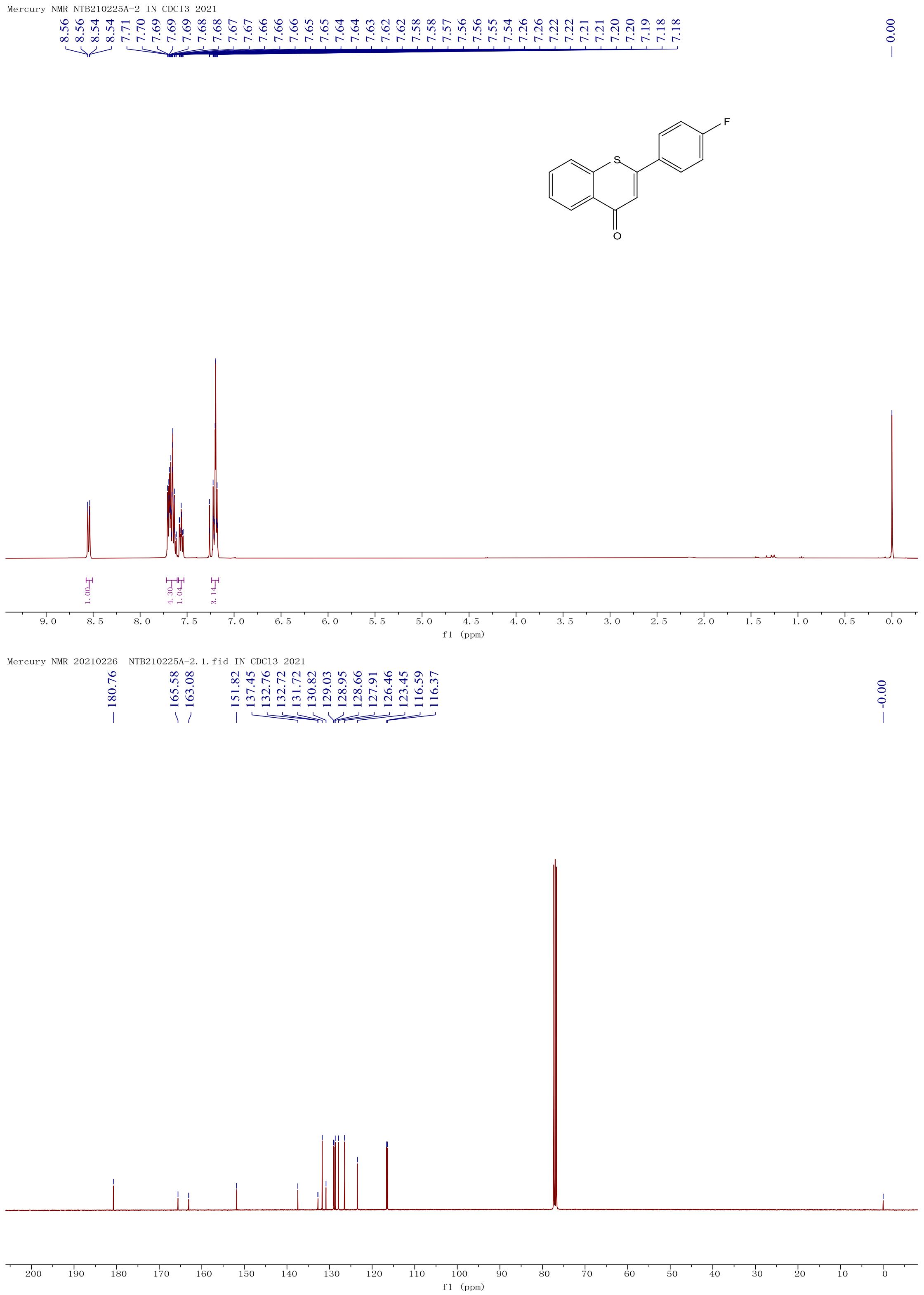

Figure S11. ^{1}H and ^{13}C NMR spectra of 2-(4-Fluorophenyl)-4*H*-thiochromen-4-one (**3k**)

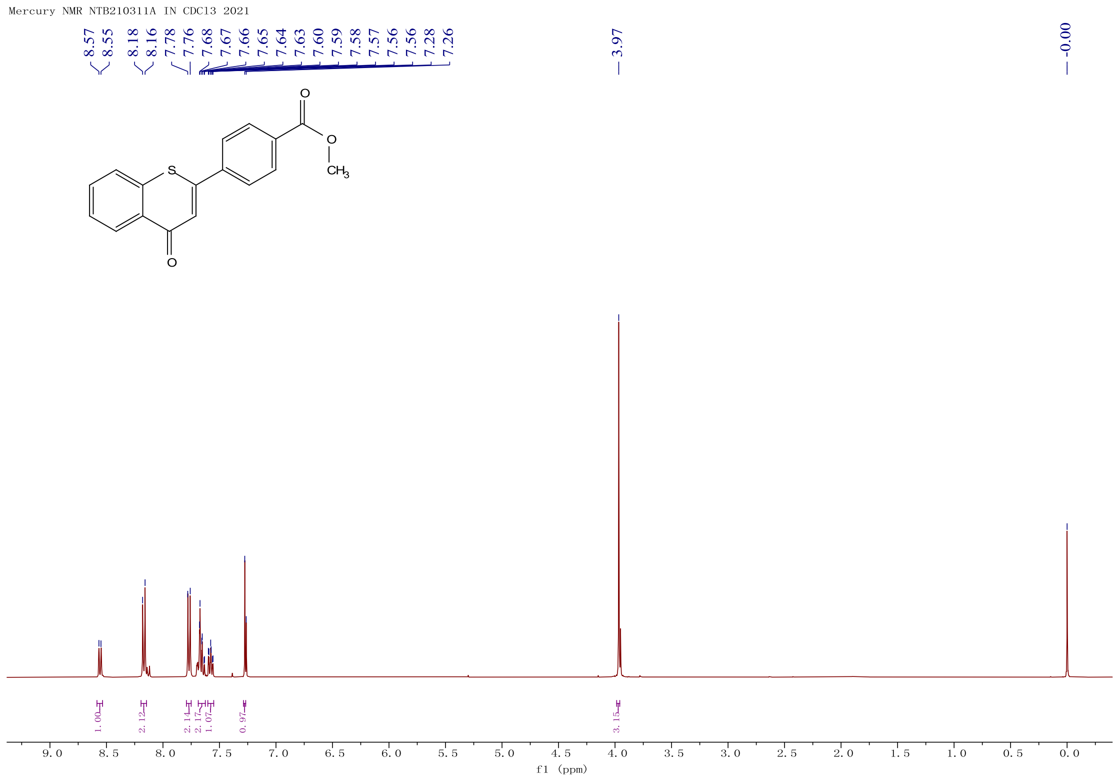

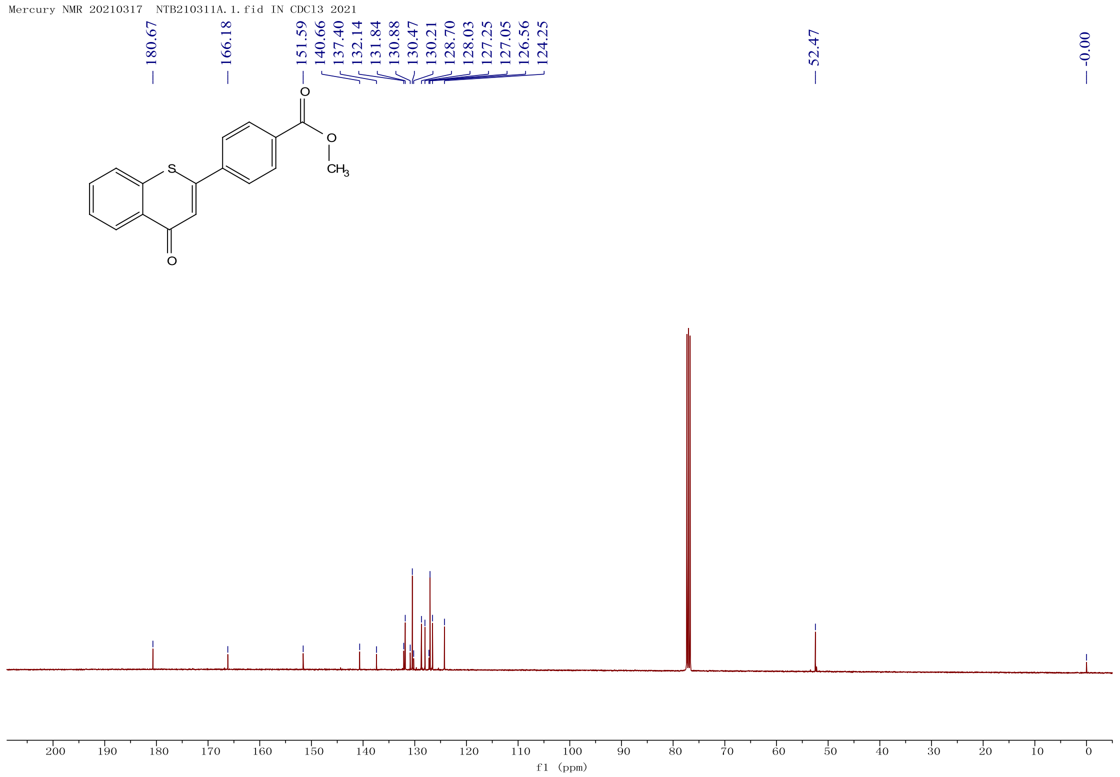

Figure S12. ^{1}H and ^{13}C NMR spectra of 2-(4-Methoxycarbonylphenyl)-4*H*-thiochromen-4-one (**3l**)

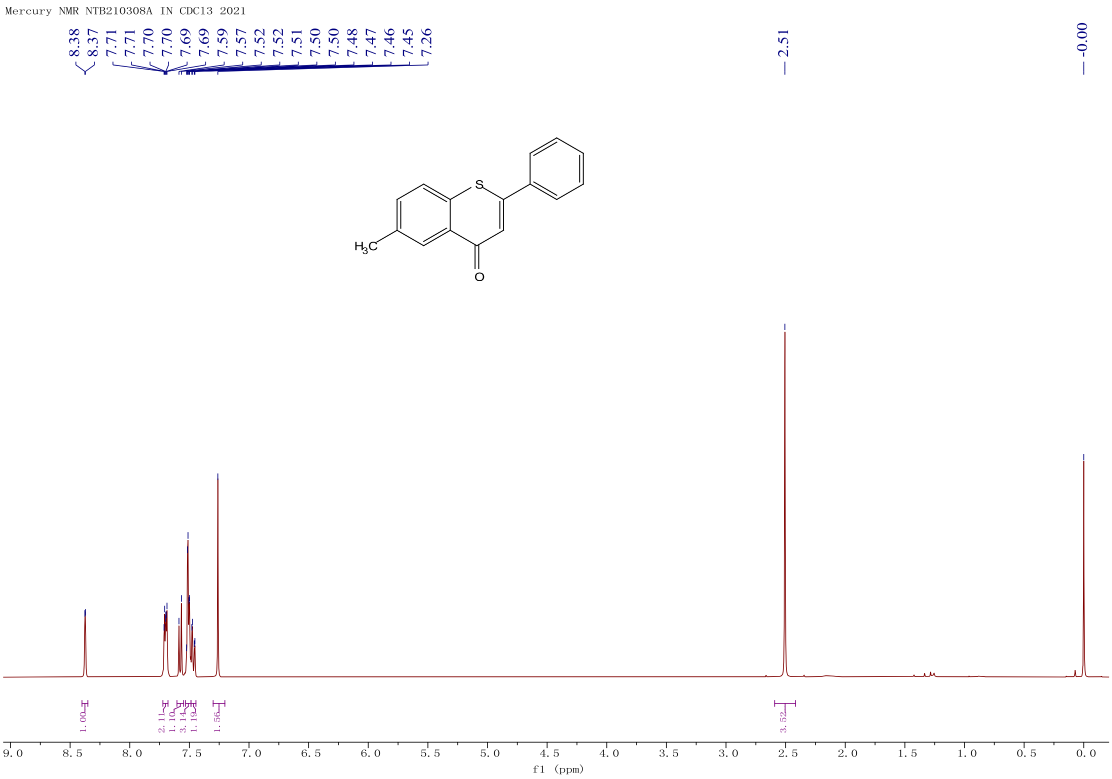

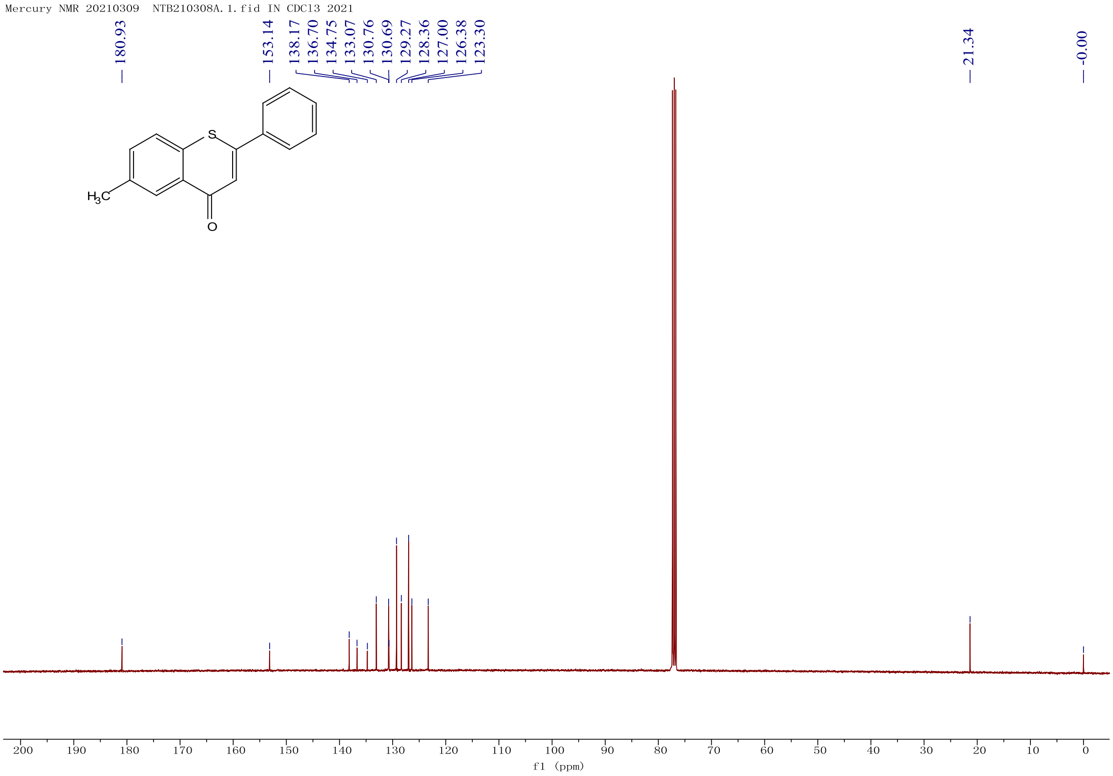

Figure S13. ^{1}H and ^{13}C NMR spectra of 6-Methyl-2-phenyl-4*H*-thiochromen-4-one (**3m**)

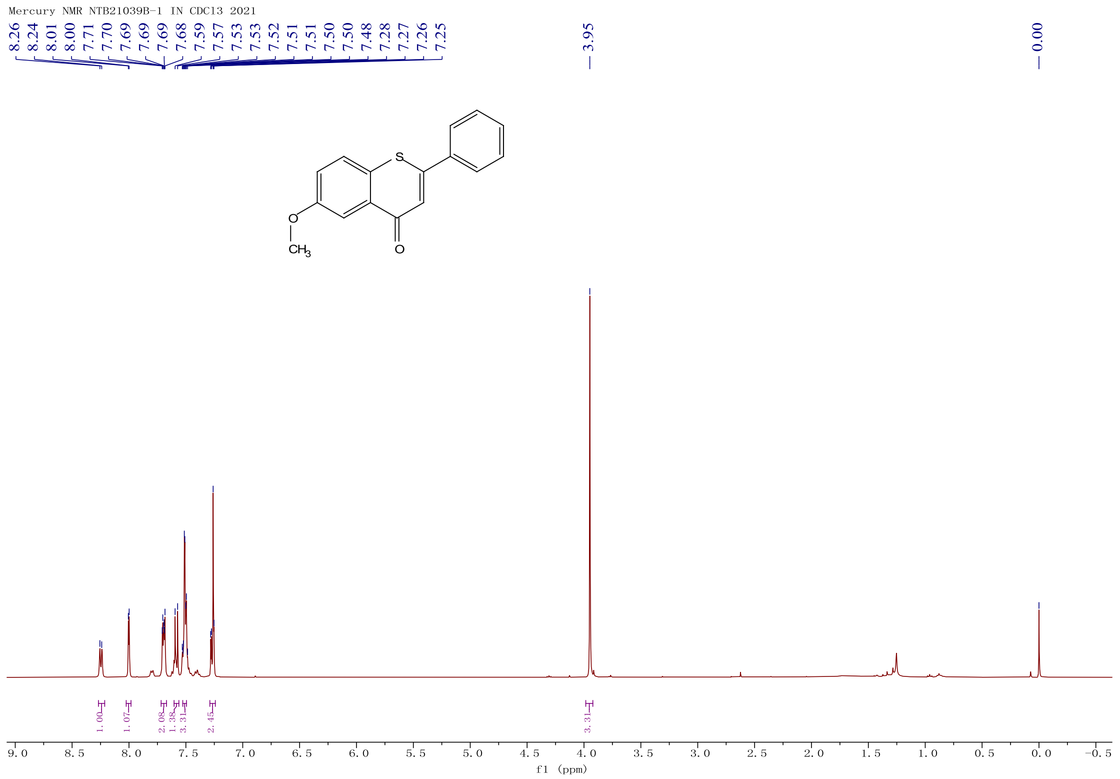

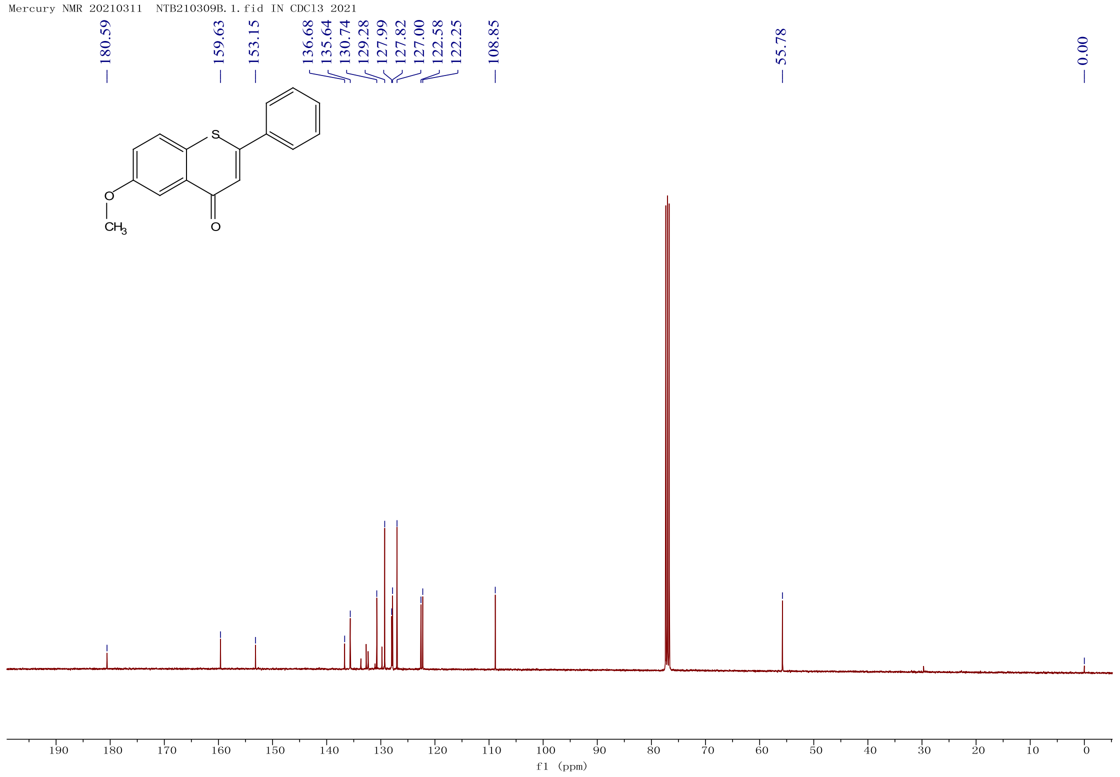

Figure S14. ^{1}H and ^{13}C NMR spectra of 6-Methoxy-2-phenyl-4*H*-thiochromen-4-one (**3n**)

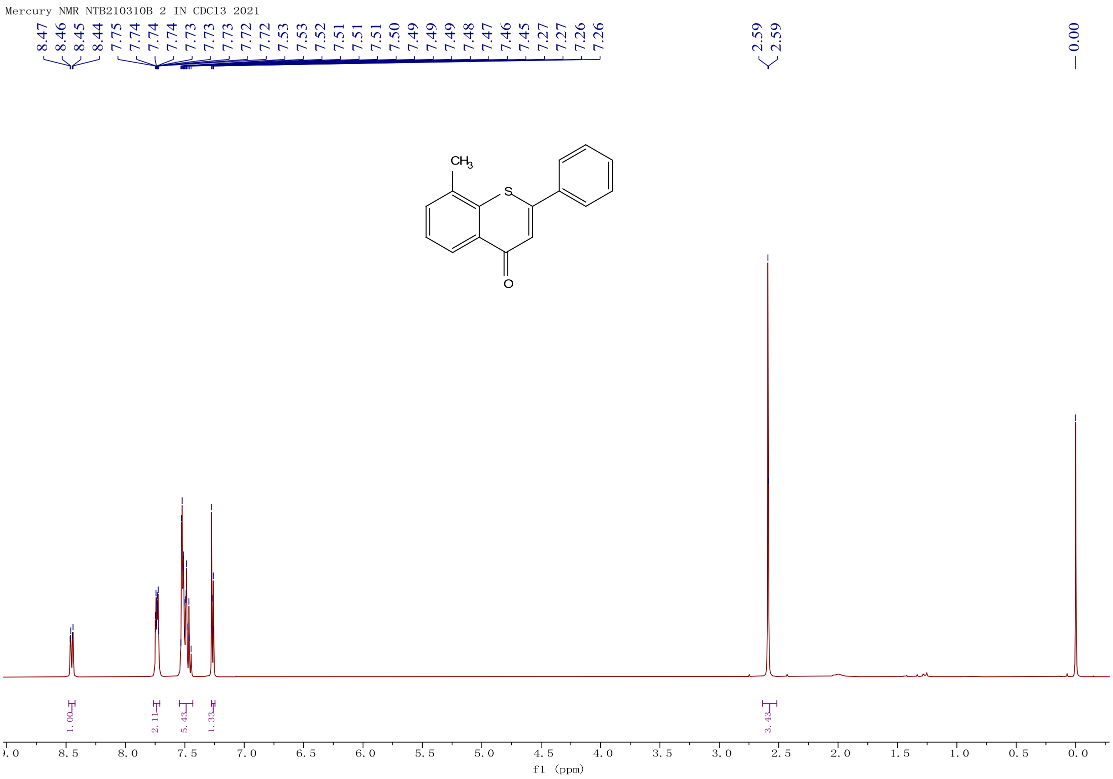

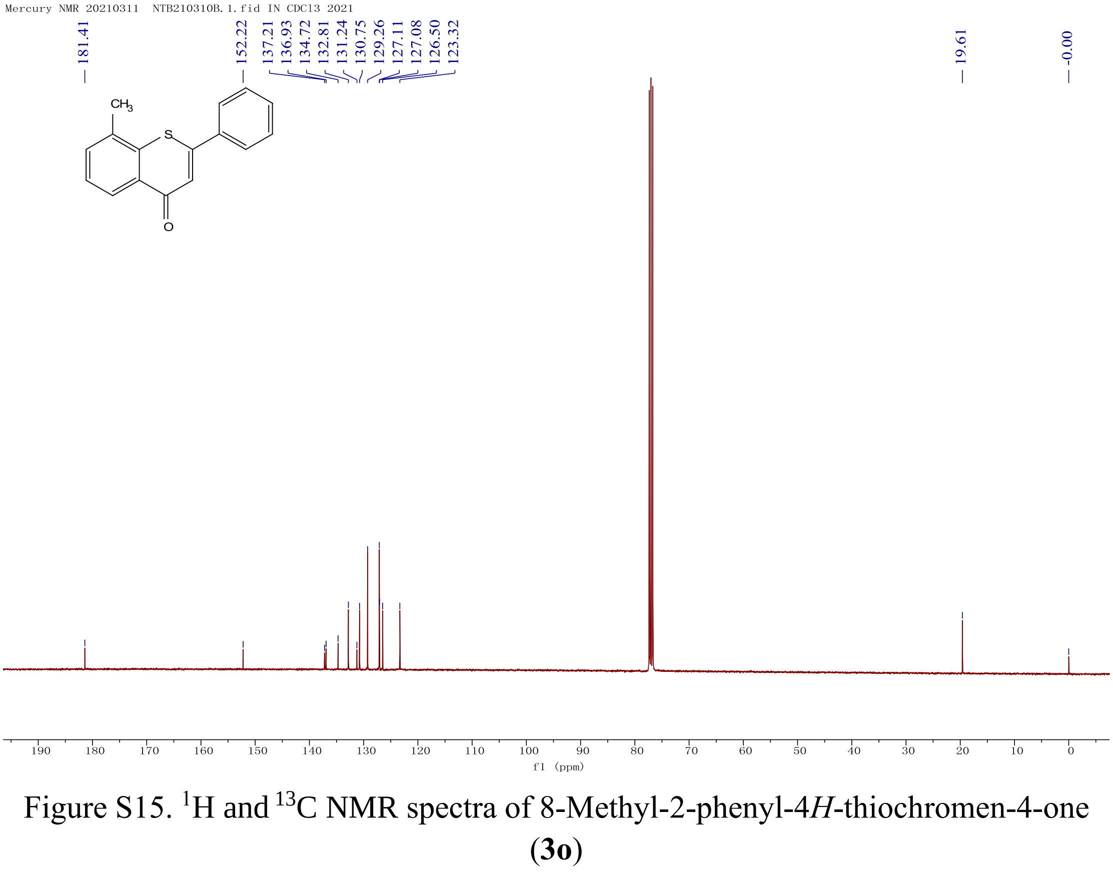

Figure S15. ^{1}H and ^{13}C NMR spectra of 8-Methyl-2-phenyl-4*H*-thiochromen-4-one (**3o**)

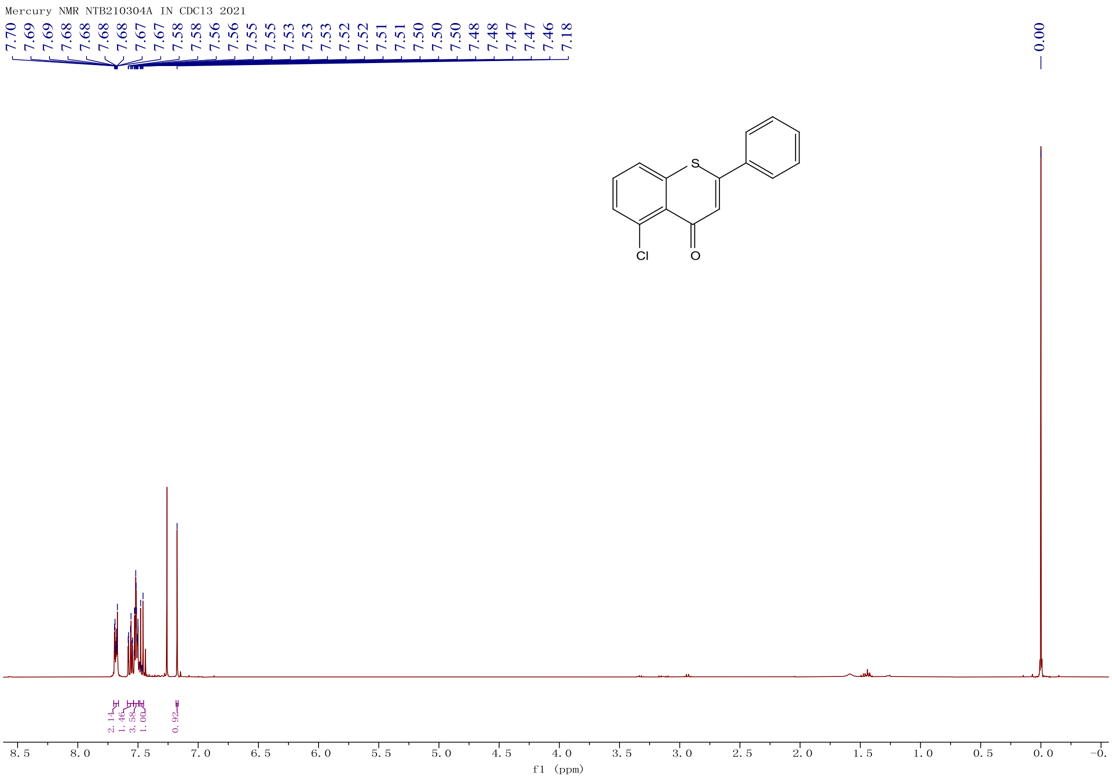

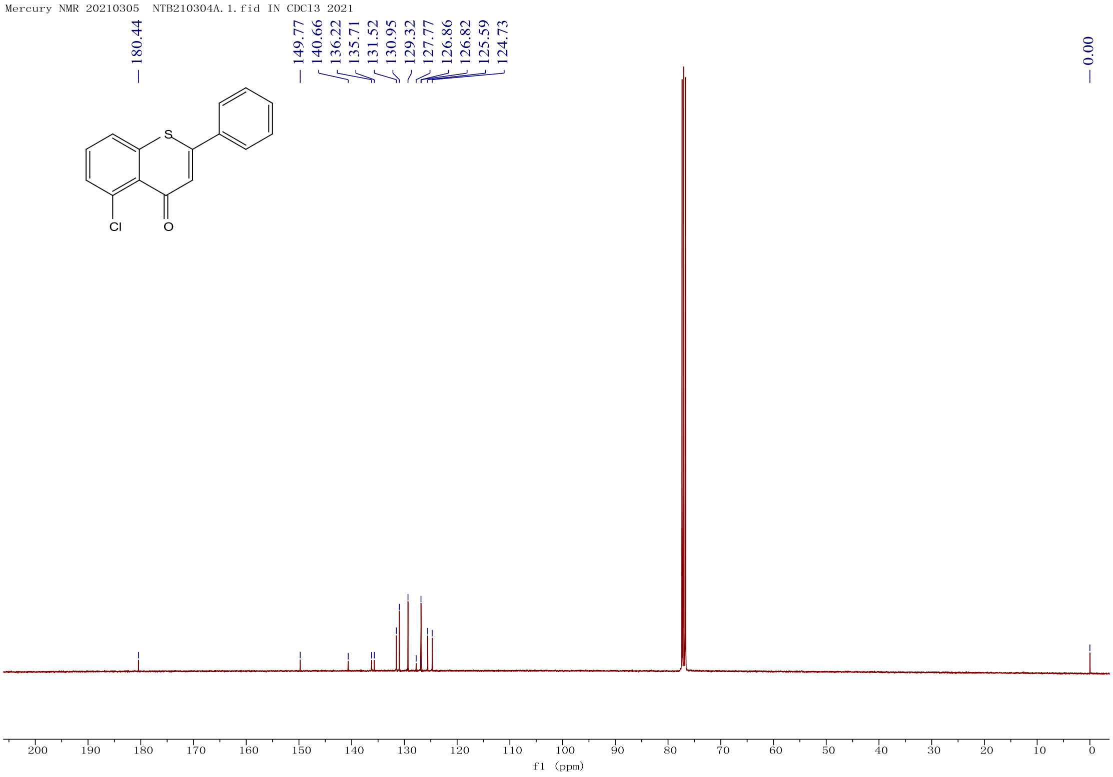

Figure S16. ^{1}H and ^{13}C NMR spectra of 5-Chloro-2-phenyl-4*H*-thiochromen-4-one (**3p**)

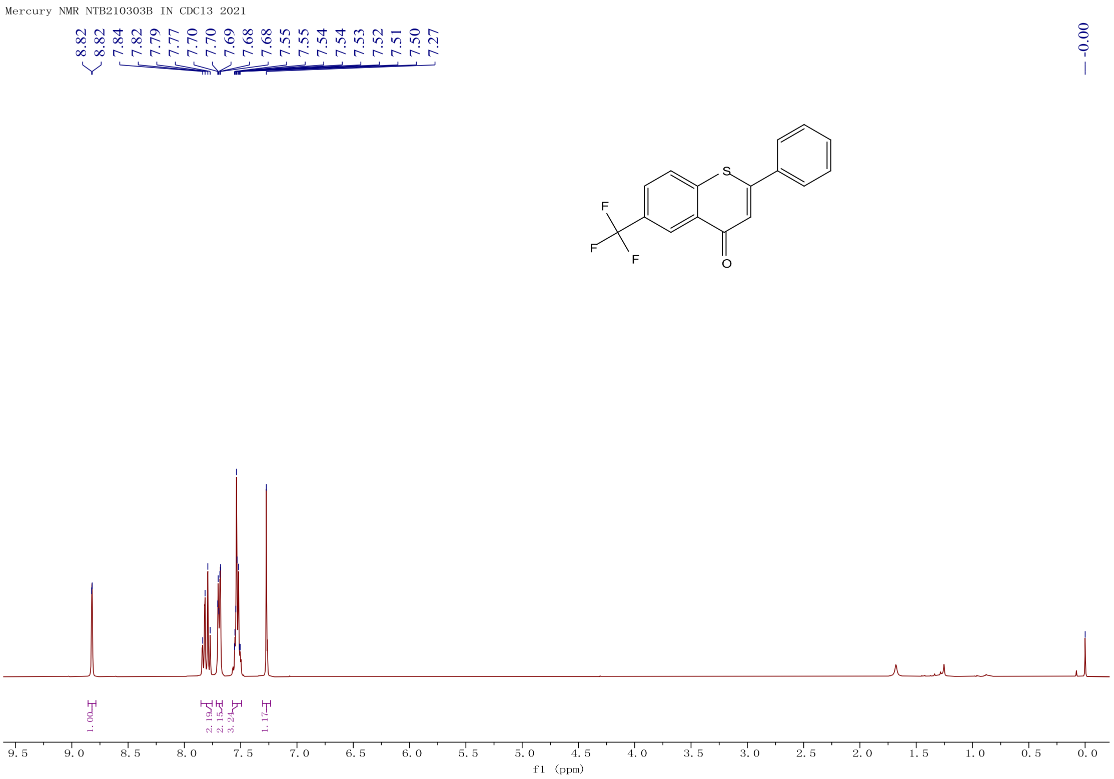

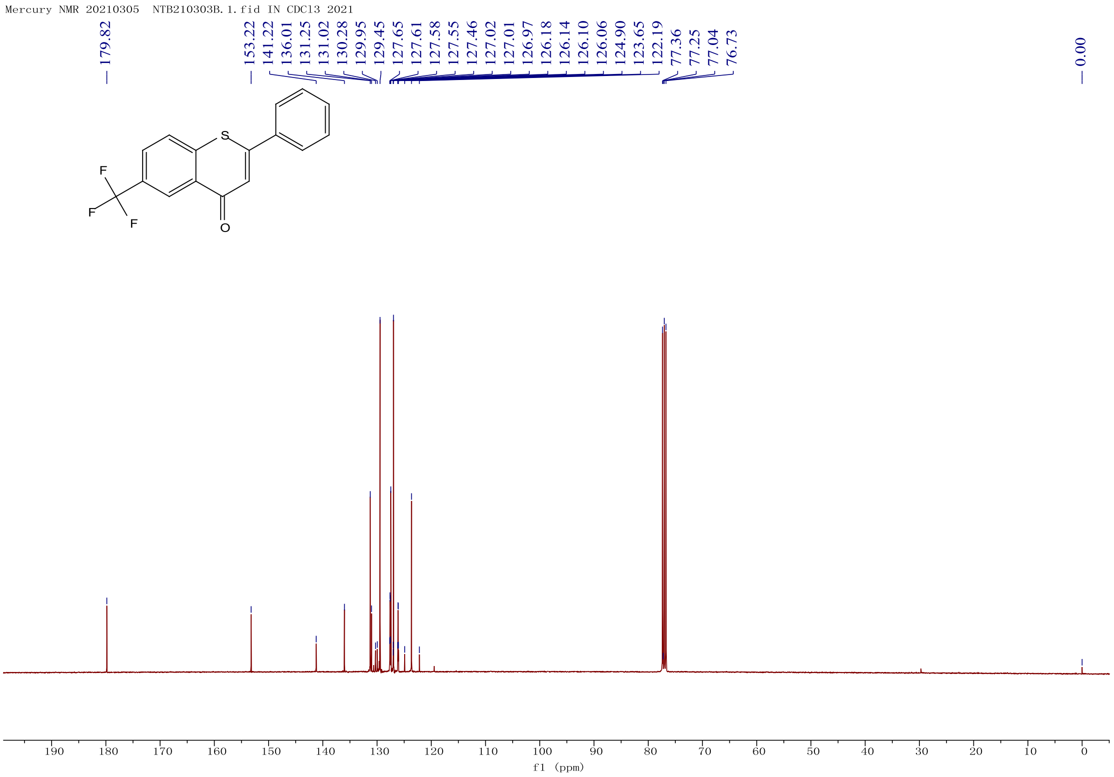

Figure S17. ^{1}H and ^{13}C NMR spectra of 2-Phenyl-6-(trifluoromethyl)-4*H*-thiochromen-4-one (**3q**)

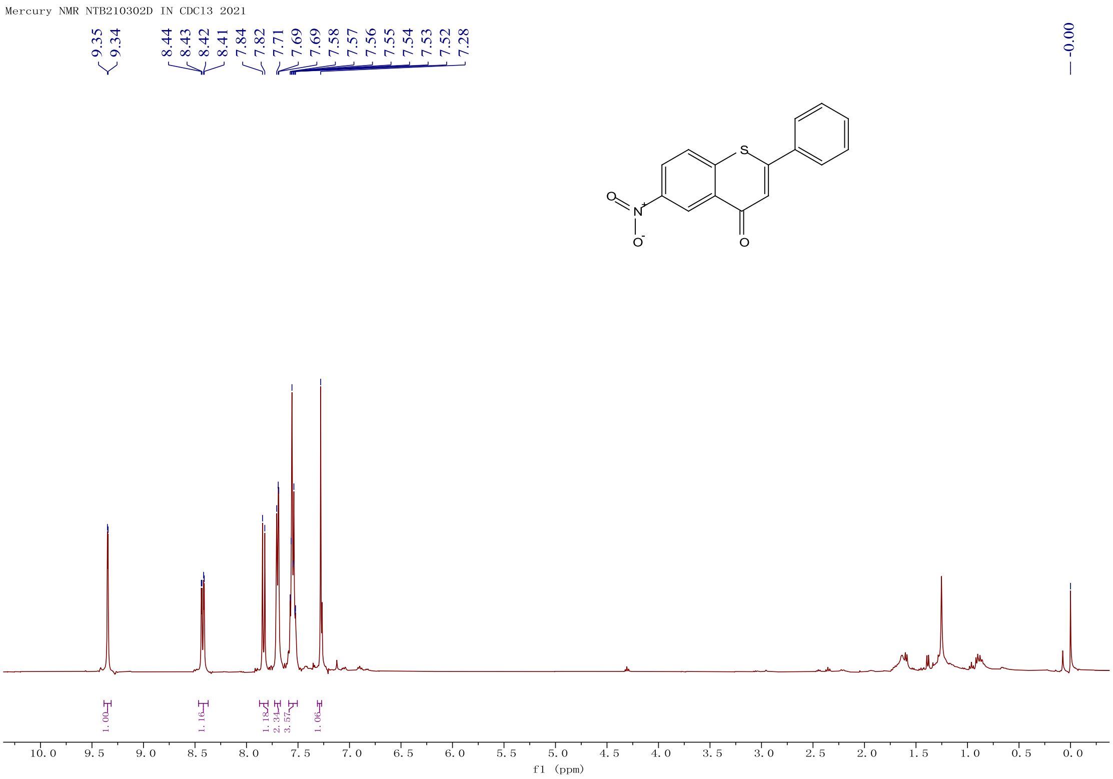

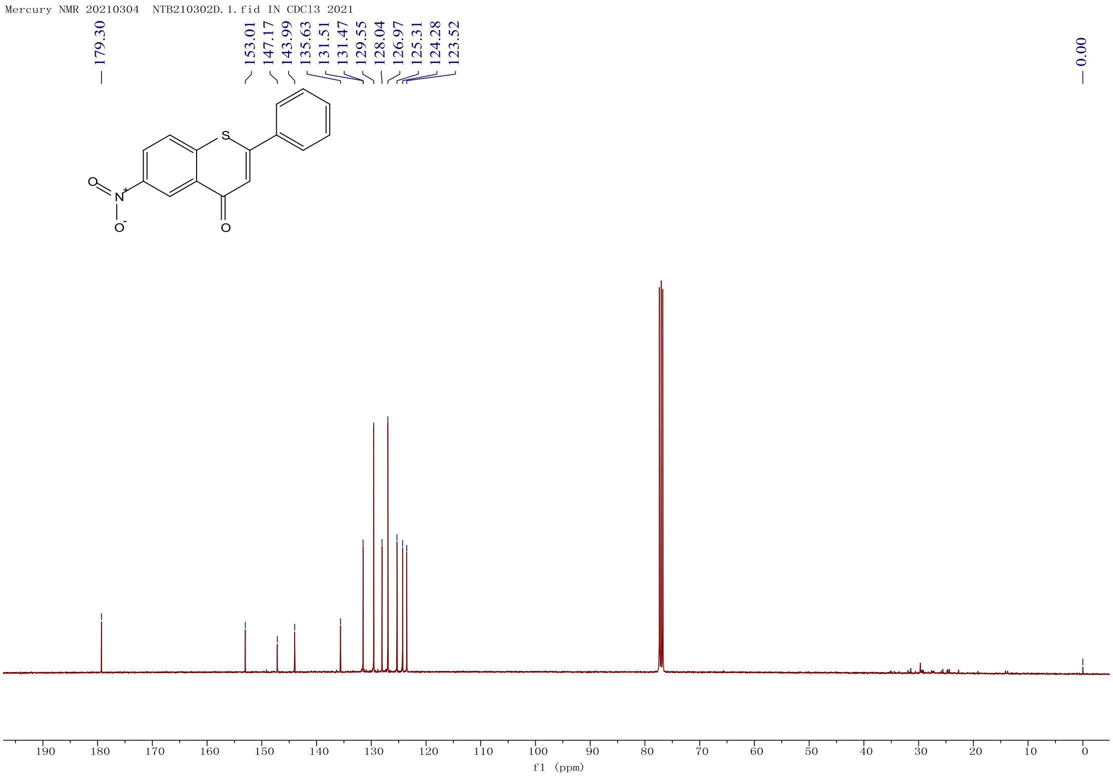

Figure S18. ^{1}H and ^{13}C NMR spectra of 6-Nitro-2-phenyl-4*H*-thiochromen-4-one (**3r**)

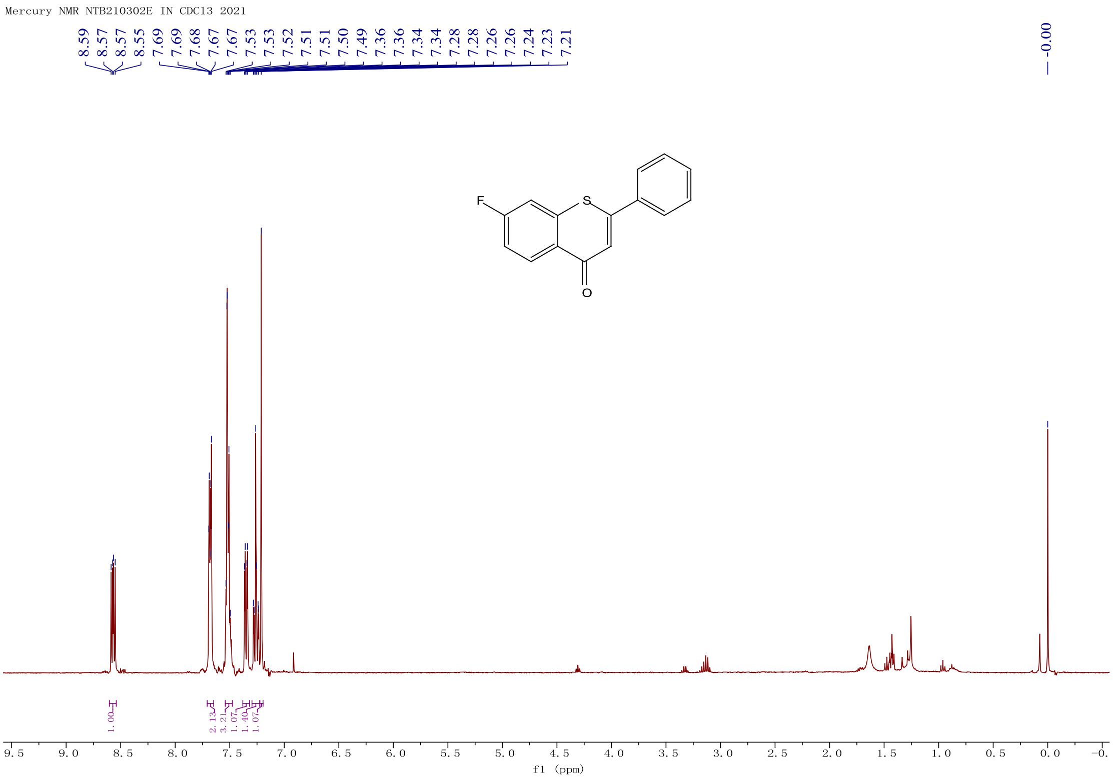

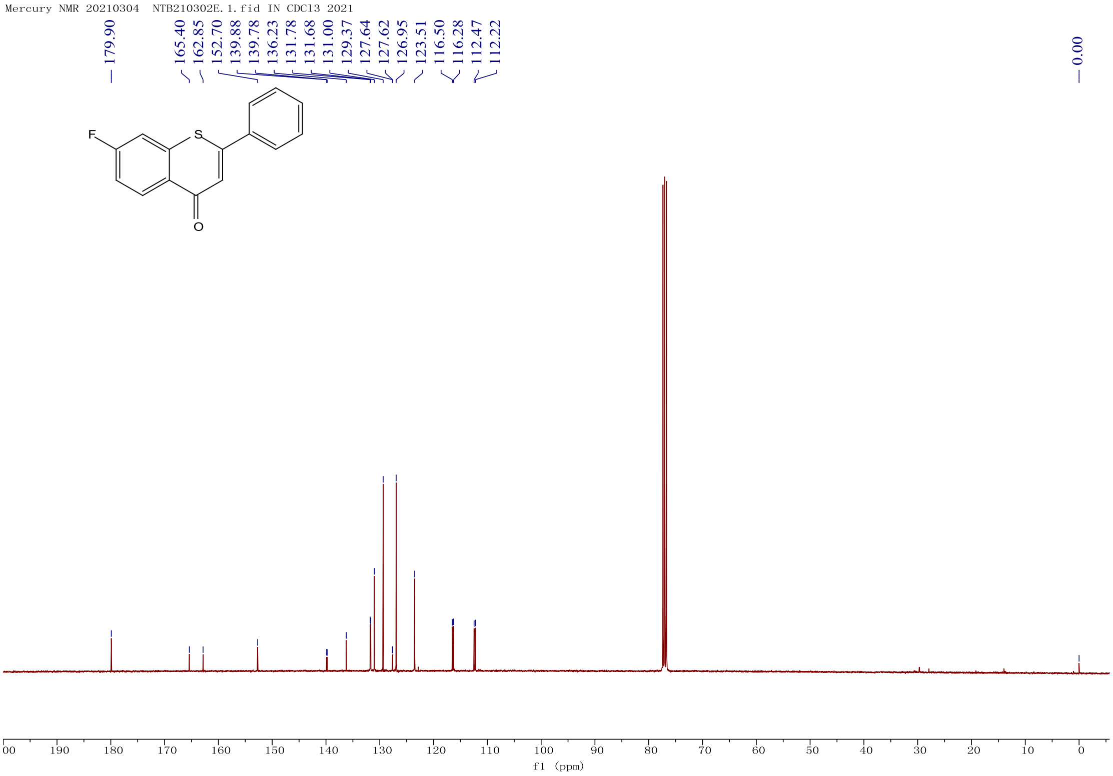

Figure S19. ^{1}H and ^{13}C NMR spectra of 7-Fluoro-2-phenyl-4*H*-thiochromen-4-one (**3s**)

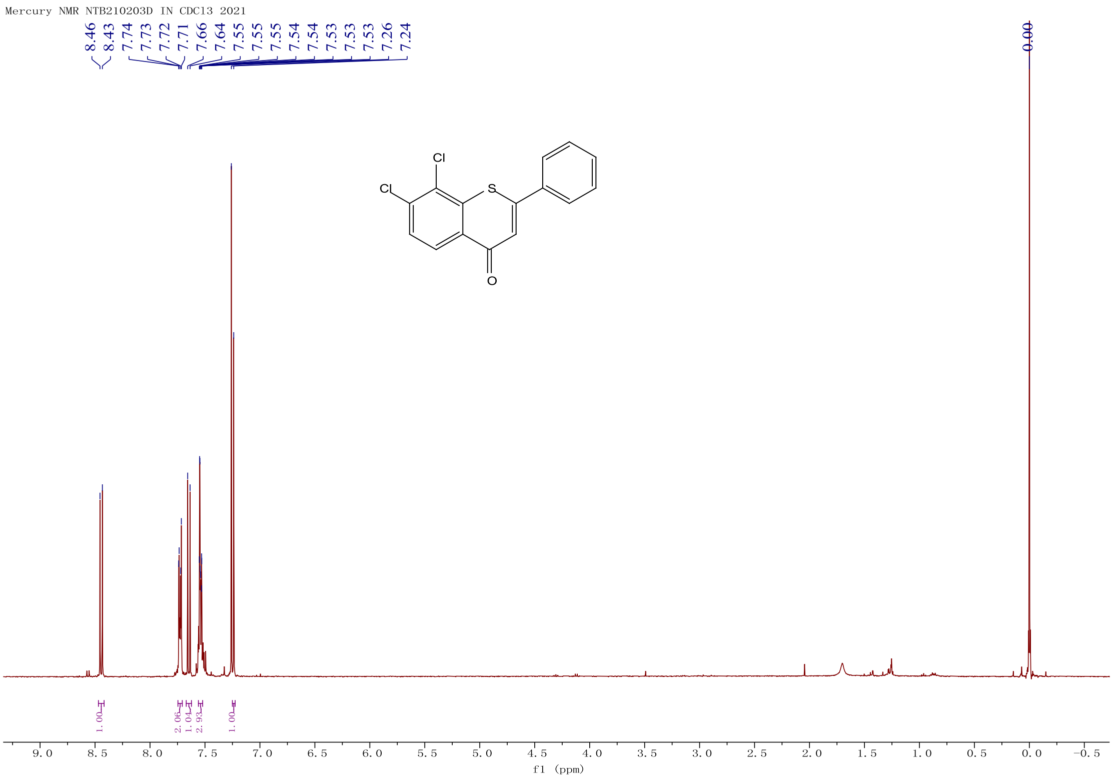

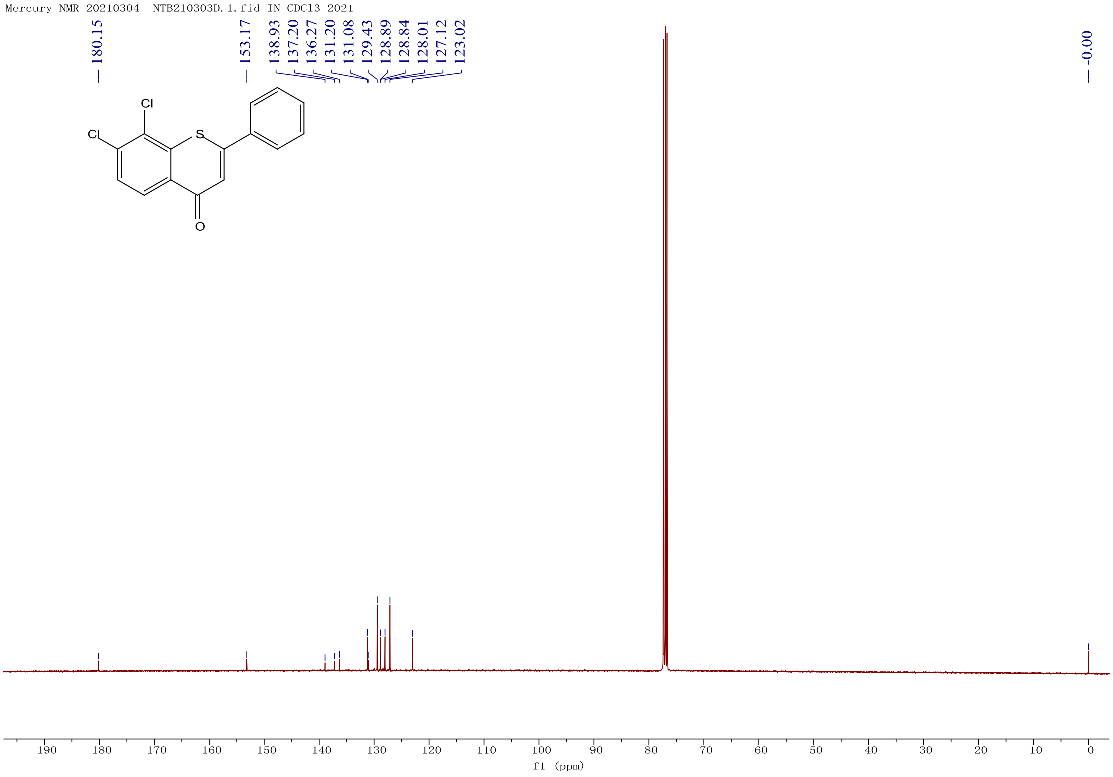

Figure S20. ^{1}H and ^{13}C NMR spectra of 7,8-Dichloro-2-phenyl-4*H*-thiochromen-4-one (**3t**)

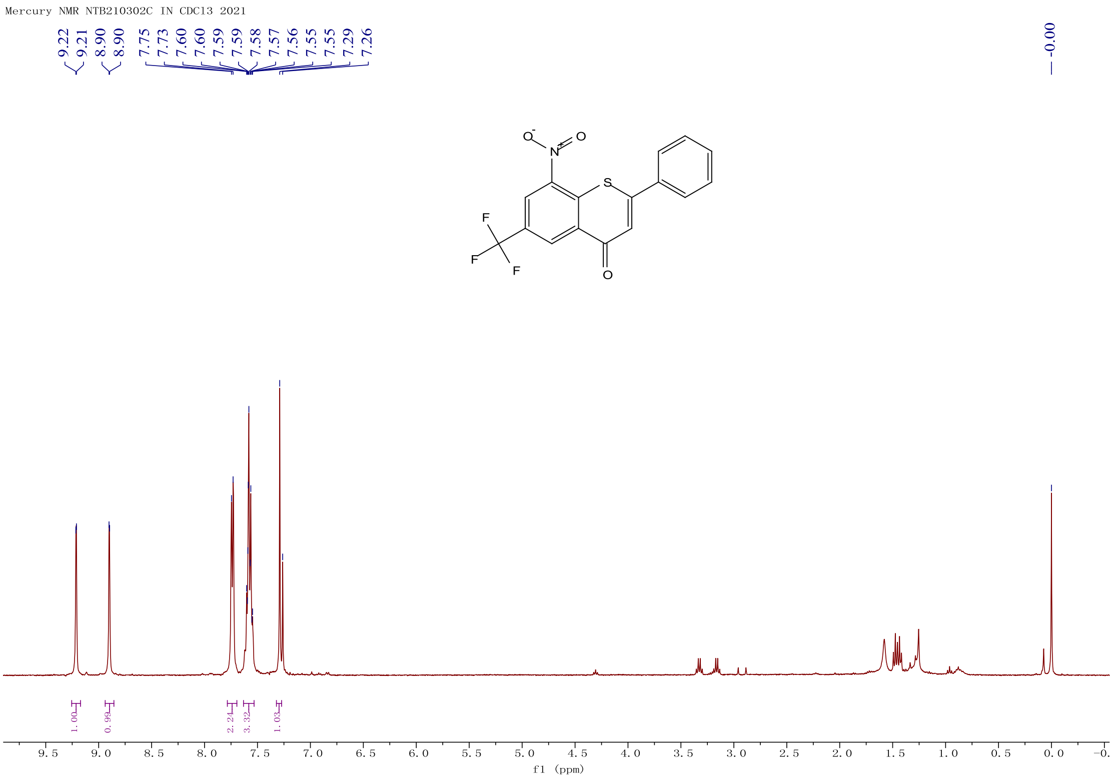

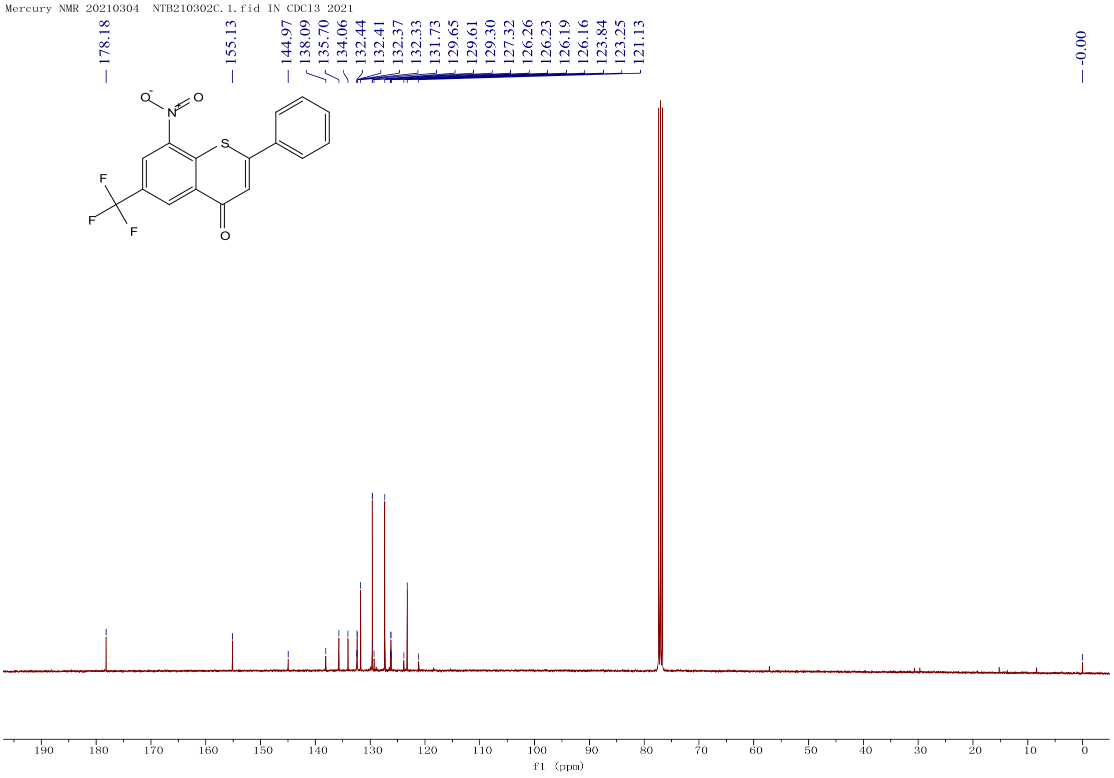

Figure S21. ^{1}H and ^{13}C NMR spectra of 8-Nitro-2-phenyl-6-(trifluoromethyl)-4*H*-thiochromen-4-one (**3u**)

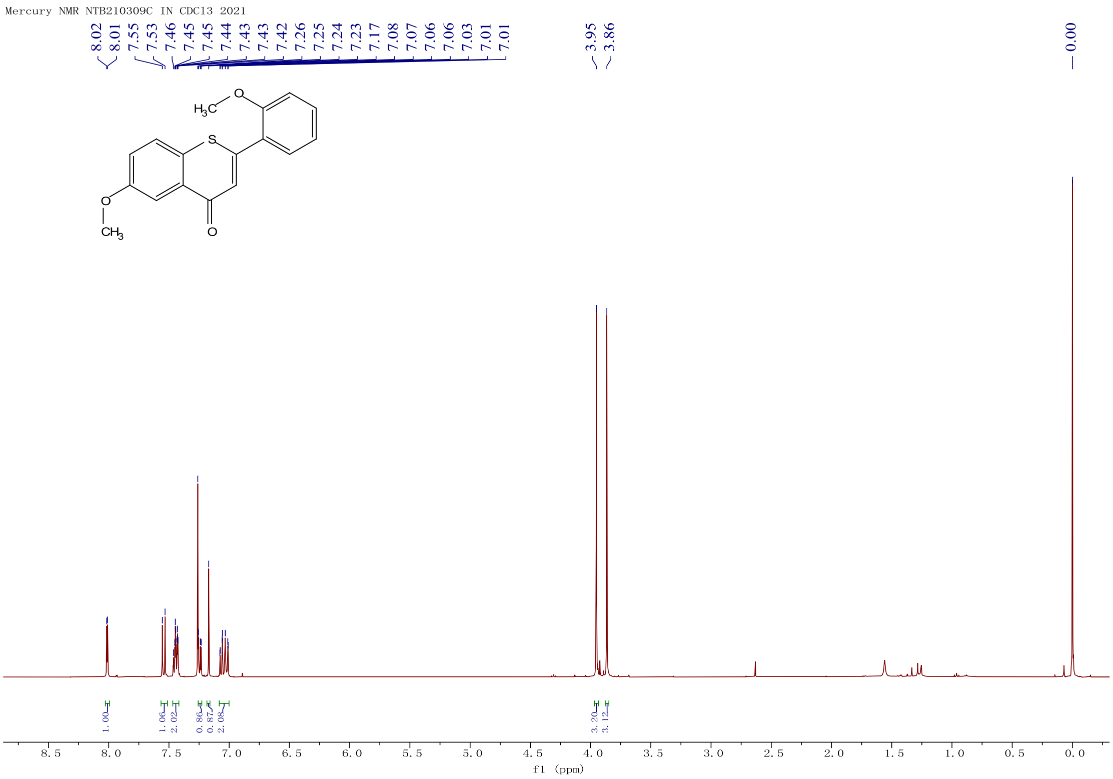

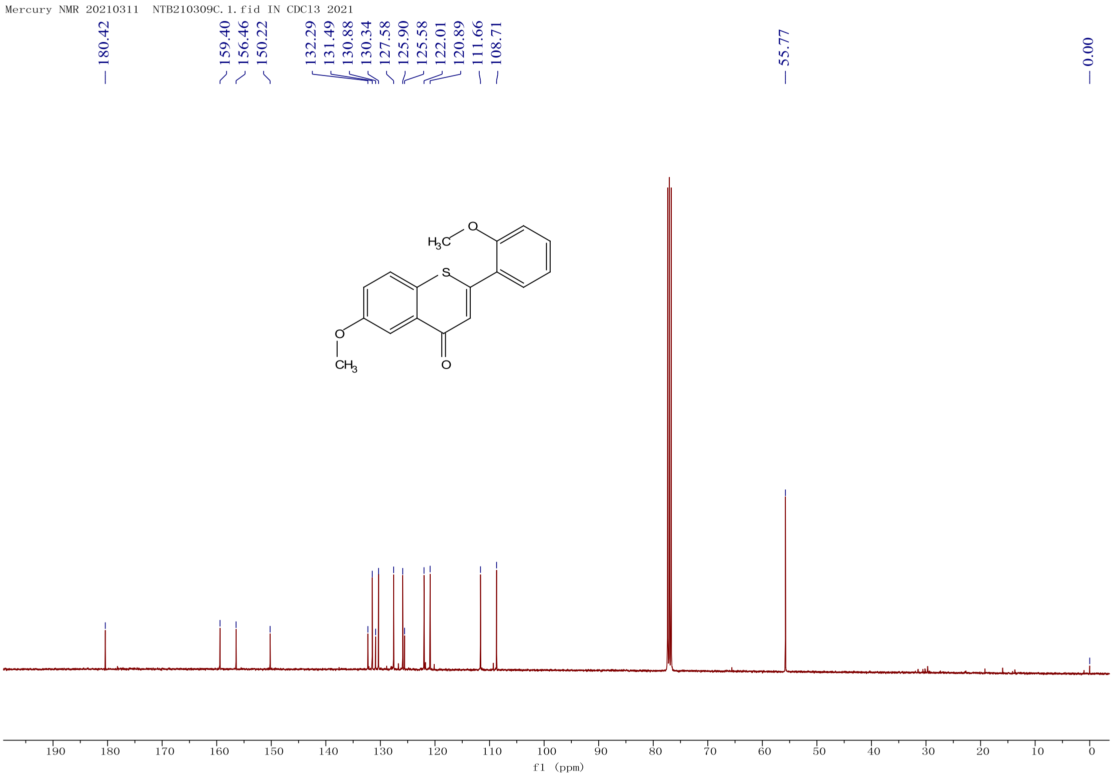

Figure S22. ^{1}H and ^{13}C NMR spectra of 6-Methoxy-2-(2-methoxyphenyl)-4*H*-thiochromen-4-one (**3v**)

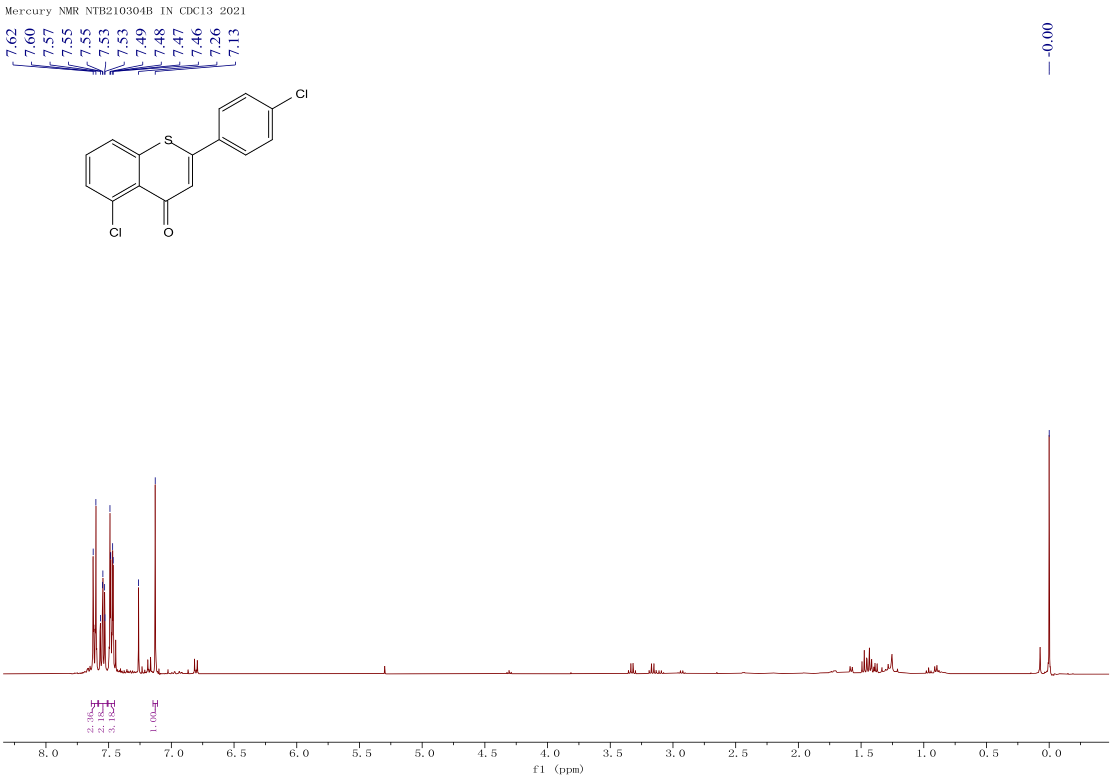

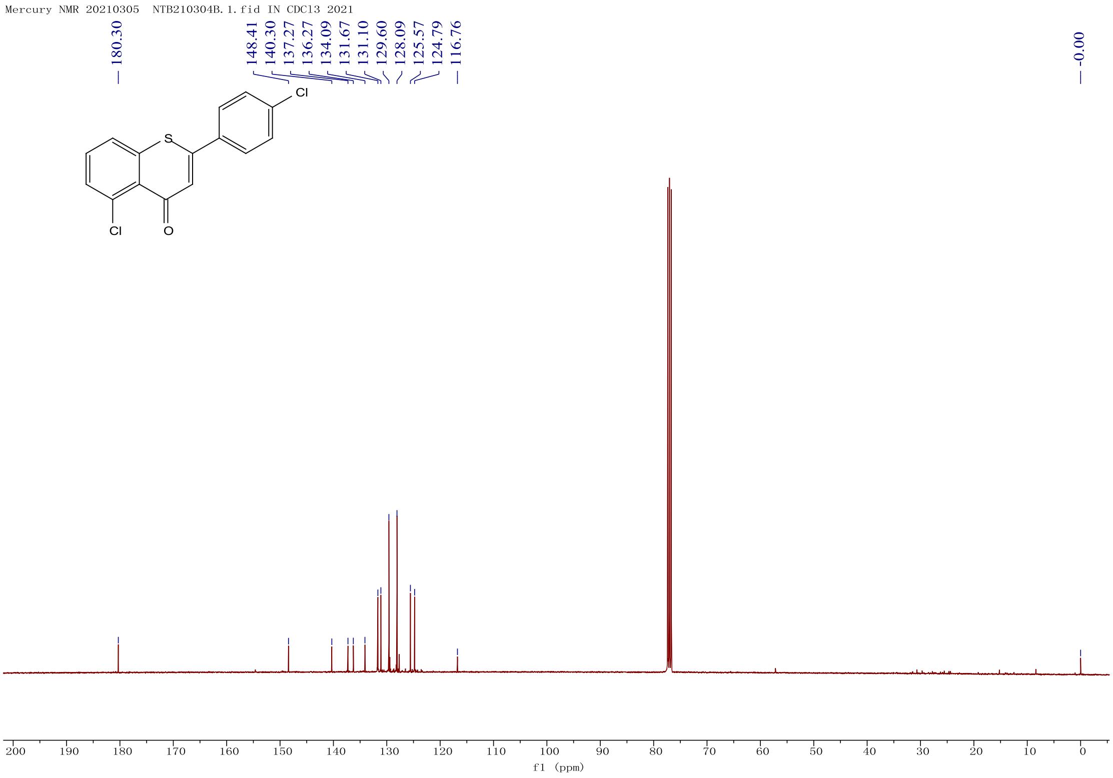

Figure S23. ^{1}H and ^{13}C NMR spectra of 5-Chloro-2-(4-chlorophenyl)-4*H*-thiochromen-4-one (**3w**)

Mercury NMR NTB210311B 1 IN CDCl3 2021

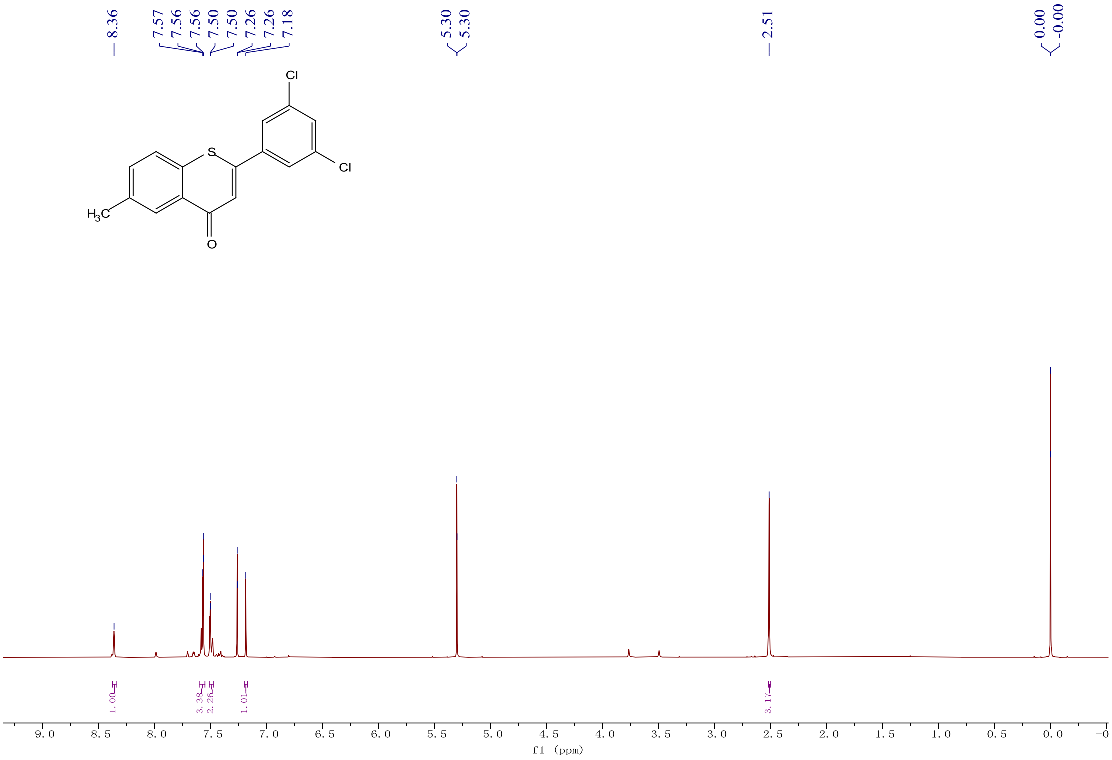

Mercury NMR 20210317 NTB210311B.1.fid IN CDCl3 2021

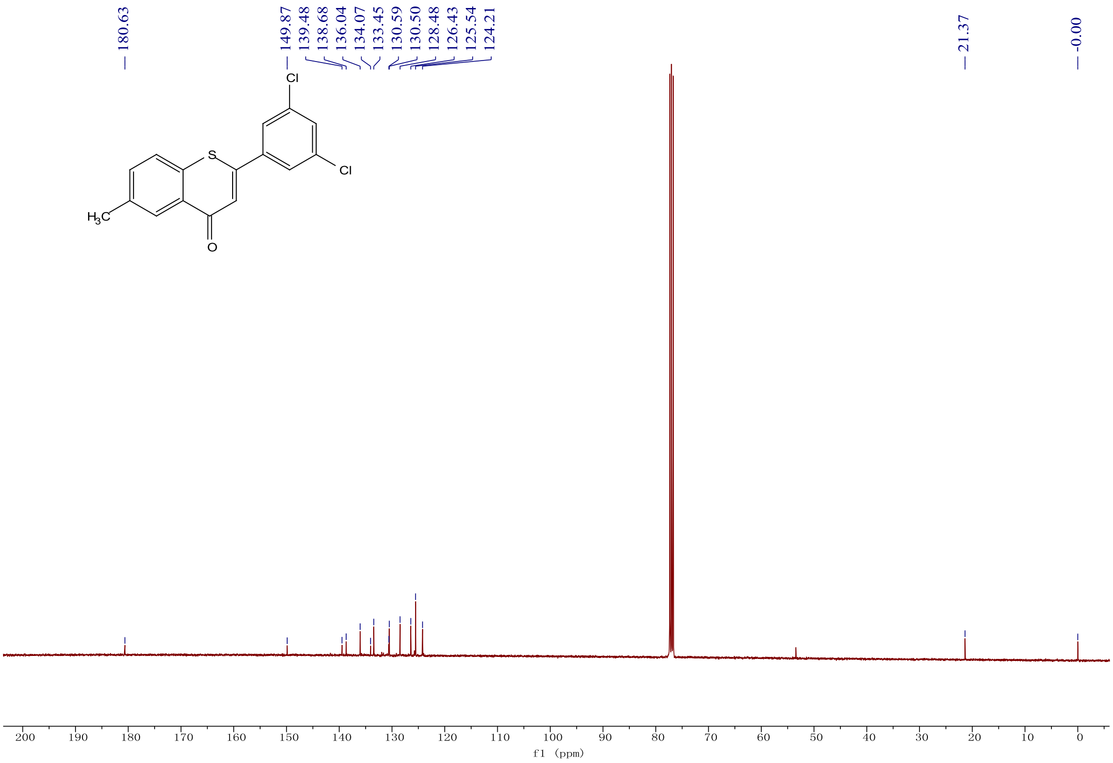

Figure S24. ^{1}H and ^{13}C NMR spectra of 2-(3,5-Dichlorophenyl)-6-methyl-4*H*-thiochromen-4-one (**3x**)

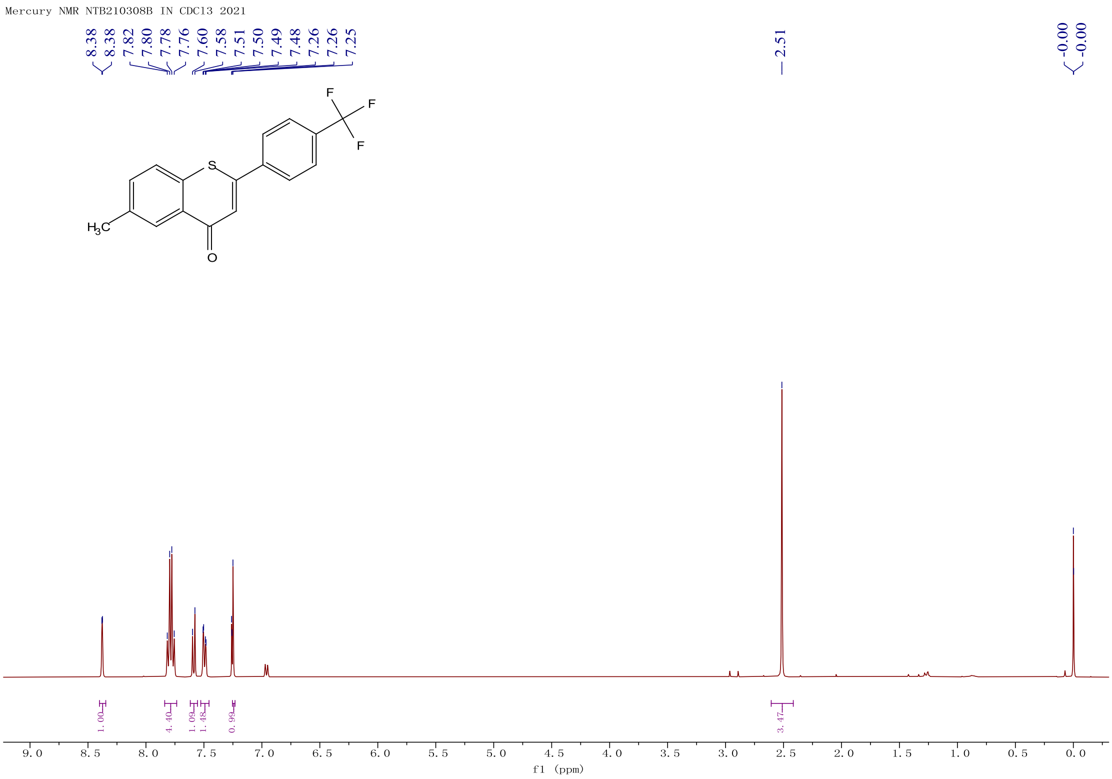

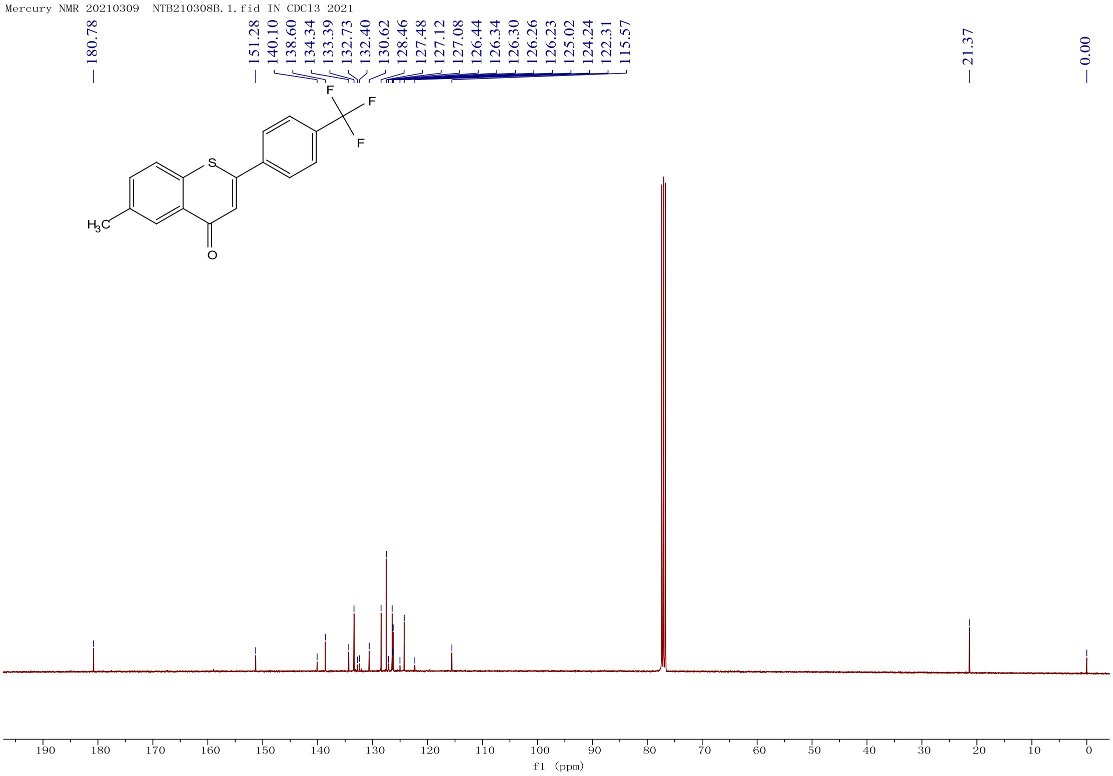

Figure S25. ^{1}H and ^{13}C NMR spectra of 6-Methyl-2-(4-(trifluoromethyl)phenyl)-4*H*-thiochromen-4-one (**3y**)

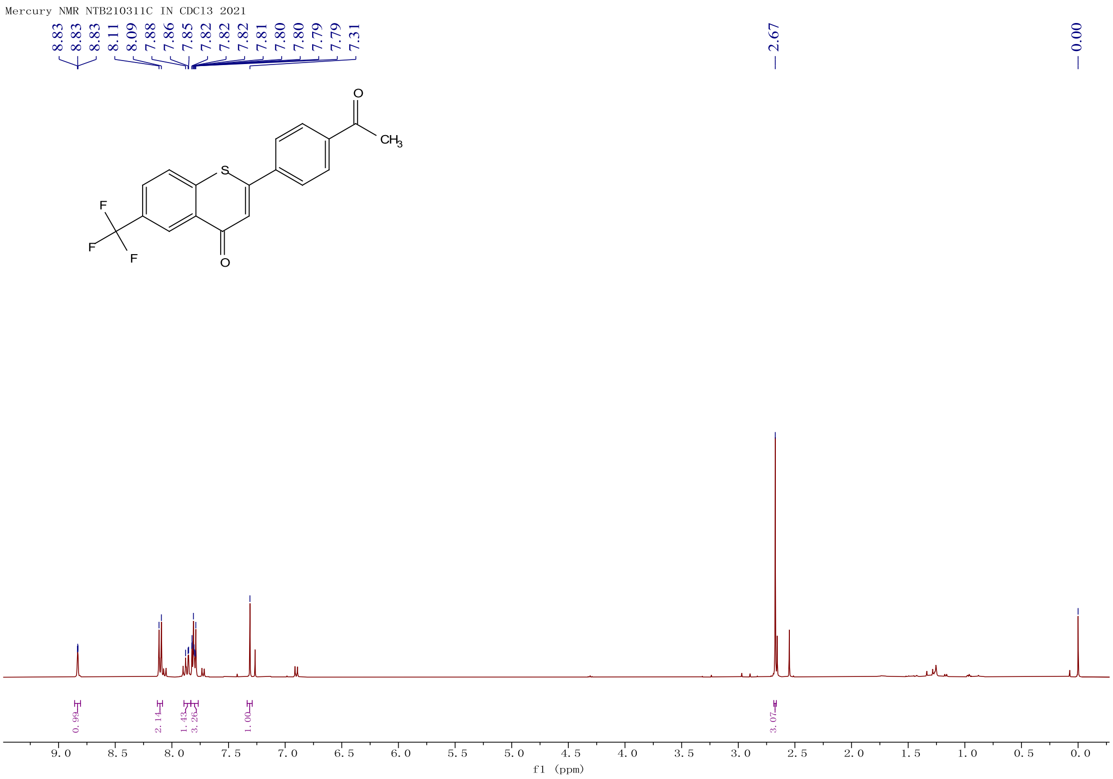

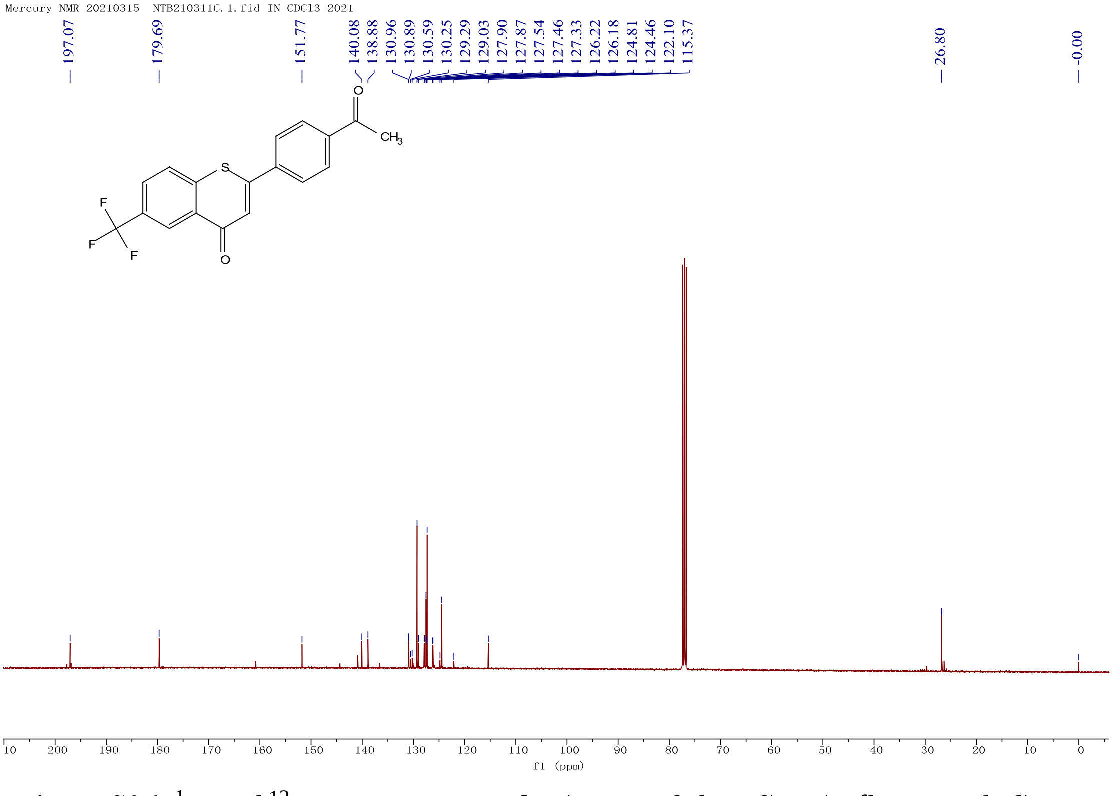

Figure S26. ^{1}H and ^{13}C NMR spectra of 2-(4-Acetylphenyl)-6-(trifluoromethyl)-4*H*-thiochromen-4-one (**3z**)

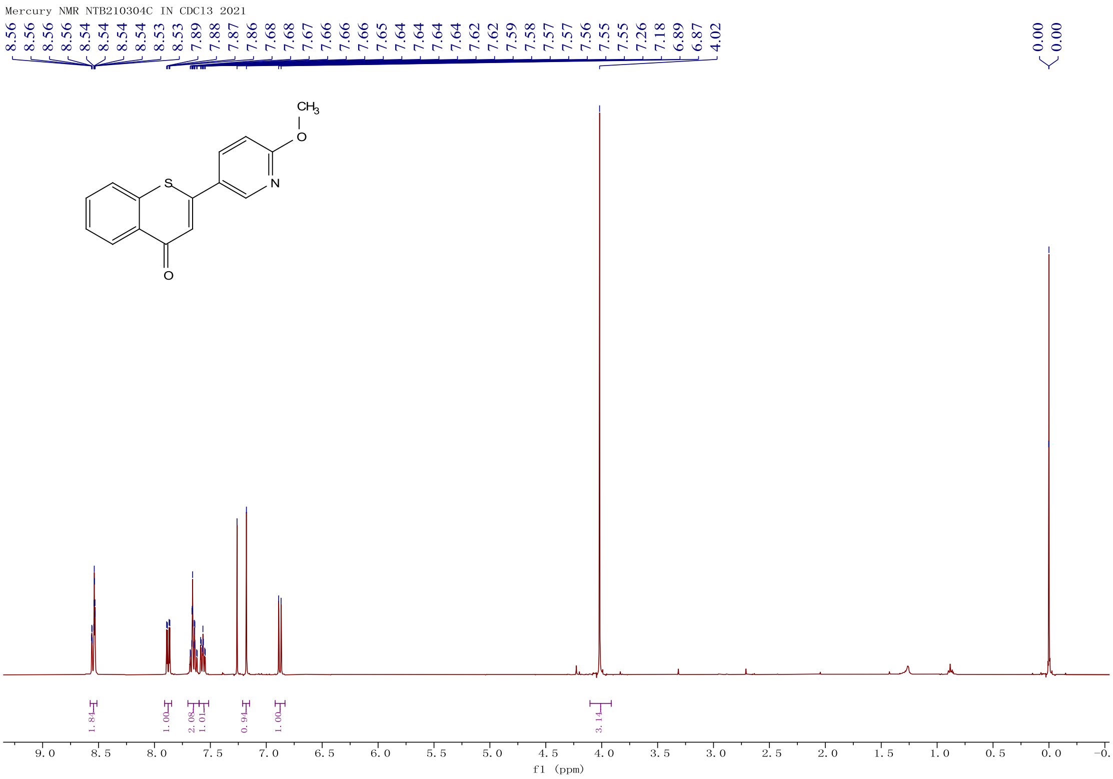

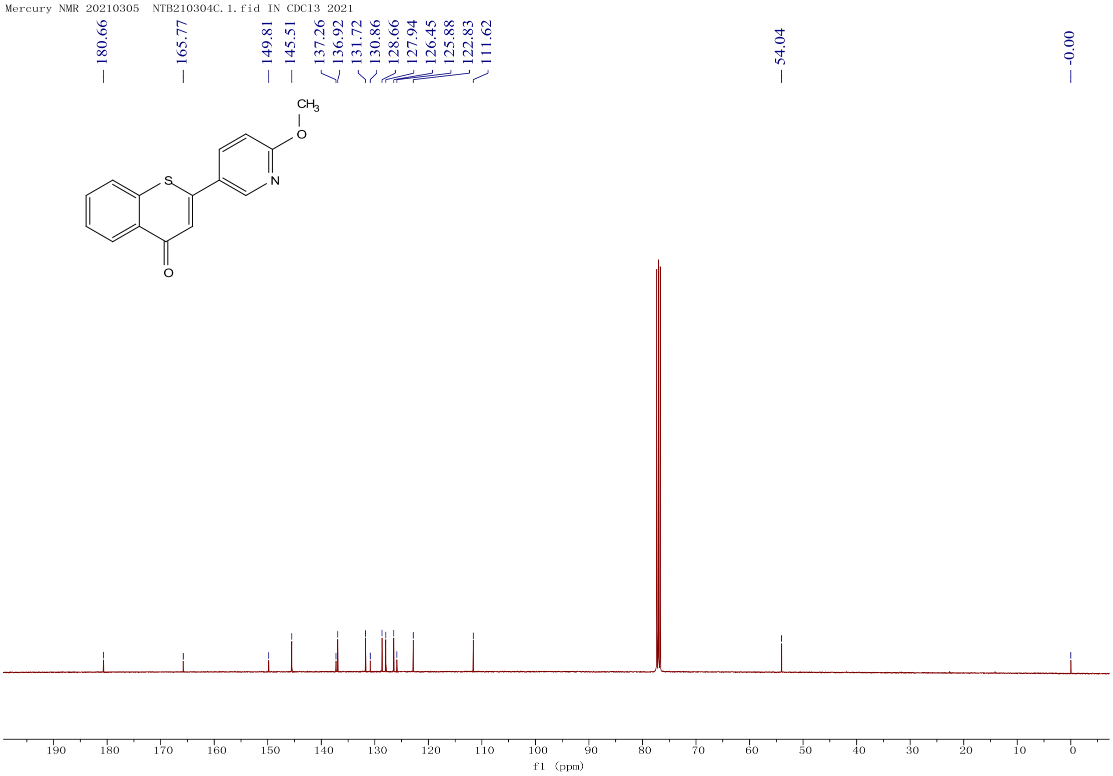

Figure S27. ^{1}H and ^{13}C NMR spectra of 2-(6-Methoxypyridin-3-yl)-4*H*-thiochromen-4-one (**4a**)

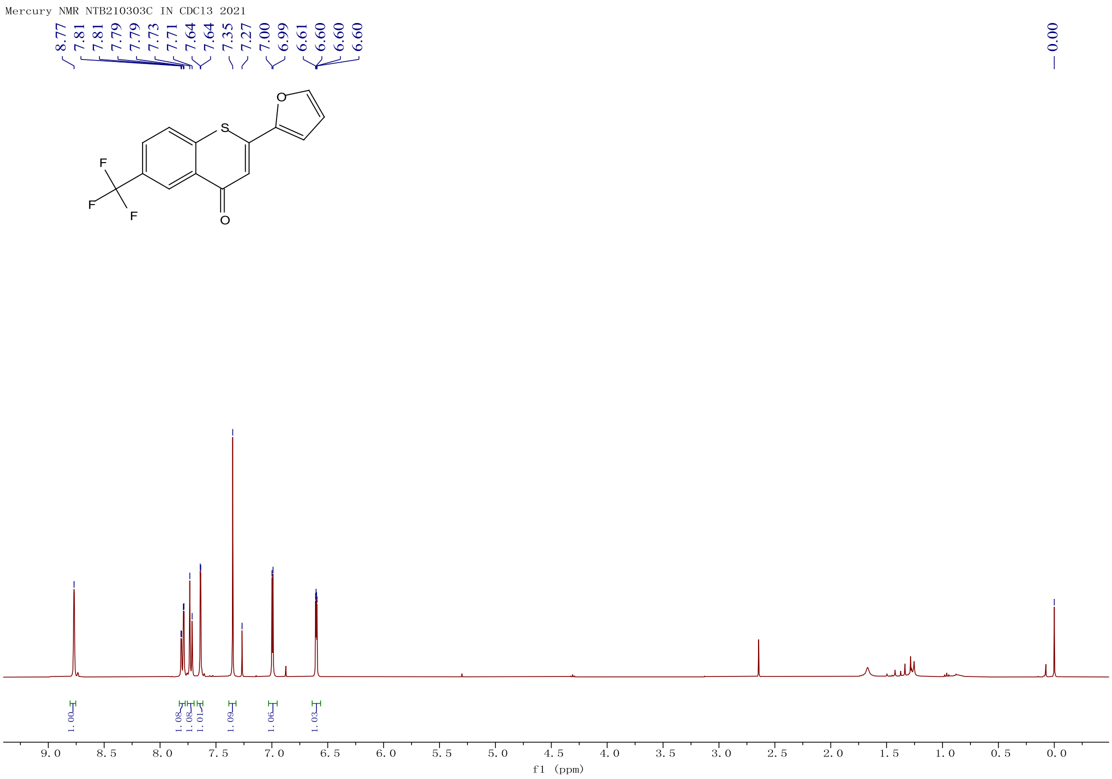

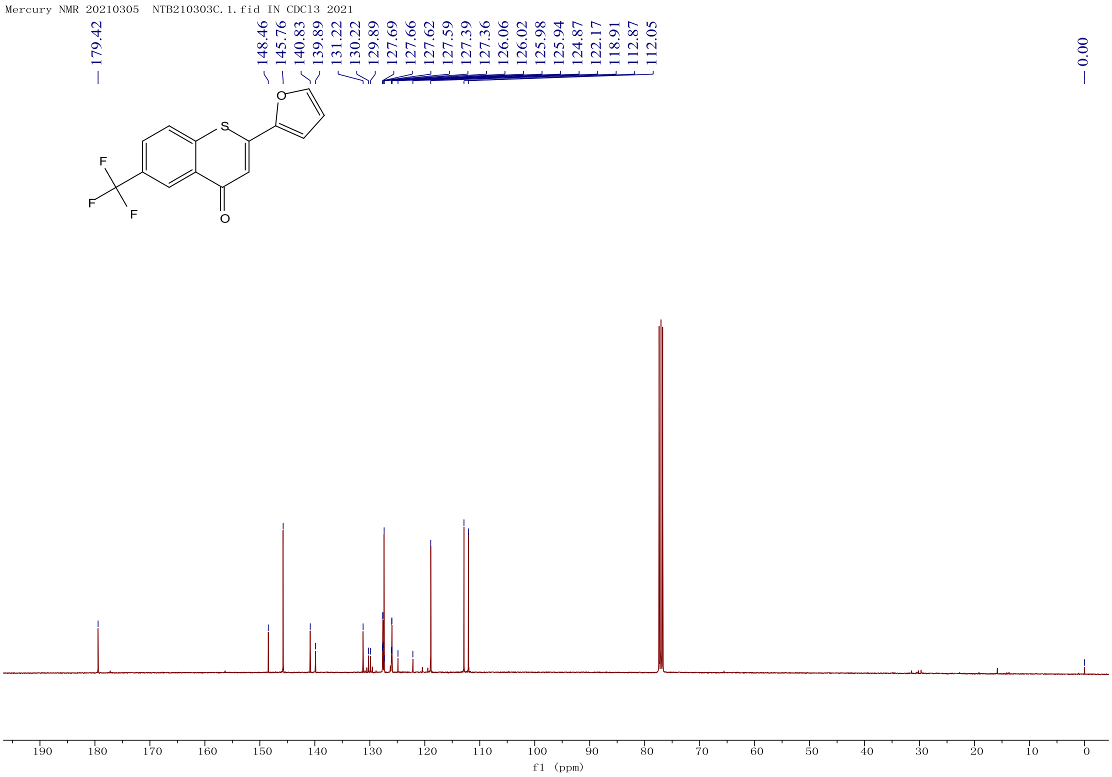

Figure S28. ^{1}H and ^{13}C NMR spectra of 2-(Furan-2-yl)-6-(trifluoromethyl)-4*H*-thiochromen-4-one (**4b**)

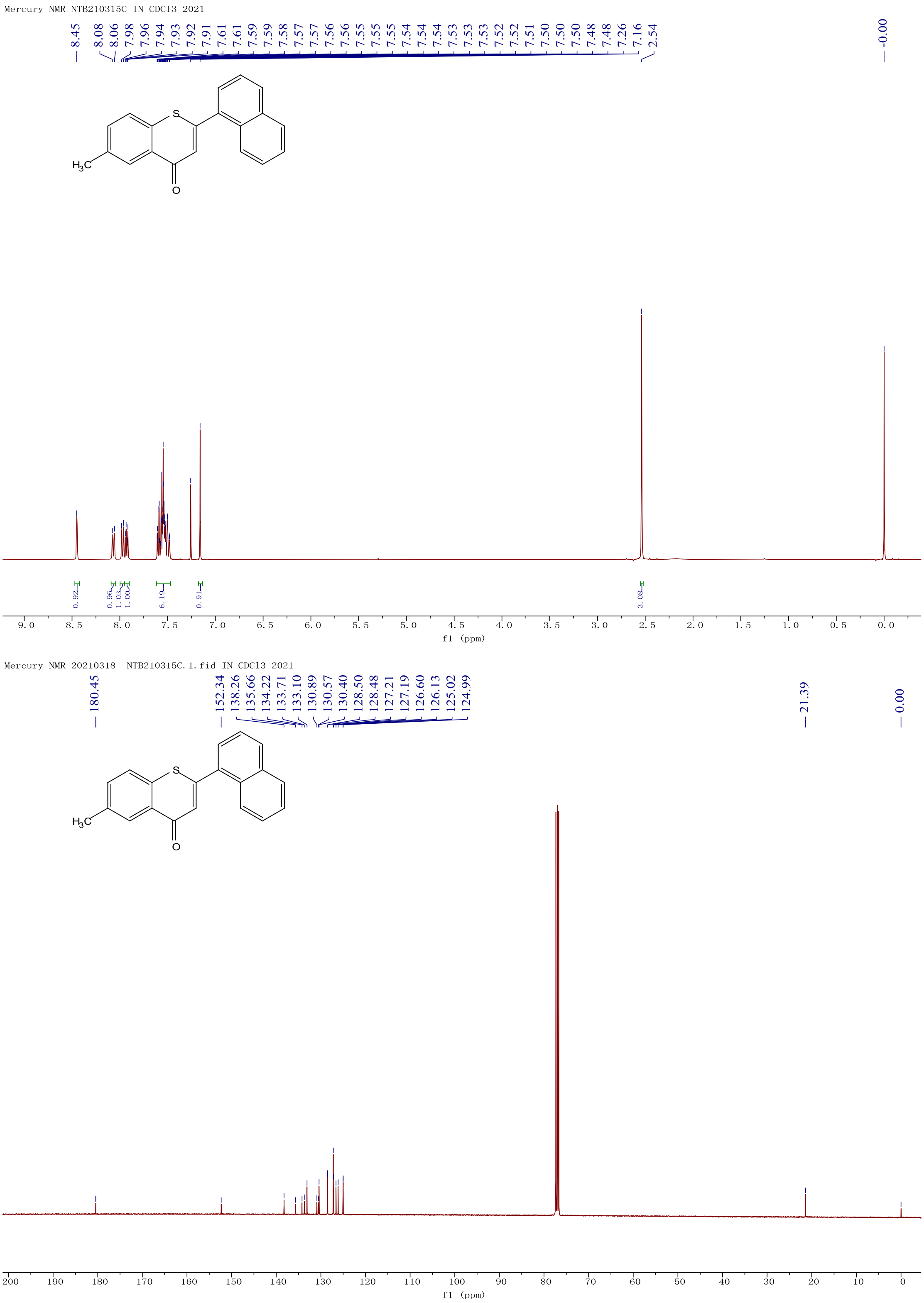

Figure S29. ^{1}H and ^{13}C NMR spectra of 2-(Benzo[*b*]thiophen-2-yl)-4*H*-thiochromen-4-one (**4c**)

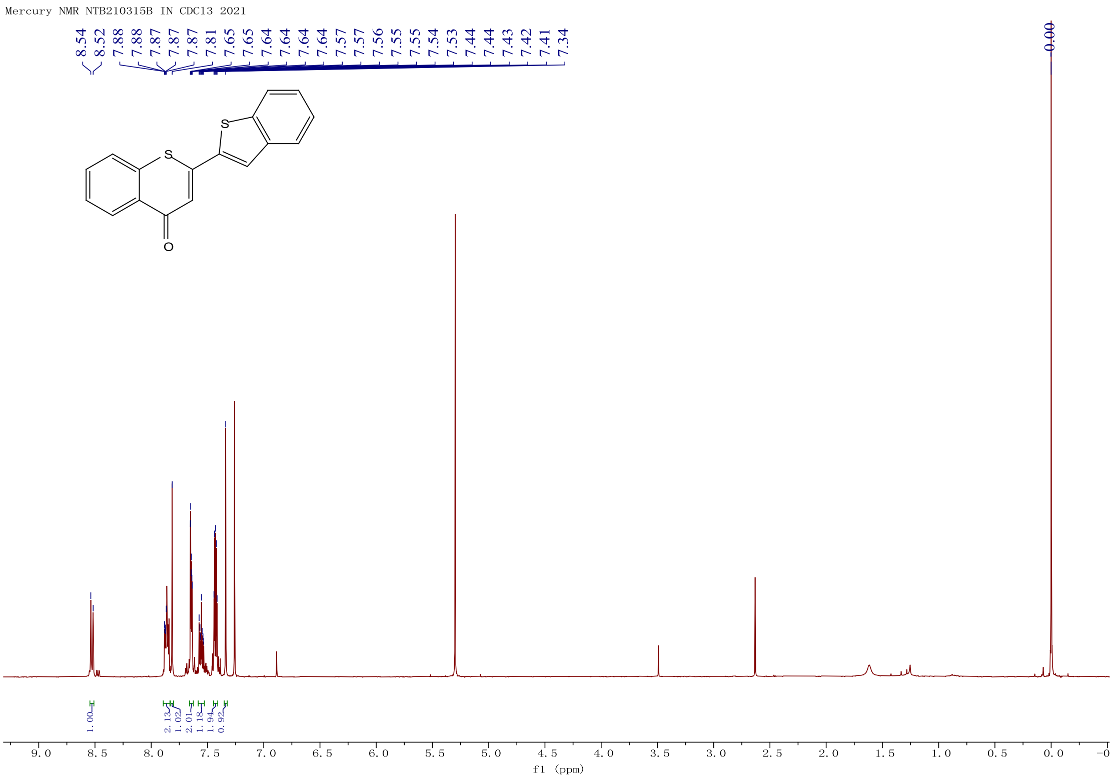

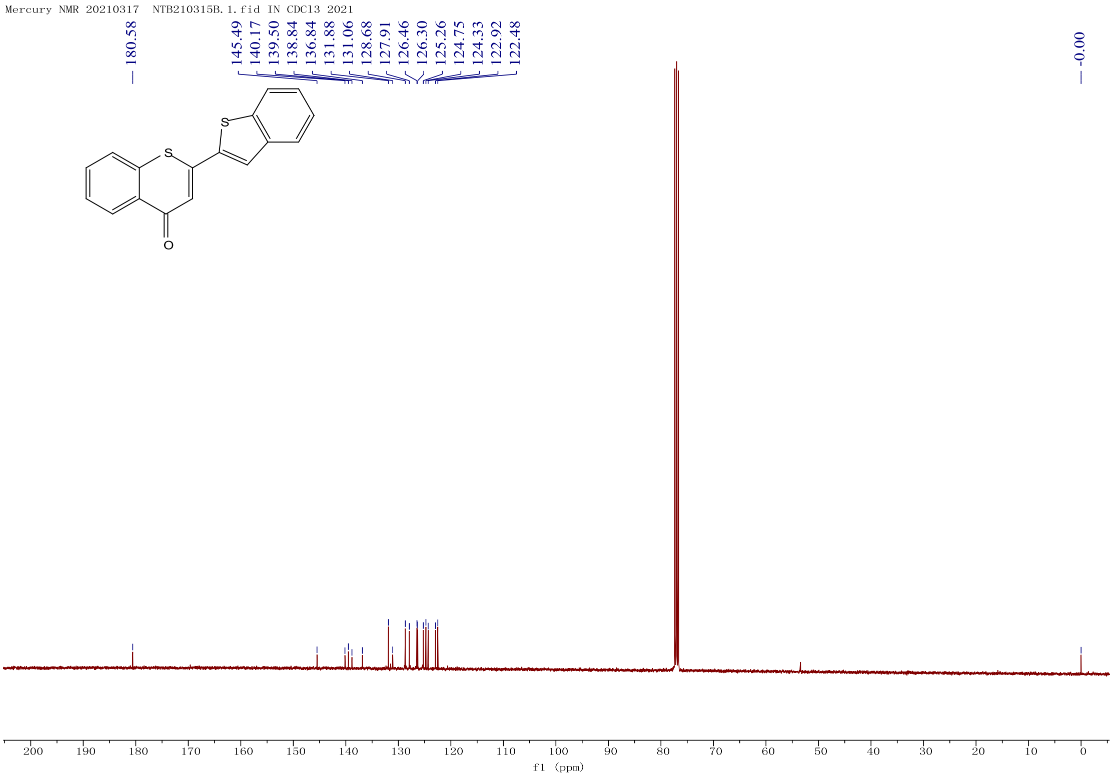

Figure S30. ^{1}H and ^{13}C NMR spectra of 6-Methyl-2-(naphthalen-1-yl)-4*H*-thiochromen-4-one (**4d**)
